# Calprotectin protects *Staphylococcus aureus* in coculture with *Pseudomonas aeruginosa* by attenuating quorum sensing and decreasing the production of pseudomonal antimicrobials

**DOI:** 10.1128/msystems.00576-25

**Published:** 2025-09-04

**Authors:** Wei H. Lee, Amanda G. Oglesby, Elizabeth M. Nolan

**Affiliations:** 1 Department of Chemistry, Massachusetts Institute of Technology 201648 , Cambridge, Massachusetts, USA; 2 Department of Pharmaceutical Sciences, School of Pharmacy, University of Maryland 733678 , Baltimore, Maryland, USA; 3 Department of Microbiology and Immunology, School of Medicine, University of Maryland 200790, Baltimore, Maryland, USA; University of Connecticut, Storrs, Connecticut, USA

**Keywords:** *Pseudomonas aeruginosa*, *Staphylococcus aureus*, coculture, calprotectin, metal availability, gene expression

## Abstract

*Pseudomonas aeruginosa* and *Staphylococcus aureus* cause debilitating polymicrobial infections in diverse patient populations. Studies of these bacterial pathogens in coculture have shown that environmental variables, including Fe availability and the host-defense protein calprotectin (CP), impact coculture dynamics. To decipher how CP modulates interactions between *P. aeruginosa* and *S. aureus*, we employed dual-species RNA-seq to examine the transcriptional responses of both pathogens in coculture to CP treatment and metal depletion. Analysis of these responses revealed that, for both *P. aeruginosa* and *S. aureus*, CP treatment not only induced gene expression changes consistent with single- and multi-metal starvation responses but also induced gene expression changes that were not observed under metal limitation. For *P. aeruginosa*, CP treatment induced gene expression changes pointing to a shift in chorismate flux away from alkylquinolone and phenazine biosynthesis and toward folate biosynthesis. These observations were consistent with decreased production of alkylquinolones by *P. aeruginosa*, including the potent anti-staphylococcal alkylquinolone N-oxides. CP treatment altered the levels of two quorum-sensing molecules, 3-oxo-C_12_-homoserine lactone and C_4_-homoserine lactone, produced by *P. aeruginosa*. In addition, CP treatment enhanced the ability of *S. aureus* to mount Fe-starvation responses and caused *S. aureus* to express host virulence genes. This analysis illuminated the physiological consequences of CP treatment that extend beyond metal starvation and impact interspecies interactions. Our findings provide a working model in which CP effectively disarms *P. aeruginosa* by inhibiting the production of anti-staphylococcal factors and boosts the ability of *S. aureus* to protect itself from attack.

**IMPORTANCE:**

The innate immune protein calprotectin (CP) defends the host against bacterial pathogens by sequestering multiple essential nutrient metal ions at infection sites. In addition to this role in nutritional immunity, CP promotes the survival of *Staphylococcus aureus* in coculture with *Pseudomonas aeruginosa*, an effect that is independent of its metal-sequestering function. In this work, we sought to understand how CP modulates this interspecies interaction by evaluating the transcriptional responses of *P. aeruginosa* and *S. aureus* to CP and metal limitation in cocultures. Our study revealed that CP attenuates the ability of *P. aeruginosa* to attack *S. aureus* with anti-staphylococcal factors and enhances the capacity of *S. aureus* to withstand this assault, effects that are not recapitulated by metal limitation. This work provides a new understanding of how CP modulates microbial interactions that are relevant to human health.

## INTRODUCTION

*Pseudomonas aeruginosa* and *Staphylococcus aureus* are two bacterial pathogens of clinical concern owing to their widespread prevalence, ability to colonize and thrive within the host environment, and resistance against available antimicrobial therapies (1–3). *P. aeruginosa* and *S. aureus* share infection niches, including chronic wounds and the lungs of cystic fibrosis (CF) patients (4, 5). Co-infections by *P. aeruginosa* and *S. aureus* exacerbate the severity of the infection (6–8), and interactions between these two bacterial pathogens increase the tolerance of both *P. aeruginosa* and *S. aureus* to antibiotic treatment (4, 5, 9–12). Elucidating how host immunity and the host environment impact interactions between these two bacterial pathogens is important for advancing fundamental understanding of coculture dynamics and pathogenesis.

*P. aeruginosa* and *S. aureus* interactions are antagonistic, with *P. aeruginosa* outcompeting *S. aureus* by secreting various anti-staphylococcal factors (13–15). Multiple virulence factors and exoproducts contribute to the anti-staphylococcal activity of *P. aeruginosa*, including LasA, LasB, and phenazines, such as pyocyanin (PYO) (16). Production of anti-staphylococcal factors by *P. aeruginosa* is controlled by a hierarchy of quorum-sensing (QS) metabolites, autoinducers, and their associated regulators (17–22). Two prominent autoinducer–regulator systems involved in this hierarchical QS cascade (23–26) are the 3-oxo-C_12_-HSL/LasIR (17, 27) and the C_4_-HSL/RhlIR systems (17, 28). When activated, the 3-oxo-C_12_-HSL/LasIR system triggers the activation of downstream virulence factors and systems (29–32), including the production of alkaline protease, staphylolysin (LasA) (33, 34), and upregulation of genes encoding the C_4_-HSL/RhlR system (18). Activation of the C_4_-HSL/RhlIR system is essential for rhamnolipid biosynthesis and the production of elastase (LasB) (35–37) and PYO (38). *P. aeruginosa* also produces multiple alkylquinolones (AQs) that contribute to its anti-staphylococcal activity both as QS molecules and as direct anti-staphylococcal metabolites (19, 39–42). The combination of multiple molecular factors drives *S. aureus* toward fermentative metabolism and reduces *S. aureus* viability (11, 14, 43, 44).

The host environment undoubtedly affects interactions between *P. aeruginosa* and *S. aureus*. One environmental variable is nutrient availability. *P. aeruginosa* and *S. aureus* have a metabolic Fe requirement, and the host lowers Fe availability to starve invading pathogens in a process termed nutritional immunity (45, 46). In response to host-imposed Fe limitation, *P. aeruginosa* and *S. aureus* express dedicated transporters (47, 48), siderophores (49–52), and heme uptake machinery (53, 54) to compete for Fe. Prior studies of *P. aeruginosa* and *S. aureus* cocultures have examined the importance of metal availability, revealing that Fe starvation enhances anti-staphylococcal activity of *P. aeruginosa* toward *S. aureus* (55, 56). The host protein calprotectin (CP) contributes to nutritional immunity by sequestering multiple divalent transition metal ions, including Fe(II), and elicits single- and multi-metal starvation responses in these two bacterial pathogens (57–63). CP was also shown to promote *S. aureus* survival in coculture with *P. aeruginosa*, which was attributed to reduced production of anti-staphylococcal factors resulting from CP-mediated metal limitation (64). Combined, these studies revealed an apparent dichotomy in the field, wherein CP sequesters Fe(II) and induces Fe-starvation responses in both pathogens, while also providing a protective effect on *S. aureus* when cocultured with *P. aeruginosa* (64–66). Our recent work demonstrated that the protective effect of CP on *S. aureus* is metal-independent, indicating that the CP protein scaffold directly impacts coculture dynamics (66). Our findings also suggested that perturbed production of PYO and the siderophore pyochelin (PCH) by *P. aeruginosa* in the presence of CP may arise due to additional effects of CP that extend beyond its metal sequestering ability (66). Others have shown that CP interacts physically with *P. aeruginosa* and *S. aureus* during coculture (67), though the impact of these interactions on the anti-staphylococcal activity of *P. aeruginosa* and the viability of *S. aureus* is unknown. Taken together, these studies illustrate the complex and multifactorial effects of the CP protein scaffold and metal limitation on coculture outcomes, necessitating a new model for how CP and metal availability modulate interspecies dynamics between *P. aeruginosa* and *S. aureus*.

To support the development of such a model, we utilized dual-species RNA-seq to evaluate the global transcriptional responses of *P. aeruginosa* and *S. aureus* in coculture to CP treatment and metal depletion. We report that CP treatment elicits transcriptional responses in both bacterial pathogens that shape coculture outcomes and that were not observed in metal-depleted cocultures. In *P. aeruginosa*, CP treatment induced gene expression changes that indicated redirected chorismate flux, perturbed levels of QS effectors, and decreased production of AQs, effectively reducing the anti-staphylococcal activity of *P. aeruginosa*. Consistent with these observations, the presence of CP led to decreased gene expression responses by *S. aureus* related to membrane damage and cell stress while enhancing Fe-starvation responses. CP treatment also increased the expression of *S. aureus* genes associated with host virulence. Our findings support a model in which *P. aeruginosa* functions as an attacker that antagonizes *S. aureus*, the defender, primarily through the action of alkylquinolone N-oxides. By perturbing QS production and decreasing AQ production, CP effectively disarms *P. aeruginosa* and promotes the survival of *S. aureus* in coculture. Collectively, our results show that the CP protein scaffold markedly impacts coculture dynamics between these two pathogens, demonstrating that components of host immunity may impact pathogen–pathogen interactions in ways outside their known function.

## RESULTS AND DISCUSSION

### Experimental considerations for dual-species RNA-seq

The study design leveraged insights from prior investigations into the effects of CP treatment and metal depletion on the growth dynamics of *P. aeruginosa* and *S. aureus* cocultures, utilizing the same coculture conditions as previously described (66). Briefly, *P. aeruginosa* strain UCBPP-PA14 (hereafter PA14) and *S. aureus* strain USA300 JE2 (hereafter JE2) were grown in a chemically defined medium (CDM) prepared from trace metal basis reagents used in prior studies of both species in monocultures or cocultures (57–59, 66). This base medium is used to prepare media with defined metal concentrations. Metal-replete CDM is supplemented with 0.3 µM Mn, 5 µM Fe, 0.1 µM Ni, 0.1 µM Cu, and 6 µM Zn. These metal concentrations are representative of physiologically relevant metal levels in sputum samples from CF patients (68, 69). The omission of one or more metals (Mn, Fe, Zn, or all three metals) from this mixture allows for comparisons between the effects of CP treatment and either single- or multi-metal depletion on *P. aeruginosa* and *S. aureus* in monoculture and coculture. We performed dual-species RNA-seq on cocultures of these two bacterial pathogens grown in metal-replete, Mn-depleted, Fe-depleted, Zn-depleted, metal-depleted CDM (depleted of Mn, Fe, and Zn), and metal-replete CDM supplemented with a physiologically relevant concentration of CP (20 µM) (66, 70, 71). We compared the transcriptional responses of the cocultures exposed to metal depletion with those observed for CP treatment. We also performed RNA-seq on *P. aeruginosa* and *S. aureus* monocultures treated with CP (20 µM) to identify transcriptional responses occurring specifically in coculture or monoculture of either species, as well as responses common to both culture types.

We identified the 6–8-h period as a potential window for sample collection based on our prior coculture growth and metabolite time-course studies. Fe-starvation responses, including appreciable production of the pseudomonal siderophores pyoverdine and PCH and decreased production of phenazines, occurred from 6 h onward in cultures treated with CP (66). To determine the appropriate time point for RNA-seq sample collection, real-time PCR was used to confirm sample reproducibility by validating consistent transcript levels for both *P. aeruginosa* and *S. aureus* cocultured in Fe-depleted medium and metal-replete medium with or without CP (66). Across the conditions tested, *P. aeruginosa* RNA abundance remained high as judged from transcript levels of the housekeeping gene 16S. However, the reproducibility of *S. aureus* RNA transcripts from cocultures grown in the absence of CP declined significantly past the 6-h time point as judged from highly variable and often trace levels of the *S. aureus* housekeeping gene *sigA* (Table S1), indicative of significant RNA degradation as *P. aeruginosa* anti-staphylococcal activity proceeded. Consequently, the 6-h time point was selected for RNA-seq. For differential expression analysis, metal-replete CDM functioned as the (untreated) control. The proportions of differentially expressed (DE) genes for *P. aeruginosa* and *S. aureus* are presented in Table S2. Key statistics of the RNA-seq data set, including the sequenced read count and the number of unique features sequenced (72), are presented in Table S3. Herein, we summarize multi-metal starvation responses induced by CP treatment in cocultures of *P. aeruginosa* and *S. aureus* and describe how CP treatment modulates interspecies dynamics between both bacterial pathogens. Additional transcriptional responses of *P. aeruginosa* unique to each experimental condition are presented in the accompanying supplemental Discussion. The complete list of DE *P. aeruginosa* and *S. aureus* genes is presented in the accompanying supplemental files.

### CP elicits multi-metal starvation responses in *P. aeruginosa* cocultured with *S. aureus*

**Fig 1:**
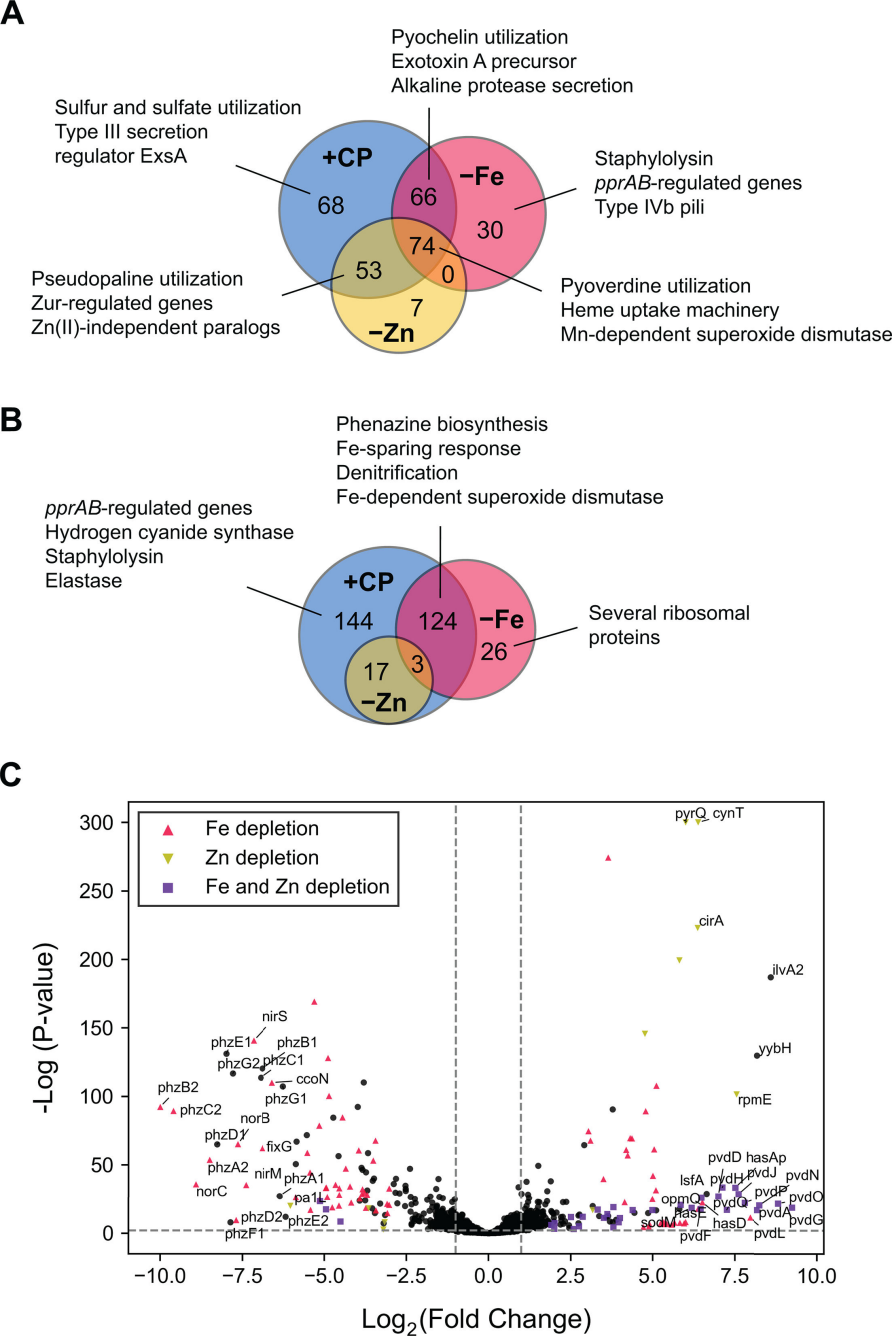
CP treatment induces multi-metal starvation responses in *P. aeruginosa* when cocultured with *S. aureus*. Venn analyses reveal significant overlap of upregulated (**A**) and downregulated (**B**) *P. aeruginosa* genes in cocultures treated with CP and cocultures grown in Fe-depleted or Zn-depleted CDM. The top 600 DE genes across all culture conditions were used for Venn analyses. (**C**) Volcano plot of DE changes in response to CP treatment. Genes with similar DE patterns in response to Fe depletion, Zn depletion, or both Fe and Zn depletion are denoted as colored shapes. A threshold cutoff log_2_(fold change) of 1 was employed. The complete list of DE *P. aeruginosa* genes identified in each condition is presented in Tables SF1 to SF10.

We examined the top 600 DE genes across all culture conditions with Venn analysis to compare responses resulting from CP treatment and metal depletion. For *P. aeruginosa* genes DE in response to CP treatment and Fe depletion, about 48% of upregulated genes (Fig. 1A) and 40% of downregulated genes (Fig. 1B) were common to both conditions, demonstrating significant overlap. For genes DE in response to CP treatment and Zn depletion, 47% of upregulated genes (Fig. 1A) and all downregulated genes (Fig. 1B) were common to both conditions. For cocultures grown in Mn-depleted CDM, only 42 *P*. *aeruginosa* genes fell within the top 600 DE genes across all culture conditions (Fig. S1A and B), and nearly all genes observed to be upregulated in response to Mn depletion were also upregulated in CP-treated cocultures (Fig. S1A). Transcriptional responses common to CP treatment and the depletion of Fe, Zn, or Mn were also recapitulated in metal-depleted cocultures (Fig. S1 to S3). While most transcriptional responses (~87%) of *P. aeruginosa* in monoculture to CP treatment overlapped with the transcriptional responses for *P. aeruginosa* in coculture to CP treatment, approximately 9% of upregulated genes and 16% of downregulated genes were found to be unique to either monoculture or coculture (Fig. S4A and B).

To further probe similarities and differences between CP treatment and metal depletion, we performed functional enrichment analyses. Overrepresentation analysis of DE genes revealed considerable overlap between the effects of CP treatment and metal depletion for *P. aeruginosa* in coculture (Fig. S5A and C), but also identified several distinct clusters of Gene Ontology (GO) terms responding uniquely to CP treatment or the depletion of Fe, Mn, or Zn (Fig. S5B and D). Genes encoding the Mn-dependent superoxide dismutase SodM (73), the heme acquisition protein HasAp (74), Phu heme uptake machinery (53), and pyoverdine biosynthesis and uptake machinery (*pvd* operon) (50, 66) were upregulated in all four conditions (Table SF1A). No systems were downregulated in all four conditions (Table SF1B). Collectively, our findings demonstrate that CP treatment elicits multi-metal starvation responses in *P. aeruginosa* cocultured with *S. aureus*, and that these responses overlap considerably, but not fully, with the effects of metal depletion.

### CP treatment induces Fe-starvation responses in *P. aeruginosa* cocultured with *S. aureus*

**Fig 2:**
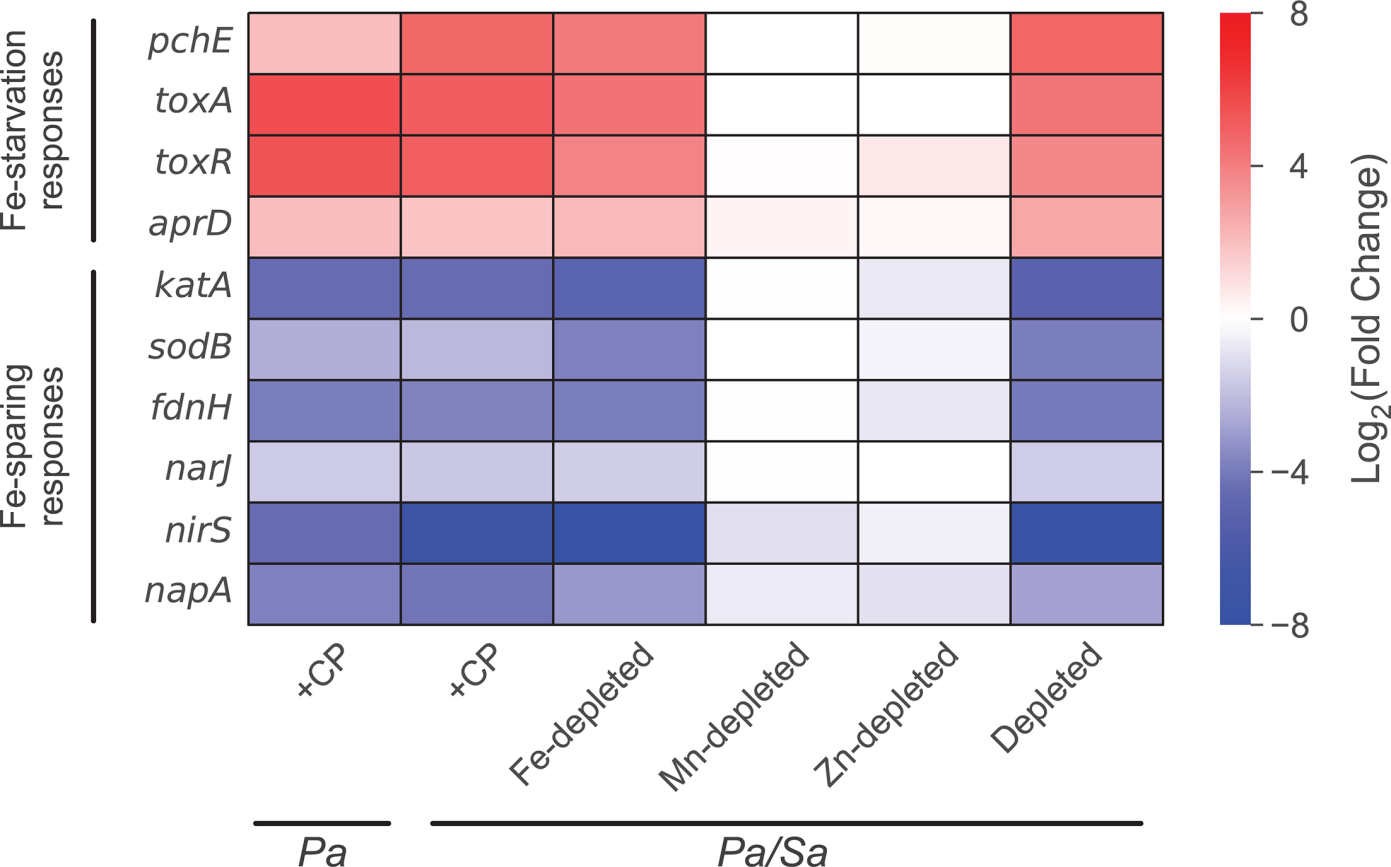
CP elicits Fe-starvation responses and Fe-sparing responses by *P. aeruginosa* cocultured with *S. aureus*. DE heatmap of *P. aeruginosa* genes associated with Fe-starvation responses and Fe-sparing responses. *Pa* indicates *P. aeruginosa* monoculture, and *Pa/Sa* indicates the coculture.

In agreement with prior studies reporting that CP elicits transcriptional changes associated with Fe-starvation responses in *P. aeruginosa* during coculture with *S. aureus* (66) and in monoculture (57, 59, 64), CP treatment and growth in Fe-depleted CDM resulted in the upregulation of genes encoding pyoverdine (*pvd*) and pyochelin (*pch*) biosynthetic machinery (51*)*, the exotoxin A precursor (toxA*)* (64, 75), the transcriptional regulator (*toxR*) (76), and alkaline protease secretion machinery (77) (Fig. 1A and 2; Table SF2A). These observations are also consistent with previous studies of Fe-starvation responses in *P. aeruginosa* (58, 78). Genes encoding the MexEF-OprN efflux pump, which secretes the QS metabolite HHQ (79, 80) and contributes to antimicrobial resistance (81), and the Fe-independent paralog of fumarase *fumC1* (82) were also found to be upregulated in response to CP treatment and Fe depletion (Table SF2A).

Under conditions of Fe limitation, *P. aeruginosa* decreases the expression of Fe-containing proteins via an Fe-sparing response that is mediated by PrrF small RNAs (sRNAs) (83–85). CP treatment and Fe depletion resulted in strong downregulation of genes for the Fur-regulated catalase (*katA*) (86, 87), the Fe-cofactored superoxide dismutase (*sodB*) (88), the nitrate-inducible formate dehydrogenase (*fdnIHG*) (89, 90), and denitrification (*nar*, *nir*, and *nap*), indicative of Fe-sparing responses under these treatment conditions (91) (Fig. 1B and 2; Table SF2B). Furthermore, CP treatment and Fe depletion decreased the expression of genes for the cbb3-type cytochrome c oxidase (*cco*) (64, 92–94) and NADH dehydrogenase (*nuo*) (95) (Fig. S6; Table SF2B) as part of an Fe-starvation response (78). CP treatment and Fe depletion also decreased the expression of genes for the bacterial ferritin (*ftnA*) (96) and phenazine biosynthetic machinery (see below) (Fig. S7).

### CP treatment induces Zn-starvation responses in *P. aeruginosa* cocultured with *S. aureus*

Consistent with Zn-starvation responses (58), CP treatment and Zn depletion resulted in the upregulation of genes associated with Zn uptake machinery, including pseudopaline biosynthesis and transport (*cnt*) (97, 98), the *znu* operon (99, 100)*,* and the Zn uptake regulator *zur* (99) (Fig. 1A; Fig. S8; Table SF3A). Furthermore, expression of genes for the Zn(II)-independent paralogs of RpmE and RpmJ (*PA14_17700–PA14_17710*) (58) and a gene cluster for a predicted Zn(II) uptake system (*PA14_26390–PA14_26420*) (100) were upregulated upon CP treatment and Zn depletion (Fig. 1A; Fig. S8). In addition, a Zur-regulated cluster of Zn-independent paralogs (*PA14_72980–PA14_73070*) (58, 100) was among the most strongly upregulated hits in CP-treated and Zn-depleted cocultures (Table SF3A). This cluster contained genes for the cell wall amidase (*amiA*) (101, 102), a putative carbonic anhydrase (*cynT*) (58), and the Zn-independent transcription factor (*dksA2*) (103, 104) (Fig. S8). Genes downregulated by CP treatment and Zn depletion included several that are known to be regulated by the PprAB two-component system (105) (see below) (Table SF3B). Unexpectedly, we found that expression of genes for the ferrous iron uptake system (*feoAB*) (47, 58, 106) and the catecholate siderophore receptor (*cirA*) (107, 108) was upregulated in CP-treated and Zn-depleted conditions but not in Fe-depleted conditions (Fig. S8).

### CP treatment does not induce Mn-starvation responses in *P. aeruginosa* cocultured with *S. aureus*

We detected no DE of genes encoding the putative Mn uptake proteins MntH1 and MntH2 or genes encoding proteins known to be Mn-cofactored in CP-treated cocultures (58) (Table SF4). These findings are consistent with prior studies reporting that CP treatment had a negligible effect on cell-associated Mn levels in *P. aeruginosa* PAO1 grown under aerobic conditions (59).

### CP treatment induces gene expression changes associated with cell envelope modifications in *P. aeruginosa* cocultured with *S. aureus*

**Fig 3:**
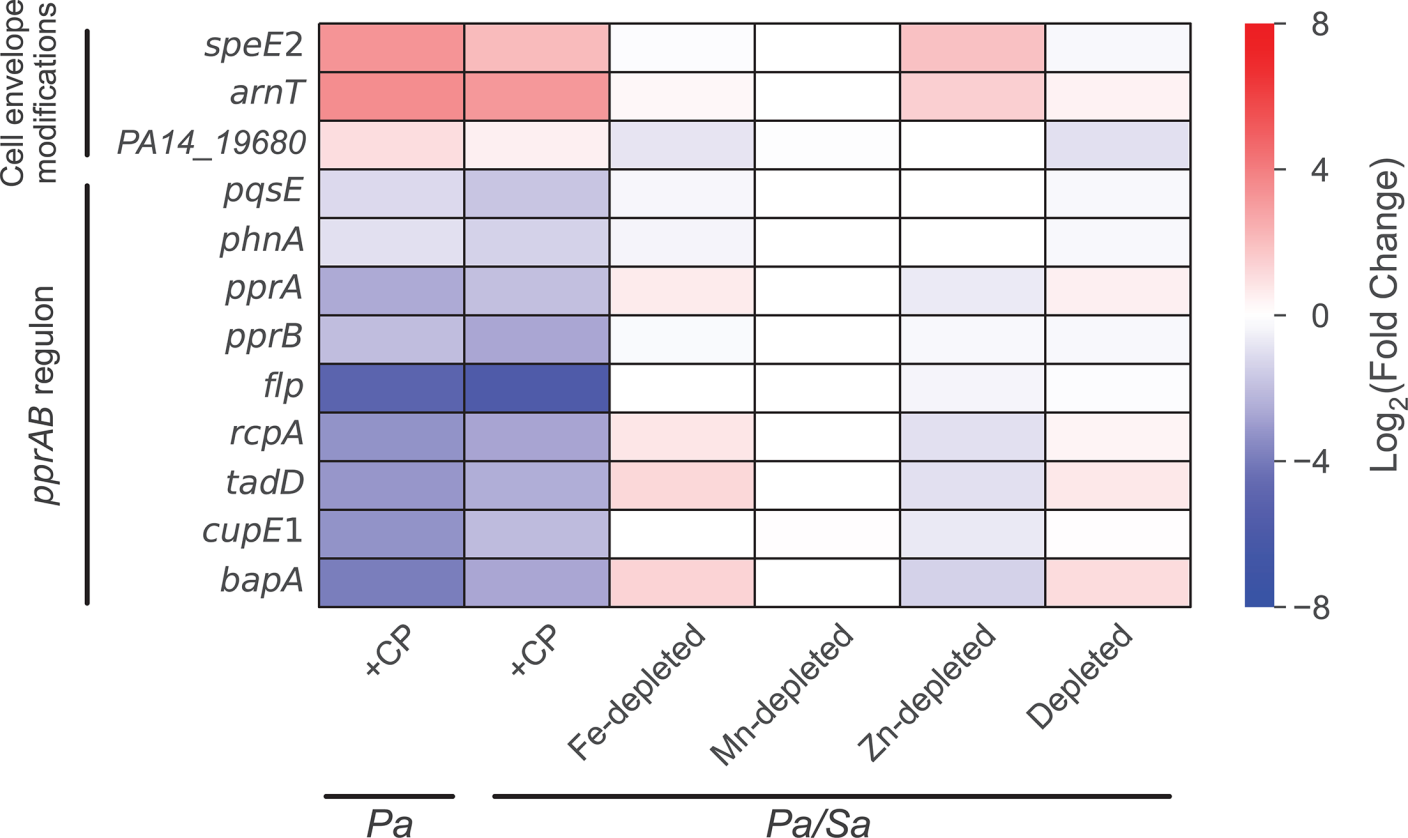
CP elicits transcriptional responses associated with cell envelope modifications for *P. aeruginosa* in coculture with *S. aureus*. DE heatmap of *P. aeruginosa* genes associated with cell envelope modifications and the *pprAB* regulon. *Pa* indicates *P. aeruginosa* monoculture, and *Pa/Sa* indicates the coculture.

Having characterized the aforementioned metal-starvation responses of *P. aeruginosa* to CP treatment, we looked at functional categories of transcriptional changes that could not be fully accounted for by metal depletion. The presence of CP resulted in DE of multiple systems associated with modifications to the cell envelope of *P. aeruginosa* in monoculture and in coculture with *S. aureus*. These gene expression changes were mostly absent for cocultures grown in Fe-depleted conditions. In agreement with prior work (66), CP treatment upregulated the expression of genes encoding the spermidine synthase SpeE2 (109, 110), the 4-amino-4-deoxy-l-arabinose lipid A transferase ArnT (111, 112), and the holin CidA (*PA14_19680*), although the change in the expression of *cidA* fell below the DE threshold (Fig. 3). CP treatment resulted in the downregulation of genes encoding the PprAB two-component system (Fig. 3), which regulates a hyper-biofilm phenotype (Pel- and Psl-independent) in *P. aeruginosa* (105). Furthermore, genes known to be positively regulated by PprAB (105), including those encoding structural components and assembly of the type IVb pili (113, 114), CupE fimbriae (115), and the BapA adhesin (105, 116), were downregulated by CP treatment (Fig. 3). We observed that downregulation of genes associated with the PprAB regulon was partially attributable to Zn depletion. CP treatment also upregulated the expression of the *arn* operon and the *PA14_63110–PA14_63160* locus encoding the two-component sensor/regulator PmrAB (117, 118), transcriptional responses that were common to Zn-depleted cocultures (Table SF3A). The PprAB two-component system has also been reported to regulate genes encoding PQS biosynthetic and transport machinery (*pqsCDE*) (see below) and anthranilate synthase (*phnAB*) (105) (Fig. 3).

We also found that CP elicited transcriptional changes that were not attributable to metal depletion. CP treatment caused mild upregulation of *cprRS* (119) and *parRS* (120) encoding two-component systems (Fig. S9) that are associated with *P. aeruginosa* responses to cationic peptides and antibacterials, including aminoglycosides and polymyxins. Additional indications of altered membrane character/integrity in response to CP treatment included upregulation of the genes encoding the *N*-succinyl-l-diaminopimelic acid desuccinylase DapE (121), which is involved in cell wall peptidoglycan production, the competence lipoprotein ComL (122), and the twin-arginine translocation (Tat) and general secretion (Sec) systems (123), which transport proteins across the cytoplasmic membrane (Fig. S9). Furthermore, CP treatment resulted in the downregulation of genes for the oxidative stress-sensing regulator OspR (124) and the long-chain fatty acid responsive regulator PsrA (125) (Fig. S9).

### CP elicits transcriptional responses indicative of redirected chorismate flux in *P. aeruginosa* cocultured with *S. aureus*

**Fig 4:**
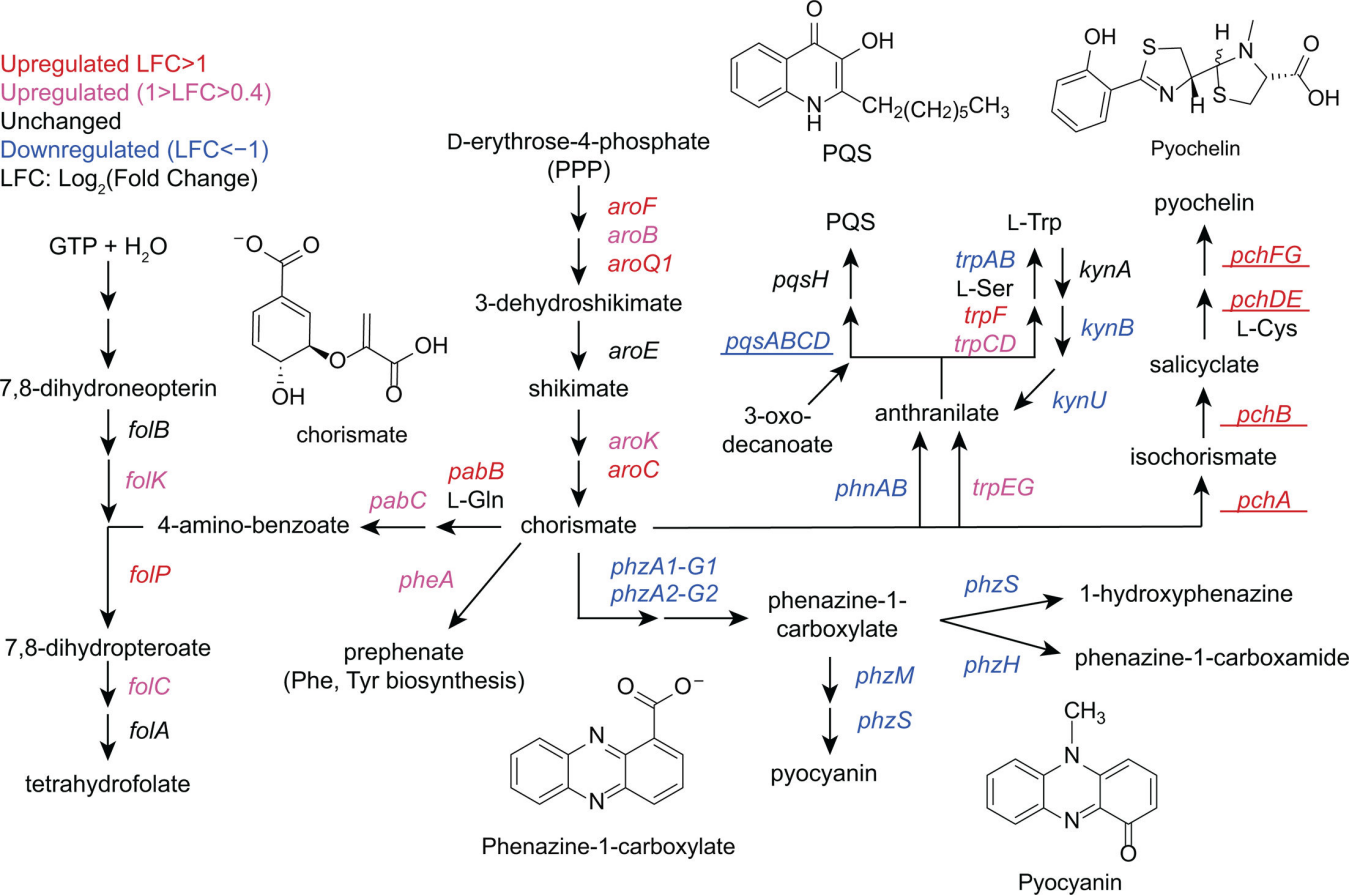
Gene expression analysis indicates that CP treatment redirects chorismate flux in *P. aeruginosa* cocultured with *S. aureus. P. aeruginosa* genes DE in response to CP treatment are shown. Genes that were DE to a similar extent in CP-treated and Fe-depleted cocultures are underlined.

 We also noticed a pattern among genes associated with the biosynthetic pathways utilizing chorismate, a key precursor for multiple secondary metabolites that contribute to the survival and virulence of *P. aeruginosa*. These secondary metabolites include the siderophore PCH (51, 126), the anthranilate-derived AQs (39), phenazines (127), the aromatic amino acid precursor prephenate (128), and 4-aminobenzoate (129), which is an intermediate for folate biosynthesis (130). DE analysis revealed that CP treatment, but not metal depletion, upregulated the expression of genes associated with the biosynthesis and utilization of 4-aminobenzoate and prephenate (Fig. 4; Table SF9). These changes indicated increased cellular requirements for folate and amino acids, which are needed to support metabolism. The expression of PCH biosynthetic machinery (131) was also upregulated as part of an Fe-starvation response (see above) (66), indicating that some chorismate flux is directed toward PCH (Fig. 4). CP treatment also significantly decreased the expression of genes encoding phenazine biosynthetic machinery, which were among the most strongly downregulated genes identified (Fig. 4; Fig. S7). These observations are in agreement with prior metabolite analyses of *P. aeruginosa* in monoculture and in coculture with *S. aureus,* which showed that Fe depletion significantly decreased phenazine levels, and CP treatment resulted in near-complete suppression of phenazine production (57, 66). Moreover, the expression of genes encoding the PhnAB anthranilate synthase (132–135) and AQ biosynthetic machinery (*pqs* operon) (41, 136) were downregulated in response to CP treatment; we observed that Fe limitation affected the expression of these genes to a lesser extent than CP treatment (Fig. 3 and 4). Overall, our analysis revealed that CP treatment elicits transcriptional responses indicative of redirected chorismate flux in *P. aeruginosa* in coculture with *S. aureus* and in monoculture (Table SF9). Our results also indicate that the transcriptional responses of *P. aeruginosa* to CP treatment are complex and involve both metal-dependent and metal-independent effects (66).

### CP treatment perturbs autoinducer production and dampens QS in *P. aeruginosa* cocultured with *S. aureus*

**Fig 5:**
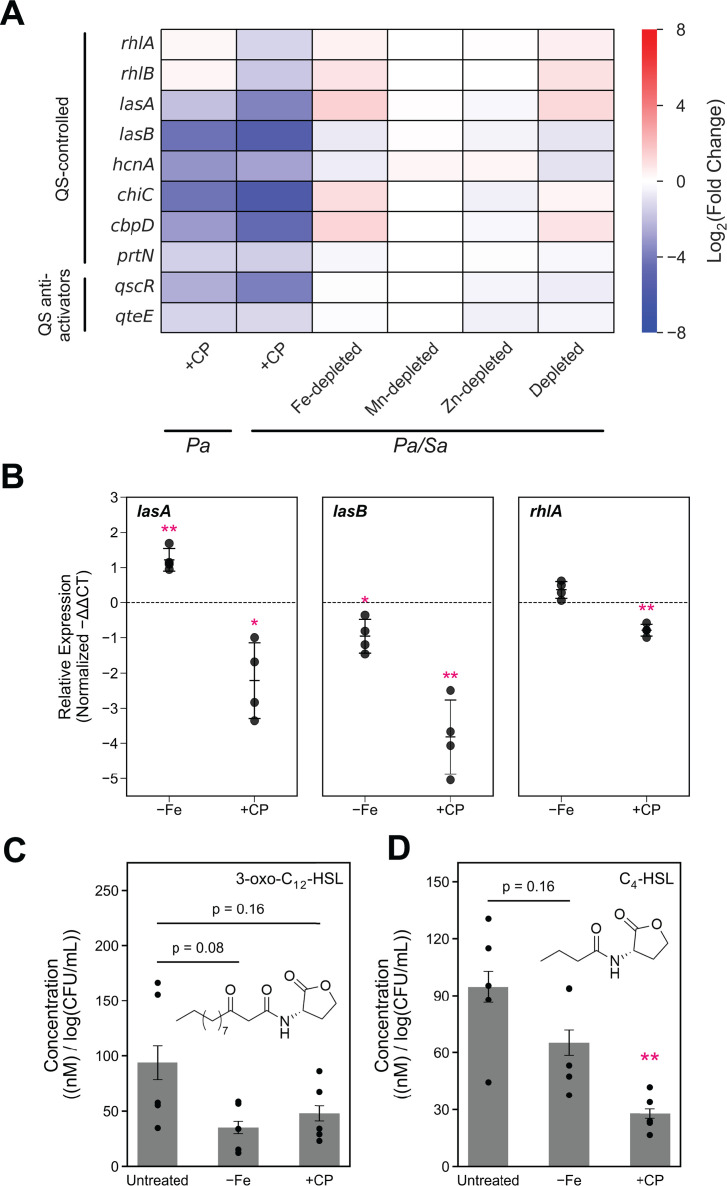
CP downregulates the expression of genes controlled by QS and decreases the production of autoinducers in *P. aeruginosa* cocultured with *S. aureus*. (**A**) DE heatmap of QS-controlled genes identified by RNA-seq. *Pa* indicates *P. aeruginosa* monoculture, and *Pa/Sa* indicates the coculture. (**B**) DE of selected QS-controlled genes quantified by real-time PCR. (**C and D**) CP treatment and Fe depletion decrease the production of the QS autoinducers 3-oxo-C_12_-HSL (**C**) and C_4_-HSL (**D**) in *P. aeruginosa* cocultured with *S. aureus*. Cultures were grown in metal-replete CDM ± 20 µM CP or Fe-depleted CDM and incubated at 37°C for 6 h. (**B**) Transcript levels were normalized to the *P. aeruginosa* housekeeping gene 16S, and the fold change after normalization is presented (*n* = 4, *P < 0.05 and **P < 0.01, error bars represent S.D.). (**C and D**) Aliquots of culture supernatants were collected and processed for quantitative mass spectrometry. Metabolite levels were normalized to *P. aeruginosa* CFUs (*n* = 5, error bars represent S.E.). For comparison with the untreated culture condition, **P < 0.01.

DE analysis indicated that CP treatment resulted in the downregulation of *lasA*, *lasB*, and genes encoding rhamnolipid production (*rhlAB*) (137) (Fig. 5A), which are known to be controlled by QS. These trends were not observed in Fe-depleted or metal-depleted cocultures. To further interrogate these findings, we examined how CP treatment and Fe depletion affected the expression of these QS-controlled genes using real-time PCR. Consistent with trends revealed by RNA-seq, CP treatment decreased the expression of *lasA*, *lasB*, and *rhlA* in *P. aeruginosa* cocultured with *S. aureus* (Fig. 5B). While Fe depletion slightly decreased the expression of *lasB*, this condition increased the expression of *lasA* and did not change the expression of *rhlA* (Fig. 5B). These results indicate that CP treatment attenuates the expression of QS-controlled genes in *P. aeruginosa* cocultured with *S. aureus* in a manner that is largely independent of Fe depletion.

Based on these findings, we suspected that CP may be acting on the LasIR and RhlIR QS systems, which govern the regulation of LasA and LasB (21, 23, 138, 139). However, our efforts to understand the impact of CP on the expression of both QS systems were complicated by expression trends indicating overlap between the effects of CP treatment and Fe depletion. To further probe the effects of CP treatment on QS in *P. aeruginosa*, we utilized triple-quadrupole mass spectrometry to quantify the levels of the autoinducers 3-oxo-C_12_-HSL and C_4_-HSL in coculture supernatants as a readout of QS molecules in *P. aeruginosa* (140, 141). Because CP treatment has negligible effects on the growth kinetics of *P. aeruginosa* cocultured with *S. aureus* (66), differences in levels of homoserine lactones (HSLs) are unlikely to be a result of differences in growth phase (142). Our mass spectrometric analysis revealed that CP treatment and Fe depletion slightly decreased levels of 3-oxo-C_12_-HSL, but the change was not statistically significant (Fig. 5C). By contrast, CP treatment decreased the levels of C_4_-HSL by approximately 70% at the 6-h time point (Fig. 5D). Fe depletion caused no significant change in C_4_-HSL levels at the 6-h time point (Fig. 5D). At the 11-h time point, CP treatment did not significantly alter the levels of 3-oxo-C_12_-HSL (Fig. S10A), indicating that production of 3-oxo-C_12_-HSL in CP-treated cocultures recovered to levels found in metal-replete cultures between 6 and 11 h. By contrast, the levels of C_4_-HSL were decreased by both CP treatment and Fe depletion at 11 h (Fig. S10B), indicating that the effect of CP on C_4_-HSL production occurs early in the culture time course and persists through the 11-h time point. It is difficult to conclude from these data whether the effect of CP on C_4_-HSL levels can be attributed to Fe depletion.

To further understand how perturbed production of 3-oxo-C_12_-HSL and C_4_-HSL affected transcriptional responses of *P. aeruginosa*, we examined the expression of other *P. aeruginosa* genes that are known to be QS-controlled. Consistent with attenuated QS for *P. aeruginosa* in CP-treated cocultures, strong downregulation of genes encoding QS-controlled systems was also observed under these culture conditions. These genes encode phenazine biosynthetic machinery (*phz*), hydrogen cyanide production (*hcn*) (143), chitin-binding protein (*cbpD*) (144), and PA-I galactophilic lectin (*lecA*) (145, 146) (Fig. 5A; Fig. S7). Furthermore, substantial overlap was found between genes that were DE in response to CP treatment and genes previously identified to be QS-controlled by microarray analysis (23, 147). Out of 311 genes known to be positively regulated by QS (23), 245 (78.8%) were downregulated in CP-treated cocultures, of which 75 (24.1%) overlapped with the effects of Fe or Zn depletion, and 170 (54.7%) were attributable only to the effects of CP treatment. We also observed that CP treatment resulted in downregulated expression of *qscR* (*PA14_39980*) (148, 149) and *qteE* (*PA14_30560*) (150), genes encoding recently identified QS anti-activator proteins (Fig. 5A), further suggesting perturbed QS. In addition, the QS-regulated *prtN* gene was downregulated in response to CP treatment (151) (Fig. 5A). Collectively, these findings suggest that CP perturbs the production of *P. aeruginosa* AHL autoinducers primarily through effects on C_4_-HSL (Fig. 5D), resulting in attenuated QS by *P. aeruginosa* in coculture. This noteworthy effect of CP on autoinducer production likely impacts the expression of multiple virulence factors that directly impact coculture dynamics between *P. aeruginosa* and *S. aureus*.

Interactions between bacterial pathogens and host factors can lead to perturbations in bacterial QS and virulence. Prior studies have found that binding of 3-oxo-C_12_-HSL and its degradation product by the host protein albumin inhibited QS in *P. aeruginosa* and decreased the anti-staphylococcal activity of *P. aeruginosa* cocultured with *S. aureus* (152). Mammalian paraoxonases were previously shown to possess lactonase activity against 3-oxo-C_12_-HSL, and transgenic expression of the paraoxonase PON1 in a *Drosophila melanogaster* model was shown to confer protection against *P. aeruginosa* infection (153–156). Host-derived metabolites have also been reported to modulate bacterial QS; ethanolamine was identified as a host metabolite that perturbs QS in *Vibrio cholerae*, and human Caco-2 cells were found to produce a mimic of the QS signal molecule autoinducer-2, thus activating QS-controlled responses in *Salmonella enterica* serovar Typhimurium (157, 158). Our findings contribute to a growing body of evidence indicating that the host environment impacts bacterial QS and highlight the importance of considering such effects in studies of the host–pathogen interface.

### CP treatment decreases the production of antimicrobials by *P. aeruginosa* cocultured with *S. aureus*

We recently showed that CP treatment and Fe depletion have opposing effects on *S. aureus* viability during coculture with *P. aeruginosa* (66). Previous studies have independently shown that CP treatment and Fe starvation have opposing effects on AQ production (55, 64). Considering that these studies were done under different experimental conditions, we were motivated to evaluate the effects of CP treatment and Fe levels on AQ production by *P. aeruginosa* in parallel. *P. aeruginosa* produces multiple AQs such as 2-heptyl-4(1*H*)-quinolone (HHQ) (39–41, 56), 2-heptyl-3-hydroxy-4(1*H*)-quinolone (PQS) (19, 39), and the potent anti-staphylococcal metabolite 2-heptyl-4-hydroxyquinoline N-oxide (HQNO) (42). PQS and its precursor HHQ function as QS molecules that modulate the activation of the virulence regulator MvfR (PqsR) and its regulon (39, 40, 159). PQS has been identified as a key QS signal molecule that controls many aspects of *P. aeruginosa* virulence (18, 160), including the expression of *lasB*. RNA-seq revealed that CP treatment decreased the expression of genes associated with AQ production (Fig. 3 and 4). To further investigate these trends, we performed real-time PCR studies to examine the expression of AQ biosynthetic machinery in response to CP treatment and Fe depletion (41, 136). We observed that CP treatment slightly downregulated the expression of *pqsD* (Fig. S11A) and decreased the expression of *pqsE* (Fig. S11B), while Fe depletion resulted in significant downregulation of *pqsD* and *pqsE* (Fig. S11), indicating that the effect of CP on *pqsD* and *pqsE* is attributable to Fe(II) sequestration by CP. Furthermore, we suspected that anthranilate production by *P. aeruginosa* may be reduced in the presence of CP due to the downregulation of *phnAB* and *kynBU* (Fig. 4), which encode the only two experimentally verified sources of anthranilate for AQ production (41, 133, 161–163).

**Fig 6:**
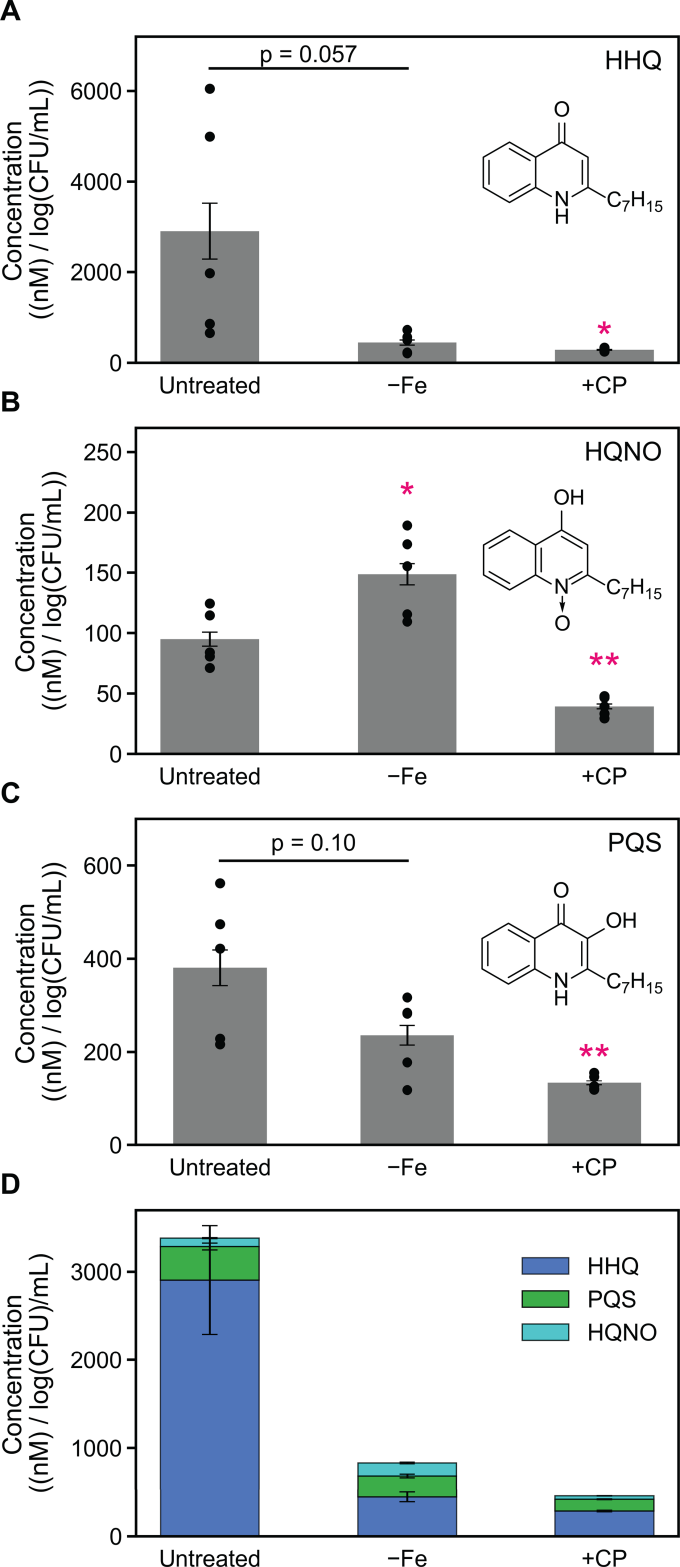
The presence of CP decreases the production of C_7_ AQs in *P. aeruginosa*/*S. aureus* cocultures. CP treatment decreases the levels of HHQ (**A**), HQNO (**B**), PQS (**C**), and the overall C_7_ AQ pool (**D**). Aliquots of culture supernatants were collected from cocultures of *P. aeruginosa* and *S. aureus* grown in Fe-depleted CDM or metal-replete CDM ± 20 µM CP at 37°C for 11 h and processed for quantitative mass spectrometry. Metabolite levels were normalized to *P. aeruginosa* CFUs (*n* = 5, error bars represent S.E.). For comparison with the untreated culture condition, *P < 0.05 and **P < 0.01.

Based on transcriptional responses suggesting decreased AQ production in response to CP treatment, we used mass spectrometry to ascertain whether CP treatment decreased the production of AQs by *P. aeruginosa* cocultured with *S. aureus*. The alkyl chains in AQs can vary in length and saturation, with the C_7_ congeners being the first studied (19, 164, 165). Because *P. aeruginosa* also synthesizes C_9_ AQ congeners (40) that possess anti-staphylococcal activity similar to the C_7_ congeners (56), we included the C_9_ AQ congeners 2-nonyl-4(1*H*)-quinolone (NHQ), 2-nonyl-4-hydroxyquinoline N-oxide (NQNO), and 2-nonyl-3-hydroxy-4(1*H*)-quinolone (C_9_-PQS) in our analysis. Using triple-quadrupole mass spectrometry, we quantified AQ levels from the same coculture supernatants used for the analysis of HSLs. Strikingly, CP treatment considerably decreased levels of HHQ, HQNO, and PQS at the 6-h (Fig. S12A through C) and 11-h (Fig. 6A through C) time points. While Fe depletion also decreased levels of HHQ at both time points, levels of HQNO were increased by Fe depletion at both time points (Fig. S12A and B; Fig. 6A and B), although we note that in some cases the difference was not statistically significant. PQS levels in Fe-depleted cocultures were slightly increased at 6 h (Fig. S12C) and decreased at 11 h (Fig. 6C), suggesting that production of PQS in cocultures grown in Fe-depleted CDM may be negatively autoregulated at this later time point during growth. CP treatment and Fe depletion decreased the overall pool of C_7_-AQs at both time points (Fig. 6D; Fig. S13), which was primarily driven by decreased levels of HHQ.

**Fig 7:**
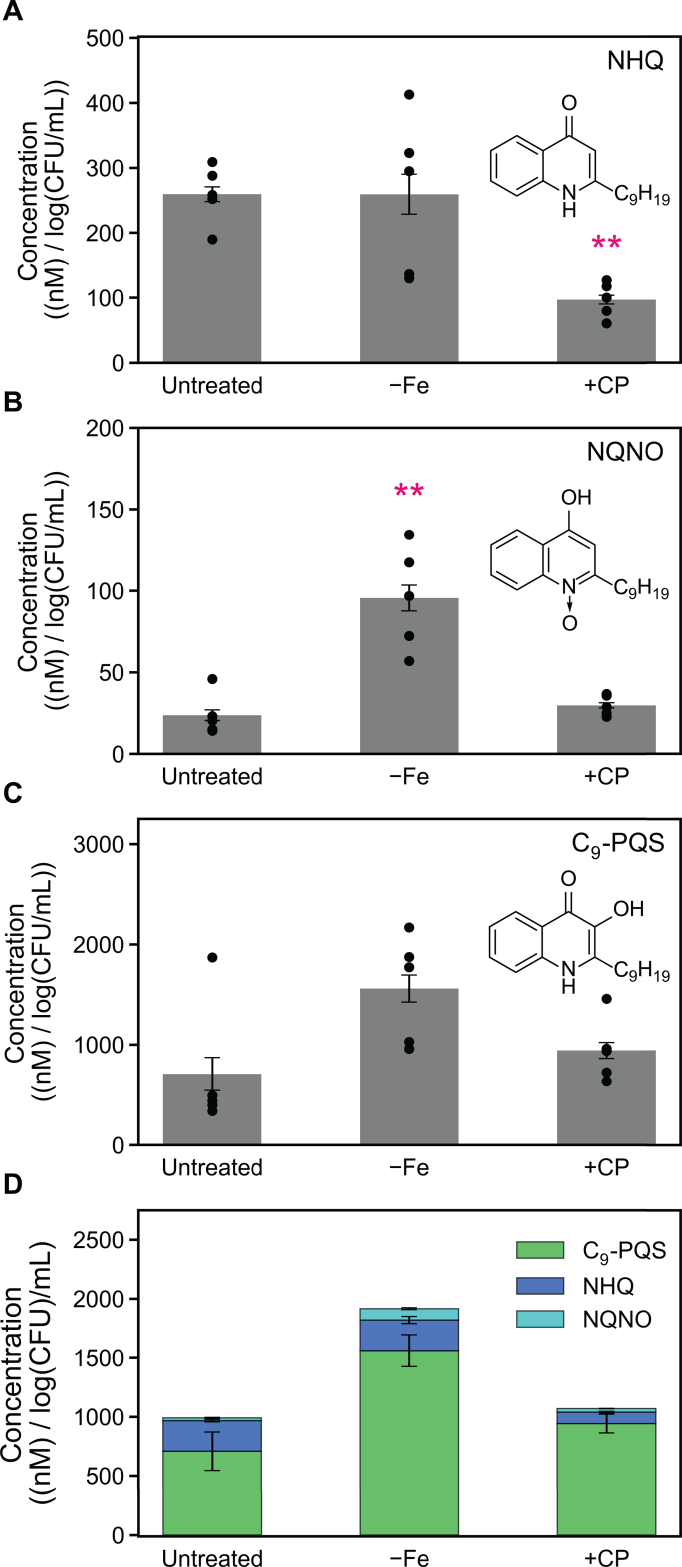
The presence of CP decreases levels of the C_9_ AQ NHQ in *P. aeruginosa*/*S. aureus* cocultures. CP treatment decreased the levels of NHQ (**A**) but did not significantly affect levels of NQNO (**B**), C_9_-PQS (**C**), or the overall C_9_ AQ pool (**D**). Aliquots of culture supernatants were collected from cocultures of *P. aeruginosa* and *S. aureus* grown in Fe-depleted CDM or metal-replete CDM ±20 µM CP at 37°C for 11 h and processed for quantitative mass spectrometry. Metabolite levels were normalized to *P. aeruginosa* CFUs (*n* = 5, error bars represent S.E.). For comparison with the untreated culture condition, **P < 0.01.

Consistent with the C_7_ congeners, CP treatment resulted in a marked decrease in NHQ levels at both the 6-h (Fig. S14A) and 11-h (Fig. 7A) time points, a decrease that was not observed for Fe-depleted cocultures. By contrast, NQNO levels were significantly increased in Fe-depleted cocultures and unchanged in CP-treated cocultures at both time points (Fig. S14B; Fig. 7B). Levels of C_9_-PQS were increased in CP-treated cocultures and Fe-depleted cocultures at the 6-h time point (Fig. S14C) but were not significantly altered at 11 h (Fig. 7C), in agreement with trends observed for C_7_-PQS (Fig. S12C; Fig. 6C). Changes in the overall pool of C_9_ AQs were primarily driven by changes in the levels of C_9_-PQS (Fig. S15; Fig. 7D). Together, our findings show that the effect of CP on the C_9_ AQs occurs primarily on NHQ, which exhibits significant anti-staphylococcal activity in Fe-limited conditions (56) such as those expected in the presence of CP.

 Overall, our analysis of AQ production by *P. aeruginosa* in coculture is consistent with transcriptional responses indicating redirected chorismate flux, with decreased chorismate flux toward phenazine and AQ production (Fig. 4). Furthermore, our results are in agreement with prior observations of the opposing effects of CP treatment and Fe depletion on *S. aureus* survival in coculture with *P. aeruginosa* (55, 56, 64, 66). Collectively, our findings indicate that one aspect of CP-mediated *S. aureus* survival in coculture with *P. aeruginosa* (64, 66) involves decreased production of the C_7_ AQs HHQ, HQNO, and PQS, as well as the C_9_ AQ NHQ. The observation that CP treatment decreased PQS production was also consistent with CP-induced downregulation of *psrA* (166) (Fig. S9), encoding a protein known to positively regulate PQS production. By contrast, *P. aeruginosa* produced increased levels of the anti-staphylococcal metabolites HQNO and NQNO in Fe-depleted cocultures, which contribute toward heightened anti-staphylococcal activity by *P. aeruginosa* in Fe-limited environments.

### *S. aureus* cocultured with *P. aeruginosa* mounts Fe-starvation responses in the presence of CP but not in Fe-depleted conditions

**Fig 8:**
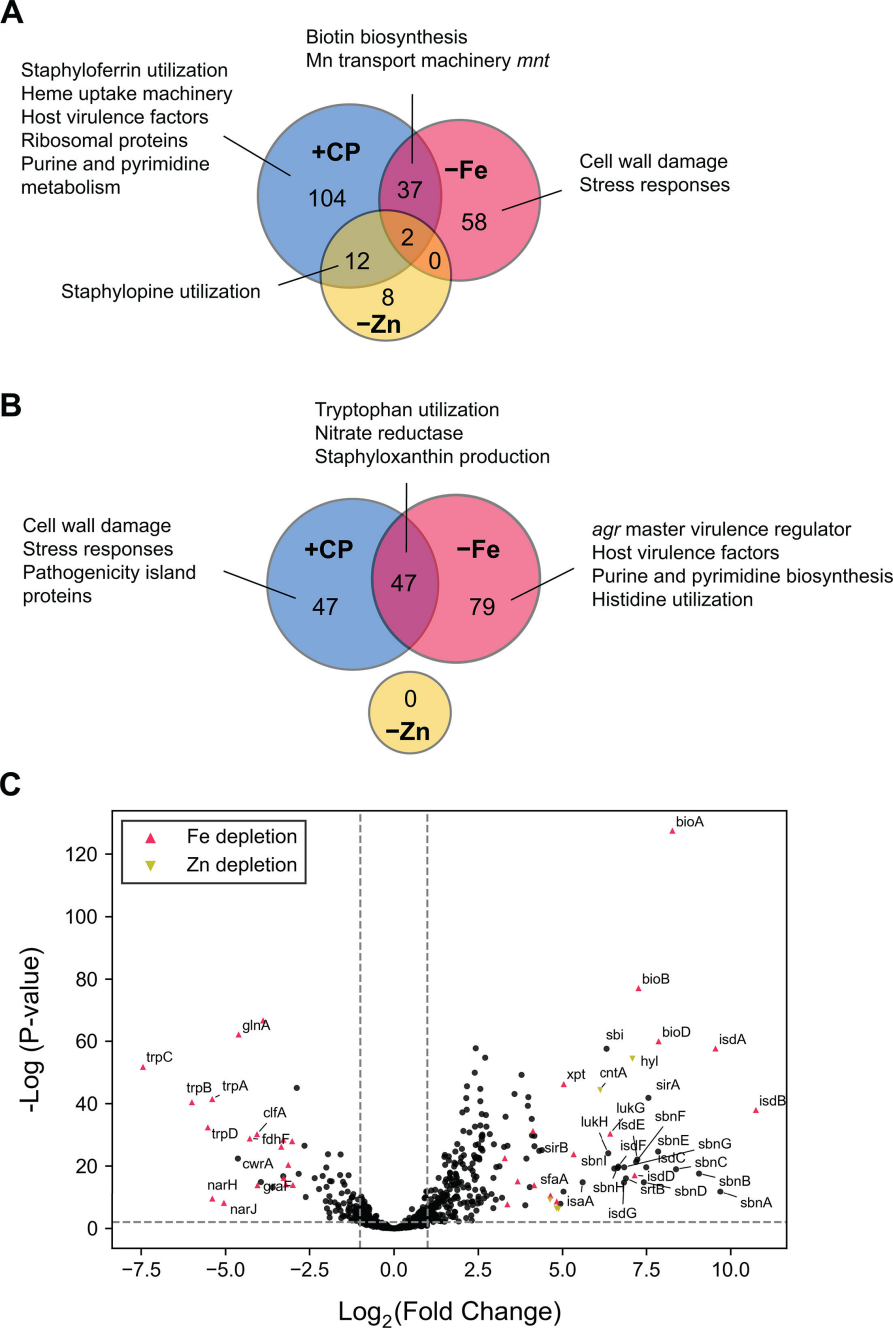
DE profiles of CP treatment and Fe depletion overlap in *S. aureus* cocultured with *P. aeruginosa*. Venn diagrams of the top 600 DE *S. aureus* genes across all conditions tested reveal partial overlap for upregulated (**A**) genes and considerable overlap of downregulated (**B**) genes in CP-treated cocultures and cocultures grown in Fe-depleted CDM. (**C**) Volcano plot of DE changes in response to CP treatment. Genes with similar DE patterns in response to Fe depletion or Zn depletion are denoted as colored shapes. A threshold cutoff log_2_(fold change) of 1 was employed. The complete list of DE *S. aureus* genes identified in each condition is presented in Tables SF11 to SF17.

The above analysis highlights the distinct impacts of CP treatment and Fe limitation on *P. aeruginosa* AQ production, with likely consequences on *S. aureus* viability in coculture. To gain an improved understanding of how *S. aureus* responds to CP treatment and metal limitation, analysis of the *S. aureus* transcriptome during coculture was performed. In response to CP, *S. aureus* upregulated multiple systems associated with metal acquisition and siderophore utilization (Fig. 8A and C), indicating that CP elicits multi-metal starvation responses from *S. aureus* in coculture. Our findings are consistent with prior studies examining the transcriptional responses of *S. aureus* to CP treatment, which include real-time PCR studies of *S. aureus* cocultured with *P. aeruginosa* (66) and a recent RNA-seq study of *S. aureus* monocultures (60). However, Venn analysis of the top 300 DE genes across all conditions revealed only partial overlap between the transcriptional responses of *S. aureus* in coculture to CP treatment and metal depletion (Fig. 8A and B), prompting further analysis of these distinct effects (Fig. S16 to S20).

**Fig 9:**
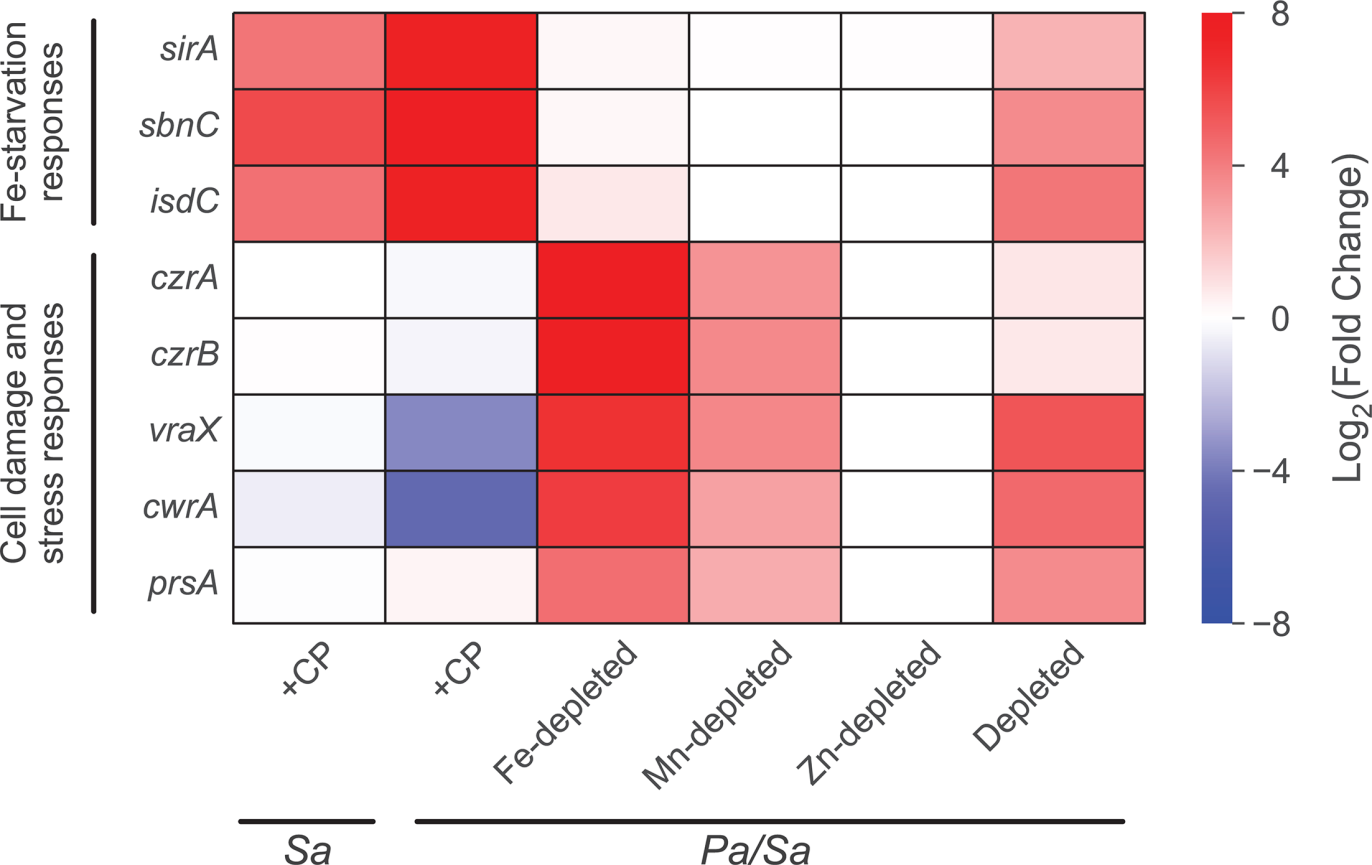
The presence of CP elicits Fe-starvation responses and decreases cell damage and stress responses by *S. aureus* cocultured with *P. aeruginosa*. DE heatmap of *S. aureus* genes associated with Fe-starvation responses as well as cell damage and stress responses. *Sa* indicates *S. aureus* monoculture, and *Pa/Sa* indicates the coculture.

In agreement with prior real-time PCR studies, CP treatment elicited robust Fe-starvation responses from *S. aureus* in monoculture (59, 60) and in coculture with *P. aeruginosa* (66). These responses include the upregulation of genes encoding heme uptake machinery (*isd*) (54) and staphyloferrin biosynthesis and transport (*sir* and *sbn*) (167, 168). Unexpectedly, the upregulation of these hallmark Fe-starvation responses was not observed for *S. aureus* in Fe-depleted cocultures and was attenuated for *S. aureus* in metal-depleted cocultures (Fig. 9). These surprising results suggest that the ability of *S. aureus* to mount Fe-starvation responses is inhibited by the anti-staphylococcal activity of *P. aeruginosa*, which is exacerbated by Fe depletion and mitigated by CP treatment (55, 56, 64, 66).

In agreement with this notion, a cluster of genes was found to be upregulated in Fe-depleted cocultures but not in CP-treated cocultures. These genes encode the Zn-responsive transcriptional repressor and efflux transporter CzrAB (169, 170), the cell wall inhibition-responsive protein CwrA (171)*,* the peptidylprolyl isomerase PrsA (172, 173), and superoxide stress responses (Fig. 9; Fig. S21), reflecting increased levels of cell wall stress and damage for *S. aureus* in Fe-depleted cocultures. Transcriptional responses of *S. aureus* in metal-depleted cocultures indicated decreased severity of cell wall damage and stress compared to Fe-depleted cocultures (Fig. 9; Fig. S22) despite similar impacts on *S. aureus* viability under both culture conditions (66). CP treatment also resulted in transcriptional responses indicating increased translational activity, evident from the upregulation of genes associated with translational machinery and metabolism, and downregulation of the genes encoding the transcriptional repressor LexA (174, 175) and the ribosome-associated inhibitor protein RaiA (176) (Fig. S22). By contrast, genes associated with translational activity were downregulated in *S. aureus* under Fe-depleted cocultures; this downregulation was attenuated in *S. aureus* under metal-depleted cocultures (Fig. S22).

These data point to a model in which CP promotes the survival of *S. aureus* in coculture with *P. aeruginosa* by reducing the anti-staphylococcal activity of *P. aeruginosa*, as evident from transcriptional responses indicating perturbed QS and decreased AQ production in *P. aeruginosa*, and decreased cell wall damage and increased metabolism in *S. aureus*. As a result, *S. aureus* cocultured with *P. aeruginosa* in the presence of CP is able to mount Fe-starvation responses. We speculate that the ability of *S. aureus* to mount Fe-starvation responses (although attenuated) in metal-depleted cocultures but not in Fe-depleted cocultures may stem from the effects of multi-metal depletion (Supplemental Discussion).

### CP treatment increases the expression of genes associated with host virulence in *S. aureus* cocultured with *P. aeruginosa*

**Fig 10:**
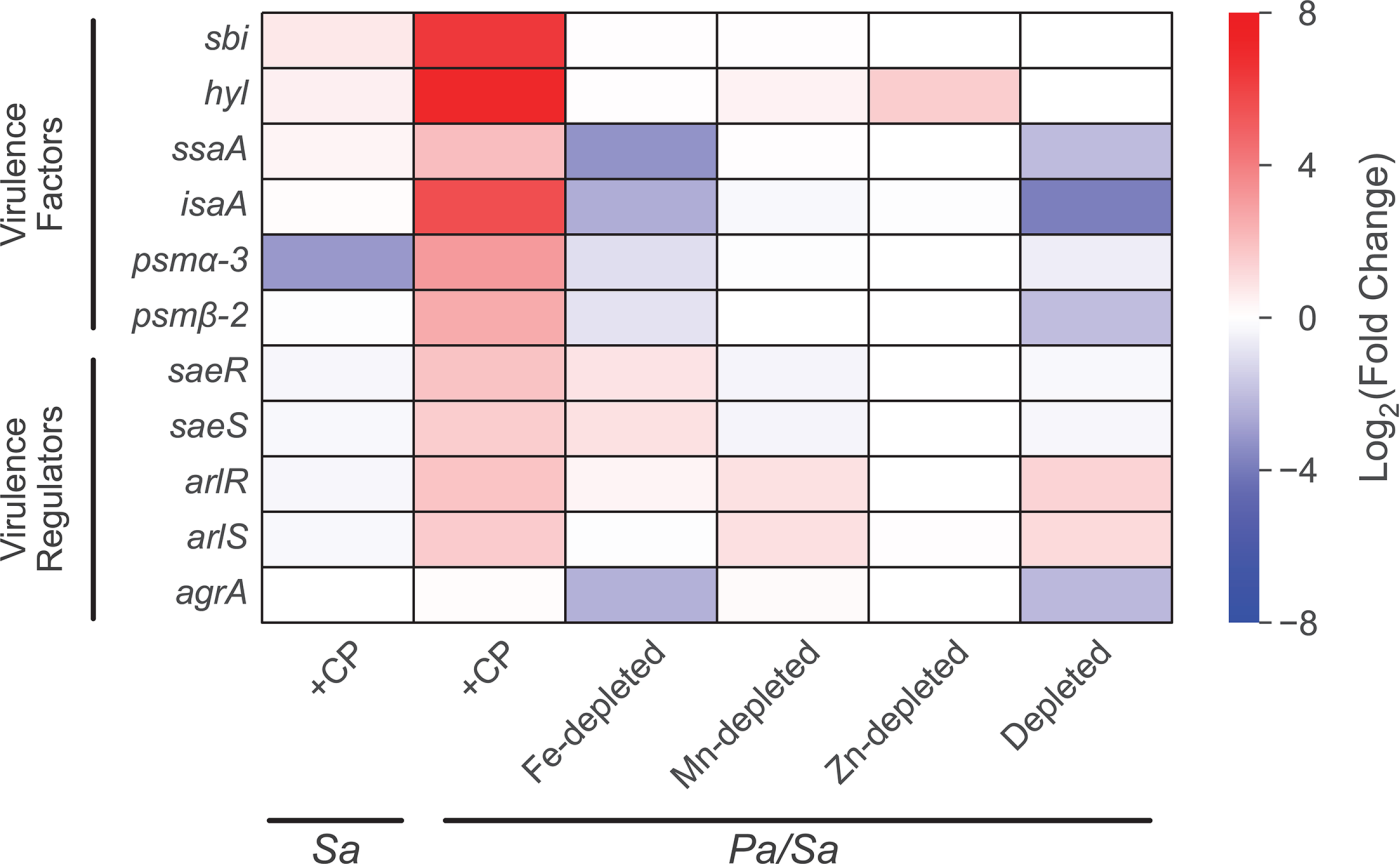
CP treatment upregulates the expression of virulence factors and regulators in *S. aureus* cocultured with *P. aeruginosa*. DE heatmap of *S. aureus* genes associated with host virulence factors and virulence regulation. *Sa* indicates *S. aureus* monoculture, and *Pa/Sa* indicates the coculture.

CP treatment upregulated the expression of multiple *S. aureus* systems associated with host virulence in coculture, in agreement with a prior RNA-seq study examining the effect of CP on *S. aureus* Newman in monoculture (60). We observed that CP treatment uniquely upregulated the expression of genes encoding the immunoglobulin-binding protein Sbi (177), alpha-hemolysin (*hyl*) (178), lytic transglycosylase IsaA (179), the secretory antigen SsaA (180), and the phenol-soluble modulins (*psm*) (181) in coculture (Fig. 10; Table SF19). These findings were consistent with CP-induced upregulation of genes for the SaeRS two-component system (182–184), which regulates many *S. aureus* host virulence factors (Fig. 10). The expression of *agr* encoding the master virulence regulator (185) remained unchanged in response to CP treatment and was decreased in response to Fe depletion (Tables SF15B and SF20). In addition, the expression of the *S. aureus* QS effector RNAIII was decreased in response to CP treatment, Fe depletion, and Mn depletion (Fig. S23). Together, these findings indicate that CP-mediated upregulation of *S. aureus* host virulence genes is unlikely to be due to Agr-mediated regulation or metal sequestration by CP. The upregulation of *psmα-3* was consistent with increased expression of the sRNA Teg41 (*srna_1080_RsaX05*) (Fig. S23; Table SF21), which was previously shown to be important for virulence in *S. aureus* and required for the expression of the alpha phenol-soluble modulins (186, 187). Intriguingly, CP treatment increased the expression of the sRNA RsaA for *S. aureus* in coculture (Fig. S23), which is known to suppress the translation of the pleiotropic virulence regulator MgrA (188, 189). We note that the effect of CP on *S. aureus* monocultures was attenuated (Fig. 10), which most likely stems from variations in the growth phase of *S. aureus* and culture conditions between this work and previous investigations (60, 66).

We also observed that CP treatment in coculture resulted in upregulated expression of the *S. aureus dltABCD* operon (60, 190), which is responsible for the modification of cell wall teichoic acids in response to environmental stresses (Fig. S24). The presence of CP also led to upregulation of genes for the KdpDE two-component system (191), which is involved in virulence regulation by sensing extracellular K^+^ levels. In addition, CP treatment downregulated the expression of genes encoding Clp protease, which is involved in protein homeostasis and the expression of various virulence factors in *S. aureus* (60, 192). Finally, we observed that the presence of CP slightly perturbed the expression of genes encoding two global regulators, *sigB* (193, 194) and *sarA* (195–197), which are involved in *S. aureus* virulence and adaptation (Fig. S24). We did not observe comparable upregulation of *S. aureus* host virulence genes in monocultures treated with CP. Overall, the increased expression of host virulence genes in the presence of CP is consistent with the protective effect of CP on *S. aureus* cocultured with *P. aeruginosa* and highlights the profound impact of this protection on the *S. aureus* transcriptome.

### Working model and outlook

**Fig 11:**
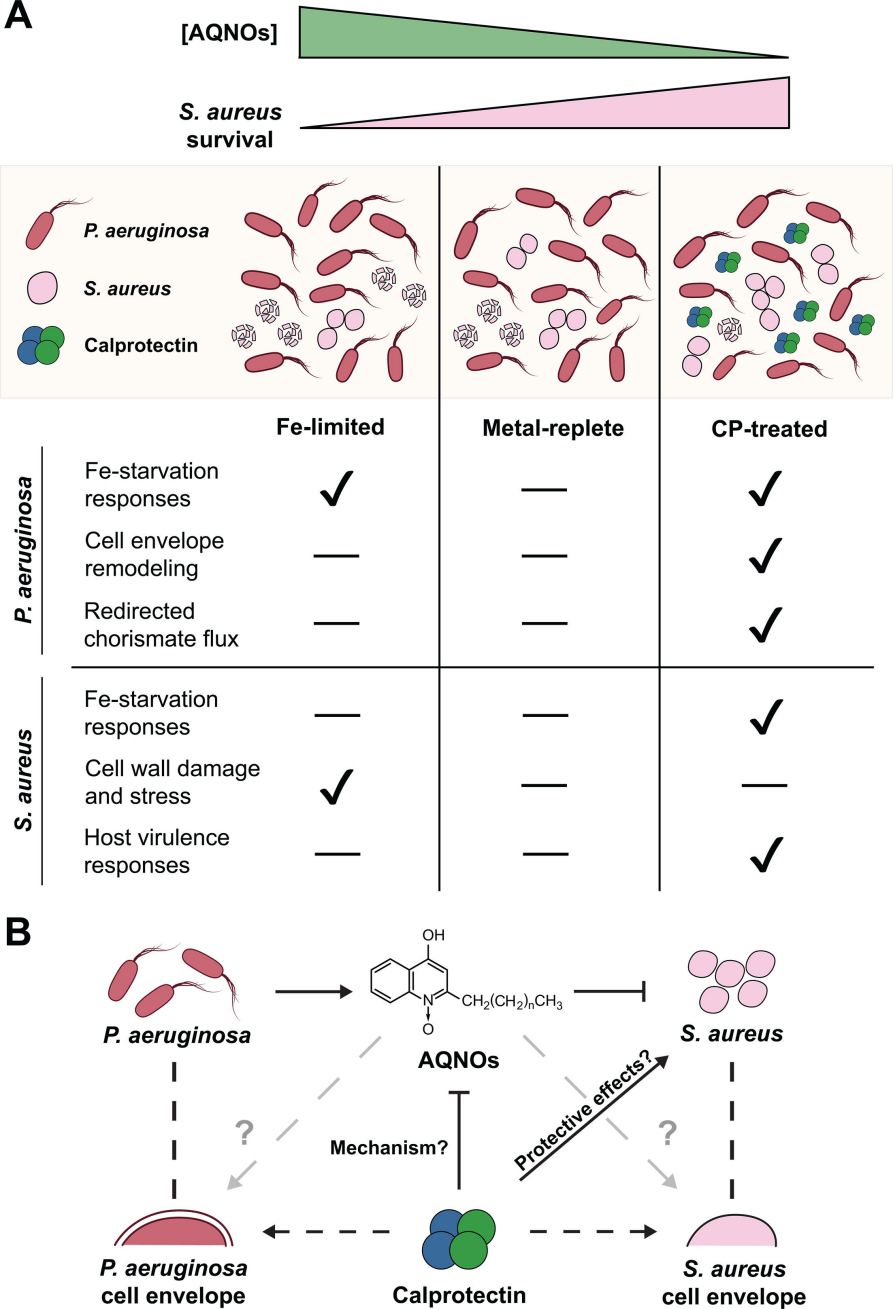
Current working model of the impact of CP on coculture dynamics between *P. aeruginosa* and *S. aureus*. (**A**) CP treatment and Fe depletion have distinct and opposing impacts on coculture dynamics. (**B**) Scheme of interactions between CP, AQNOs, and cocultures of *P. aeruginosa* and *S. aureus*.

The insights discussed here provide a working model for how CP and Fe availability impact coculture dynamics between *P. aeruginosa* and *S. aureus* (Fig. 11A). In this model, *P. aeruginosa* functions as an attacker that produces an arsenal of anti-staphylococcal factors, while *S. aureus* functions as a defender. Under metal-replete and metal-depleted conditions, *P. aeruginosa* attacks *S. aureus* via AQNOs and other secreted factors, and the inability of *S. aureus* to defend itself leads to decreased viability. CP effectively disarms *P. aeruginosa* by redirecting chorismate flux, perturbing autoinducer production, and decreasing the production of AQs. By decreasing the anti-staphylococcal activity of *P. aeruginosa*, the presence of CP increases the viability of *S. aureus* cocultured with *P. aeruginosa,* and thus *S. aureus* mounts Fe-starvation and host virulence responses that are otherwise not feasible in the presence of *P. aeruginosa*. Our findings highlight complexity in the transcriptional responses of both bacterial pathogens to CP, some of which overlap with responses to metal depletion and others appear to result from metal-independent effects of the protein.

This study and the resulting model present several outstanding questions that warrant future investigation (Fig. 11B). First, this work motivates investigating the mechanism by which AQ levels are modulated by CP treatment, i.e., does CP directly initiate a *P. aeruginosa* signaling cascade that results in decreased AQ production, or does *P. aeruginosa* reduce AQ production due to a distinct physiological response to CP? Second, the collective results from prior studies (58, 60, 66) and this work point to CP eliciting cell envelope changes in *P. aeruginosa* and *S. aureus*. Whether these membrane changes are linked to the observed decrease in AQ production remains to be investigated. Third, future work should examine whether CP-mediated protection of *S. aureus* results from CP boosting *S. aureus* defenses against *P. aeruginosa*—perhaps through cell envelope modifications—or if coculture dynamics are primarily driven by changes in AQ production. Fourth, given that CP-mediated protection of *S. aureus* occurs independently of metal sequestration, future studies should determine whether the CP protein scaffold, without its metal-binding sites, can recapitulate the transcriptional responses elicited by CP from cocultures of *P. aeruginosa* and *S. aureus* (66), or if any other structural features or functions of CP are required. Finally, we expect this model to pertain to the extracellular host environment where CP is released from the neutrophil and encounters these two bacterial pathogens, and the impact of these interactions on the host warrants exploration. These insights will likely be key to understanding the multifaceted activity of this remarkable protein and its consequences on interspecies interactions at the host–pathogen interface, such as in polymicrobial infections.

## MATERIALS AND METHODS

For complete materials and methods, please refer to the supplemental material.

### General experimental methods

General methods, including general microbiology methods and protein production and handling, were performed as previously reported 66.

### RNA extraction and workup for RNA-seq

RNA extraction and workup were carried out as previously reported (66). Following RNA precipitation and resuspension in nuclease-free water, samples were submitted to the MIT BioMicro Center for further preparation and sequencing. Sample integrity was validated using fluorescence-based electrophoresis (AATI Fragment Analyzer), and ribosomal depletion was performed using the NEB Next rRNA Depletion Kit (New England Biolabs). Subsequently, library preparation (adapter ligation, size selection, barcoding, and enrichment) was performed using the NEBNext Ultra II Directional RNA Library Prep Kit (New England Biolabs). The quality of the resulting libraries was validated using real-time PCR, following which the libraries were pooled and sequenced on a single lane of an Illumina NextSeq500 instrument using 75 nt chemistry.

### Bioinformatics workflow and analyses

Raw reads were aligned with the hisat2 aligner (198) using NCBI RefSeq reference genomes for *P. aeruginosa* PA14 (GCF_000014625.1) (199) and *S. aureus JE2* (GCF_002085525.1) (Walter Reed Army Institute of Research). The combined genome derived from the PA14 + JE2 genomes was used to align reads from coculture samples, and mapping fidelity was validated by verifying that no cross-mapping occurred. The aligned reads were quantified using Feature Aggregate Depth Utility (200). To check for sequencing depth and the presence of technical artifacts, rarefaction analysis and principal component analysis were performed. DE analysis of the quantified reads was performed using DESeq2 (version 1.40.2) (201). For both monocultures and cocultures, untreated cultures (in metal-replete CDM) served as the untreated control. Log2(fold changes) (LFCs) were calculated using the apeglm method for effect size shrinkage (202). Functional enrichment analyses were performed with clusterProfiler (203, 204) using the variance-stabilized LFCs for both species against the best available annotations for each species. To access these annotations, cross-species gene mapping was carried out with CD-HIT-EST-2D (205) to obtain unique gene mappings. For *P. aeruginosa*, overrepresentation analysis was performed using curated PAO1 GO annotation available on the *Pseudomonas* genome database (pseudomonas.com) (206). For *S. aureus*, gene set enrichment analysis was performed using the NCBI RefSeq annotations available for *S. aureus* USA300 FPR3757 (CP000255.1) (207).

### Metabolite quantification by triple quadrupole mass spectrometry

HPLC-grade solvents were used for sample preparation and mass spectrometry. A 350 µL aliquot of culture suspension was centrifuged at 13,000 rpm for 5 min at 4°C to pellet cells and debris. A 300 µL aliquot of the supernatant was transferred to a new polypropylene tube, and 3 µL of 100 µM C_6_-HSL-d_3_ internal standard in methanol was added. The resulting mixture was extracted twice with an equal volume of acidified ethyl acetate containing 0.02% (vol/vol) acetic acid, each time by vigorous vortexing at 3,000 rpm at ambient temperature for 1 min. The upper organic layers were transferred to a clean glass vial, and the solvent was removed by rotary evaporation in a 35°C water bath. The resulting solid was resuspended in 900 µL of ice-cold methanol and transferred to a new ice-cold tube. The resuspended samples were centrifuged at 13,000 rpm for 10 min at 4°C to pellet any particulates, and 200 µL of the supernatant was transferred into a HPLC vial fitted with a polypropylene vial insert for analysis. Complete instrumentation data and HPLC conditions are provided in the supplemental material.

### Preparation of analyte standards

The homoserine lactones and alkylquinolone standards were obtained from commercial vendors and used as received. To determine the dynamic range of detection and obtain standard curves, analyte standards (2 nM–50 µM) were prepared by serial dilution of a fresh 1–10 mM stock solution of each analyte in methanol, with the exception of C_9_-PQS, which was instead dissolved into a 1:1 mixture of water:acetonitrile, each containing 0.1% formic acid. The analyte standards were centrifuged at 13,000 rpm for 10 min at 4°C to pellet any particulates and loaded into vials as described above.

## Data Availability

Raw reads obtained from RNA-seq were deposited in the NCBI Sequence Read Archive under the BioProject accession number PRJNA1295913. The complete lists of differentially expressed *P. aeruginosa* and *S. aureus* genes are available as accompanying supplemental material (Tables SF1 to SF21).

## SUPPLEMENTAL MATERIAL

Supplemental information msystems.00576-25-s0001.pdf Supplemental text, tables, figures, and references.

Tables SF1-SF10 msystems.00576-25-s0002.xlsx Gene expression data for *P. aeruginosa*.

Tables SF11-SF21 msystems.00576-25-s0003.xlsx Gene expression data for *S. aureus*.

ASM does not own the copyrights to Supplemental Material that may be linked to, or accessed through, an article. The authors have granted ASM a non-exclusive, world-wide license to publish the Supplemental Material files. Please contact the corresponding author directly for reuse.
