# Supplementary material for: Calprotectin protects Staphylococcus aureus in coculture with Pseudomonas aeruginosa by attenuating quorum sensing and decreasing the production of pseudomonal antimicrobials
Source: mSystems. 2025 Sep 4;10(9):e00576-25. doi: 10.1128/msystems.00576-25 (PMC12455946; doi:10.1128/msystems.00576-25)
Supplement: Supplemental information — Supplemental text, tables, figures, and references. [file msystems.00576-25-s0001.pdf]

Supplementary Information for

Calprotectin protects *Staphylococcus aureus* in coculture with *Pseudomonas aeruginosa* by attenuating quorum sensing and decreasing the production of pseudomonal antimicrobials

Wei H. Lee[1], Amanda G. Oglesby[2,3,#], and Elizabeth M. Nolan[1,#]

[1]Department of Chemistry, Massachusetts Institute of Technology, Cambridge, MA 02139, United States.

[2]Department of Pharmaceutical Sciences, School of Pharmacy, University of Maryland, Baltimore, MD 21201, United States.

[3]Department of Microbiology and Immunology, School of Medicine, University of Maryland, Baltimore, MD 21201, United States.

#Address correspondence to: Elizabeth M. Nolan, lnolan@mit.edu and Amanda. G. Oglesby, aoglesby@rx.umaryland.edu

This PDF file includes:

Table of Contents
Experimental Materials and Methods
Supplementary Discussion
Supplementary Tables S1-S7
Supplementary Figures S1-S36
Supplementary References

TABLE OF CONTENTS

Supplementary Experimental

General Materials and Methods.. S5
Mass Spectrometry.. S5
Microbiology.. S6
Bioinformatics Workflow and Analyses…………………………………………………………… S7

Supplementary Discussion

Distinct transcriptional responses of *P. aeruginosa* to CP treatment and metal depletion............ S9
Distinct transcriptional responses of *S. aureus* to CP treatment and metal depletion................... S12
Zn availability contributes to the metabolism of *S. aureus* cocultured with *P. aeruginosa* when part of a multi-metal starvation response... S16

Supplementary Tables

Table S1. Detection of transcripts for the *S. aureus* housekeeping gene *sigA*.............................. S17
Table S2. The proportion of differentially expressed *P. aeruginosa* and *S. aureus* genes across conditions.. S17
Table S3. Key statistics and average size of *P. aeruginosa* and *S. aureus* libraries obtained from RNAseq.. S18
Table S4. Optimized collision and fragmentation parameters for the selected analytes............... S19
Table S5. Primers used for real-time PCR of *P. aeruginosa*... S19
Table S6. Standard curves for real-time PCR of *P. aeruginosa* genes associated with quorum-sensing, alkylquinolone production and translational activity……………………………………… S20
Table S7. Effective dynamic range and standard curve fitting of analytes quantified by triple quadrupole mass spectrometry.. S20

Supplementary Figures

Figure S1. Transcriptional responses to Mn depletion overlap with responses to CP treatment in *P. aeruginosa* cocultured with *S. aureus* S21
Figure S2. Transcriptional responses to Fe depletion overlap significantly with responses to CP treatment in *P. aeruginosa* cocultured with *S. aureus*.. S21
Figure S3. Transcriptional responses to CP treatment and Zn depletion overlap significantly in *P. aeruginosa* cocultured with *S. aureus*………………………………………………………..... S22
Figure S4. Transcriptional responses of *P. aeruginosa* in coculture with *S. aureus* to CP resemble those seen for *P. aeruginosa* in monoculture ..……………………………………….. S22

Figure S5A. Overrepresentation analysis of upregulated *P. aeruginosa* genes across conditions reveals GO terms enriched across multiple conditions ……………………………………….. S23

Figure S5B. Overrepresentation analysis of upregulated *P. aeruginosa* genes across conditions S24

Figure S5C. Overrepresentation analysis of downregulated *P. aeruginosa* genes across conditions reveals GO terms enriched across multiple conditions ..……………………………. S25

Figure S5D. Overrepresentation analysis of downregulated *P. aeruginosa* genes across conditions ..…………………………………………………………………………… S26

Figure S6. Fe limitation impacts cytochrome preference and respiration in *P. aeruginosa* cocultured with *S. aureus*.. S27

Figure S7. CP decreases the expression of phenazine biosynthetic machinery in *P. aeruginosa* cocultured with *S. aureus*.. S27

Figure S8. CP elicits Zn-starvation responses for *P. aeruginosa* cocultured with *S. aureus*........ S28

Figure S9. Additional transcriptional responses of *P. aeruginosa* to CP treatment associated with cell envelope modifications.. S28

Figure S10. CP treatment and Fe depletion perturb production of the QS autoinducer C_4-HSL in *P. aeruginosa*/*S. aureus* co-cultures... S29

Figure S11. CP treatment and Fe depletion downregulate the expression of genes encoding for PQS biosynthetic machinery in *P. aeruginosa*/*S. aureus* cocultures.. S30

Figure S12. CP treatment decreases the production of C_7 alkylquinolones during early growth in *P. aeruginosa*/*S. aureus* co-cultures... S31

Figure S13. CP treatment and Fe depletion decrease overall levels of C_7 alkylquinolones during early growth in *P. aeruginosa*/*S. aureus* co-cultures.………...................……………….. S32

Figure S14. CP treatment decreases levels of the C_9 alkylquinolone NHQ and increases levels of C_9-PQS during early growth in *P. aeruginosa*/*S. aureus* co-cultures....................................... S33

Figure S15. Fe depletion and CP treatment increase overall levels of C_9 alkylquinolones during early growth in *P. aeruginosa*/*S. aureus* co-cultures.. S34

Figure S16. Transcriptional responses of *S. aureus* in coculture with *P. aeruginosa* to Mn depletion differ from responses to CP treatment... S34

Figure S17. Transcriptional responses of *S. aureus* in coculture with *P. aeruginosa* to Fe depletion overlap with responses to CP treatment.. S35

Figure S18. Transcriptional responses of *S. aureus* in coculture with *P. aeruginosa* to Zn depletion are distinct from responses to CP treatment.. S35

Figure S19. Transcriptional responses of *S. aureus* in coculture with *P. aeruginosa* to CP treatment differ significantly from responses of *S. aureus* in monoculture..........................…… S36

Figure S20. Gene set enrichment analysis of upregulated and downregulated *S. aureus* genes across conditions.. S36

Figure S21. Fe depletion upregulates superoxide stress responses, decreases methicillin resistance and increases the expression of urease genes in *S. aureus* cocultured with *P. aeruginosa*.. S37

Figure S22. CP treatment increases the expression of genes associated with translational activity in *S. aureus* cocultured with *P. aeruginosa*... S37

Figure S23. Differential expression heatmap of selected *S. aureus* regulatory RNAs………….. S38

Figure S24. Differential expression heatmap of other unique responses of *S. aureus* cocultured with *P. aeruginosa* to CP treatment ……………………………………………………………....... S38
Figure S25. CP treatment and Fe depletion increase the expression of *P. aeruginosa* genes involved in type III secretion systems and the production and transport of alkaline protease....... S39
Figure S26. CP treatment affects the expression of *P. aeruginosa* genes perturbed by the P443L mutation of the elongation factor mutant FusA1 in *P. aeruginosa* PAO1......................... S39
Figure S27. CP treatment affects the expression of translational machinery in *P. aeruginosa*..... S40
Figure S28. CP treatment increases translational activity and catabolite repression in *P. aeruginosa* cocultured with *S. aureus*... S40
Figure S29. Mn depletion increases the expression of genes involved in type VI secretion and alters nutrient preference in *P. aeruginosa* cocultured with *S. aureus*.. S41
Figure S30. Differential expression heatmap of other unique responses of *P. aeruginosa* monocultures to CP treatment.. S41
Figure S31. CP increases the expression of genes associated with Zn uptake in *S. aureus*.......... S42
Figure S32. CP elicits Mn starvation responses from *S. aureus* cocultured with *P. aeruginosa*... S42
Figure S33. Differential expression heatmap of other unique responses of *S. aureus* monocultures to CP treatment.. S43
Figure S34. Determination of effective range for quantification of C_6-HSL-d_3 using triple quadrupole mass spectrometry.. S44
Figure S35. Principal component analysis of *P. aeruginosa* libraries.. S45
Figure S36. Principal component analysis of *S. aureus* libraries... S45

Supplementary References.. S46

SUPPLEMENTARY EXPERIMENTAL

General Materials and Methods

Solutions, buffers and metal stocks. All chemicals and reagents were purchased commercially and used as received. All prepared solutions, buffers, and metal stocks were made using Milli-Q water (18.2 MΩ·cm, Milli-Q Academic system) and filtered (0.2 μm) before use. Trace metals basis reagents were used for all solutions, buffers, and metal stocks employed for microbiology. Metal stocks were prepared as previously reported (1).

Protein overexpression, purification and handling. CP (S100A8/S100A9 heterodimer) was overexpressed, purified and prepared for use in microbiology as previously reported (2). The concentration of protein was determined by absorbance measurement (ε_{280}: 18450 $M^{-1}cm^{-1}$).

Mass Spectrometry

General methods. For each analyte, the range of detection was determined using a standard curve of known concentrations. *N*-(3-Oxododecanoyl)-L-homoserine lactone (3-oxo-C_{12}-HSL), *N*-butanoyl-L-homoserine lactone (C_4-HSL), 2-heptyl-4-quinolone (HHQ) and 2-heptyl-3-hydroxy-4(*1H*)-quinolone (PQS) were obtained from Sigma. 2-Heptyl-4-hydroxyquinoline N-oxide (HQNO) was purchased from Ambeed. 2-Nonyl-4(*1H*)-quinolone (NHQ), 2-nonyl-3-hydroxy-4(*1H*)-quinolone (C_9-PQS) and *N*-hexanoyl-L-homoserine lactone-d_3 (C_6-HSL-d_3) were obtained from Cayman Chemical. 2-Nonyl-4-hydroxyquinoline n-oxide (NQNO) was obtained from Santa Cruz Biotechnology.

Preparation of culture supernatant extracts and standards for analysis. A 350 μL aliquot of culture suspension was centrifuged at 13,000 rpm, 5 min, 4 °C to pellet cells and debris. A 300 μL aliquot of the supernatant was transferred to a new tube, to which was added 3 μL of 100 μM C_6-HSL-d_3 internal standard in methanol. The resulting mixture was then extracted twice with an equal volume of acidified ethyl acetate (with 0.02% v/v acetic acid), by vigorous vortexing (1 min each time) at 3000 rpm and at ambient temperature. The upper organic layers were transferred to a clean glass vial, and the solvent was removed by rotatory evaporation in a 35 °C water bath. The resulting solid was resuspended in 900 μL of ice-cold methanol and transferred to a new ice-cold tube. The samples were prepared for mass spectrometry by centrifuging at 13,000 rpm, 10 min, 4 °C to pellet any particulates, following which 200 μL of the supernatant was transferred into a HPLC vial fitted with a polypropylene vial insert (Agilent #5182-0549).

Analyte standards (10 nM – 50 μM) were prepared by serial dilution of 1 – 10 mM stocks of each analyte freshly prepared by dissolving in methanol, with the exception of C_9-PQS which was instead dissolved into a 1:1 mixture of H_2O/0.1% formic acid (solvent A) and MeCN/0.1% formic acid (solvent B). Standards were used to prepare a standard curve and identify the effective range of detection (**Table S7**, see **Figure S34** for a representative example).

Triple quadrupole mass spectrometry. Triple quadrupole mass spectrometry was performed on an Ultivo triple quadrupole instrument coupled to an Agilent 1260 Infinity II LC system housed in the MIT Department of Chemistry Instrumentation Facility. For the quantification of alkylquinolones and homoserine lactones, the instrument was operated in multiple reaction monitoring (MRM) mode using an ESI source with positive polarity and the sample chamber was maintained at 4 °C. Samples were run on a Poroshell 120 EC-C18 column (Agilent, 695775-902) with the following gradient: 0 – 1 min: 10 % B, 1 –

1.2 min: 10 – 50% B, 1.2 – 5.2 min 50 – 99% B, 5.2 – 6.7 min: 99% B, 6.7 – 7.7 min: 99 – 10% B, 7.7 – 8.7 min: 10% B, 8.7 – 10.7 min: post-time. 2 µL of each sample was injected, and a flow rate of 0.4 mL/min was used.

MassHunter Optimizer (Agilent) software was used to determine optimal collision and fragmentation parameters for each compound (**Table S4**). For Optimizer runs, direct injection (without column) of 1 – 5 µL of 100 nM – 1 µM analyte was performed using 100%B at 0.3 mL/min for 2 min. The initial fragmentation voltage was set to 135 V for the alkylquinolones and 90 V for the homoserine lactones.

Microbiology

Growth media. All growth media, with the exception of chemically-defined medium (CDM), were purchased commercially, used as received, and prepared according to manufacturer directions. Luria-Bertani (LB, Miller) medium (BD Difco), *Pseudomonas* Isolation Agar (Sigma, BD Difco) and Baird-Parker medium (Sigma, BD Difco) were dissolved into Milli-Q water and sterilized prior to use. Sterilized Baird-Parker medium was completed by adding egg yolk tellurite emulsion (Sigma, BD Difco).

Chemically-defined medium (CDM). Metal-replete CDM was prepared as previously reported (1, 3). Briefly, metal-replete CDM was prepared by supplementation of unsupplemented CDM with the following metals immediately prior to use: 1 mM Ca(II), 0.3 µM Mn(II), 5 µM Fe(II), 0.1 µM Ni(II), 0.1 µM Cu(II) and 6 µM Zn(II). Mn-depleted, Fe-depleted and Zn-depleted CDM were prepared by omitting the respective metal from the aforementioned supplementation of unsupplemented CDM, and metal-depleted CDM was prepared by omitting Mn, Fe and Zn.

General methods for bacterial culture. *Pseudomonas aeruginosa* UCBPP-PA14 (provided by Professor Dianne Newman, CalTech) (4) and *Staphylococcus aureus* USA300 JE2 (provided by NARSA) (5) were cultured and handled as previously described (1). Culture turbidity measurements and colony-forming unit counting were performed as previously described (1).

RNA extraction and workup for RNA-seq. RNA extraction and workup was carried out as previously reported (1). After resuspension of the precipitated RNA samples in nuclease-free water, samples were submitted without further dilution to the MIT BioMicro Center for RNA-seq. Subsequent procedures leading up to and including sequencing were performed at the MIT BioMicro Center. Prior to library preparation, sample integrity was validated using fluorescence-based electrophoresis (AATI Fragment Analyzer), following which ribosomal depletion was performed using the NEB Next® rRNA Depletion Kit (New England Biolabs). Subsequently, library preparation (adapter ligation, size selection, barcoding, enrichment) was performed using the NEBNext Ultra II Directional RNA Library Prep Kit (New England Biolabs). The quality of the resulting libraries was validated using real-time PCR, following which the libraries were pooled and sequenced on a single lane of an Ilumina NextSeq500 instrument using 75 nt chemistry.

Real-time PCR. RNA was extracted for real-time PCR as previously reported (1). To determine the appropriate timepoint for RNA-seq, real-time PCR was performed on *P. aeruginosa* and *S. aureus* housekeeping genes (*P. aeruginosa*: *16S*, *S. aureus*: *sigA*) as a readout of transcript levels (1). Real-time PCR was performed on a select panel of *P. aeruginosa* genes based on differential expression trends observed from RNAseq (**Table S5**). Standard curves to determine the dynamic range of detection for real-time PCR were performed as previously reported (**Table S6**) (1).

Bioinformatics Workflow and Analyses

Read alignment and feature quantification. Raw reads were aligned using the hisat2 aligner (6) using reference genomes obtained from the NCBI RefSeq database for *P. aeruginosa* PA14 (GCF_000014625.1) (7) and *S. aureus* JE2 (GCF_002085525.1) (Walter Reed Army Institute of Research). The combined genome derived from both PA14 + JE2 genomes was used as the reference to align reads from coculture samples. For read alignment, the mapping fidelity associated with each species was verified by ensuring no cross-mapping or off-target mapping for both species. Next, the aligned reads were quantified using Feature Aggregate Depth Utility, which is designed specifically for feature quantification from prokaryotic sample reads (8). To ensure samples were sequenced to sufficient depth, rarefaction analyses were performed on the resulting counts using the R package "vegan" (9). To detect the presence of technical artifacts and systematic errors, principal component analysis was also performed in R (version 4.3.1). Quantification of *S. aureus* sRNAs was performed using the available annotations for *S. aureus* USA300 FPR3757 on the *Staphylococcus* Regulatory RNAs database (10). To obtain equivalent annotations for sRNA coding sequences in *S. aureus* JE2, CD-HIT-EST (11) was used to obtain unique gene mappings for features in both *S. aureus* genomes.

Differential expression (DE) analysis and clustering. DE analysis of the quantified reads was performed using DESeq2 (version 1.40.2) (12) with user-supplied metadata on experimental conditions and sample batches. For both monocultures and cocultures, untreated cultures (in metal-replete CDM) served as the untreated control. Prior to analysis with DESeq2, genes detected in less than three samples and with normalized counts less than ten were omitted. Log_2(fold changes) (LFCs) were calculated using the apeglm method for effect size shrinkage (13), and were used to construct volcano plots for understanding transcriptome-wide changes on gene expression. Next, for the purposes of clustering, LFCs were subjected to the variance-stabilizing *rlog* transformation (12, 14). Hierarchical clustering was performed with the variance-stabilized LFCs using the "pheatmap" package (version 1.0.12) in R.

Functional enrichment analyses. Functional enrichment analyses were performed with clusterProfiler (15, 16) using the variance-stabilized LFCs for both species. Two approaches were used for functional enrichment analyses. Overrepresentation analysis (ORA) enabled detection of Gene Ontology (GO) terms enriched in the shortlist of highly DE genes. Gene set enrichment analyses enabled transcriptome-wide detection of GO terms that may not fall within the threshold cutoff for ORA, or in cases where ORA cannot obtain statistically significant results (*vide infra*). For *P. aeruginosa* PA14, the manually curated PAO1 GO annotation available on the *Pseudomonas* genome database (pseudomonas.com) (17) was used as it contained the most updated GO annotations as compared to prior NCBI RefSeq entries for *P. aeruginosa* PA14. CD-HIT-EST was used to obtain unique gene mappings between the *P. aeruginosa* genomes. For *S. aureus* JE2, the GO annotations available on the associated NCBI RefSeq entry were used.

Cross-mapping analysis. The frequency of off-target mapping was determined by aligning two representative datasets, one from a monoculture of *P. aeruginosa* and one from a monoculture of *S. aureus*, to the combined *P. aeruginosa* + *S. aureus* reference genome. Cross-mapping analyses across all conditions found approximately 15 *P. aeruginosa* genes and 70 *S. aureus* genes (with at least 5 counts, but no more than 40 counts) that were detected to be mapping off-target. To determine if cross-mapping might affect downstream analyses, the number of reads for each cross-mapped gene was compared against the number of counts of the untreated control of the intended species, flagging hits that exceeded the strict threshold of >0.05% of the control counts. While a vast majority of cross-mapping genes did not exceed this threshold, this filter identified six low-count genes, which comprised five *P. aeruginosa* genes and one *S. aureus* gene, which were flagged but not removed for downstream analyses. Only two of the six genes were annotated.

These genes were *P. aeruginosa pvdA* and *pvdP*, which are in the operon encoding for pyoverdine biosynthesis (~0.08% of the untreated control counts).

General notes on bioinformatics analysis. Given limitations on the available gene annotations for *S. aureus* JE2 at the time of this study, we utilized the more extensive annotations of *S. aureus* NCTC 8325 and *S. aureus* USA300 FPR3757. To use these annotations, we also employed CD-HIT-EST to map and derive gene annotations across *S. aureus* strains, in conjunction with the online *S. aureus* genome database *Aureo*Wiki, (aureowiki.med.uni-greifswald.de) (18). In addition, functional enrichment analyses for *S. aureus* were performed using gene set enrichment analysis (using the available annotations for *S. aureus* USA300 FPR3757) instead of overrepresentation analysis due to the inability of overrepresentation analysis to obtain statistically significant clusters (obtained adjusted p-values of 0.4 or larger). We attribute the poor performance of overrepresentation analysis to systemic differences between *S. aureus* gene expression across the conditions tested. In contrast, we found that gene set enrichment analysis provided clustering results of improved statistical significance.

SUPPLEMENTARY DISCUSSION

Distinct transcriptional responses of *P. aeruginosa* to CP treatment and metal depletion

P. aeruginosa transcripts were sequenced to saturation (**Table S3**), with at least 99.8% of unique genes in the *P. aeruginosa* genome captured across all conditions tested.

CP treatment – effects on *P. aeruginosa* in monoculture and coculture

CP treatment and Fe depletion decreased the expression of *ccpR*, which encodes a cytochrome c551 peroxidase (19). CP treatment also altered the expression of genes encoding for multiple ferredoxins and the ferredoxin-NADP$^+$ reductases *fpr* and *fprB*/*fnr-2* (20–22) in a manner that could not be attributed to metal depletion (**Figure S6**). CP treatment decreased the expression of genes encoding for a putative di/tripeptide ABC transporter system (*PA14_58420 – PA14_58490*) (23), an effect that was observed but attenuated in Fe-depleted cocultures (**Table SF10**). CP treatment increased the expression of genes associated with the type III secretion system (T3SS) (*exsA*, *PA14_42250 – PA14_42480*) (24) (**Figure S25**), an effect that was partially attributable to Fe depletion. Although genes encoding for alkaline protease secretion (*aprDEX*) (25, 26) were upregulated in response to both CP treatment and Fe depletion, the alkaline protease gene *aprA* (27) was downregulated in response to CP treatment and upregulated in Fe-depleted conditions (**Figure S25**).

We found that a cluster of *P. aeruginosa* genes were strongly upregulated only under conditions of CP treatment, which included the operonic pair of *ilvA2* (encodes threonine dehydratase) and the hypothetical gene *yybH* (*PA14_47110*) (**Figure S26**). This finding was reminiscent of prior studies examining a genetic mutant of the elongation factor FusA1 in *P. aeruginosa* PAO1 where the same genes were identified as the most upregulated genes (28). Other unique responses of *P. aeruginosa* to CP treatment observed in our study, including strong downregulation of the *mexGHI-opmD* RND-family efflux pump (29, 30) and upregulation of both the sulfate transport machinery *cysAWT* (31) and the organosulfur utilization system *ssu* (32), were also identified in this prior work (28) (**Figure S26**).

In addition, we observed that translational and RNA processing machineries were overall upregulated for *P. aeruginosa* in the presence of CP (**Figure S27**), with generally no significant change observed in all other conditions. The carbon catabolite repression gene *crc* (33–35) was upregulated in the presence of CP (**Figure S27**). In addition, the sigma factor associated with stationary phase character *rpoS* (36–38) was downregulated in response to CP treatment (**Figure S27**) despite similar growth dynamics and viability of both *P. aeruginosa* and *S. aureus* at this timepoint (1). These observations were supported by real-time PCR data demonstrating that CP treatment decreased the expression of the *crcZ* sRNA (39), a positive regulator of catabolite repression, and *rpoS* (**Figure S28**). Real-time PCR also showed that the expression of the ribosome modulation factor *rmf* (40), which inhibits translation, was decreased in the presence of CP (**Figure S28**). While the redirected chorismate flux is likely to contribute to these expression changes, how CP affects translation in *P. aeruginosa* remains to be elucidated.

Fe depletion – effects on *P. aeruginosa* in coculture

The expression of several genes known to be regulated by *pprAB* was increased for cocultures grown in Fe-depleted CDM (41) (**Table SF5**). Beyond these findings, no systems that were DE specifically in response to Fe depletion in *P. aeruginosa* were identified.

Zn depletion – effects on *P. aeruginosa* in coculture

Genes encoding for the molecular chaperone GroES were upregulated in response to Zn depletion (**Table SF6**). No other known *P. aeruginosa* genes that were DE specifically in response to Zn depletion were detected.

Mn depletion – effects on *P. aeruginosa* in coculture

Mn starvation responses for *P. aeruginosa* have not been characterized to date (42). Mn depletion resulted in modest upregulation of several genes within operons encoding for type VI secretion systems (T6SS) in *P. aeruginosa* (H1-T6SS: *PA14_01010 – PA14_01110* (43), H2-T6SS: *PA14_42890 – PA14_*

43050 (44)) (**Figure S29**). However, based on our prior coculture studies (1), these transcriptional changes do not appear to significantly impact the antistaphylococcal activity of *P. aeruginosa* in coculture under the culture conditions used in this study and the prior work. We also identified several genes that were upregulated by a small (0.5 < Log_2(Fold Change) < 1) but significant extent in response to Mn depletion (**Table SF7A**), which did not meet the DE threshold.

Mn depletion decreased expression of *argD*, which encodes for *N*-succinylglutamate 5-semialdehyde dehydrogenase and is involved in arginine and proline metabolism (45). *argD* was also downregulated in response to Fe depletion (**Figure S29**). In addition, Mn depletion led to upregulation of *PA14_58410* (annotated as *opdP* in PAO1) (46, 47), which encodes a putative outer membrane porin involved in arginine uptake, and the branched-chain amino acid transport machinery *bra* (48) (**Figure S29**). Together, these findings suggest that Mn depletion may alter nutrient preference and utilization in *P. aeruginosa* cocultured with *S. aureus*.

CP treatment – effects on *P. aeruginosa* in monoculture

In response to CP treatment, we observed that genes associated with the type III secretion system were more strongly upregulated in monoculture than in coculture (**Table SF8A**). Overrepresentation analysis indicated that the GO term for acyl-transferase activity was enriched amongst downregulated genes for *P. aeruginosa* in CP-treated monocultures (**Figure S5D**). The genes identified by this analysis included the short-chain acyl-CoA synthetase *bkd* (33, 49) and the short-chain fatty acid degradation system *ato* (50), which were found to be more strongly downregulated by CP treatment in monoculture as compared to in coculture (**Figure S30** and **Table SF10**). These findings suggest that CP treatment leads to dysregulation of fatty acid metabolism that is more pronounced in monoculture than in coculture.

Several other genes associated with glycolytic flux and amino acid utilization were observed to be downregulated only or much more strongly in CP-treated monocultures. These genes included *mmsB* (valine metabolism) (51), the *gtrS*-*gltR*-*glk* operon (52, 53), as well as the adjacent *edd* gene (54) (**Figure S30** and **Table SF8B**). Together, these observations indicate decreased glycolytic flux and increased

demand for amino acids (49) by *P. aeruginosa* in CP-treated monocultures. Furthermore, the expression of genes associated with protein homeostasis, including several genes encoding for heat shock proteins and chaperones (**Figure S30**), was downregulated in CP-treated monocultures and, to a lesser extent, in CP-treated cocultures. We tentatively propose that the effects of CP treatment on *P. aeruginosa* in monoculture are likely linked to effects on translational machinery (**Figure S27**) as CP treatment does not significantly alter *P. aeruginosa* viability or growth dynamics in coculture (1).

Distinct transcriptional responses of *S. aureus* to CP treatment and metal depletion

RNAseq captured 94 – 96% of unique *S. aureus* genes across all conditions, with the exception of CP-treated cocultures where *S. aureus* was sequenced to saturation (**Table S3**); sequencing depth generally correlated with library size. These findings indicate that some degradation of *S. aureus* RNA occurred in the untreated cocultures, consistent with significant killing of *S. aureus* by *P. aeruginosa* in the absence of CP treatment (1). To avoid problematic comparisons, genes that did not meet the minimum detection threshold were excluded from further downstream analyses (12.7% of total genes).

CP elicits multi-metal starvation responses in *S. aureus* cocultured with *P. aeruginosa*

For *S. aureus* cocultures treated with CP or grown in Fe-depleted CDM, we found that only about 18% of upregulated genes (**Figure 8A**) and 27% of downregulated genes (**Figure 8B**) were common to both conditions, indicating that the transcriptional responses of *S. aureus* to CP treatment and Fe depletion differ considerably. For cocultures grown in Zn-depleted CDM, only a small number of upregulated genes (~ 0.6%) were detected (**Tables SF19**), a majority of which were also detected in CP-treated cocultures (**Figure S18**). No downregulated *S. aureus* genes were found to meet the significance and DE thresholds for Zn-depleted cocultures (**Table S2**). For cocultures grown in Mn-depleted CDM, we observed only partial overlap between DE genes in response to CP treatment and Mn depletion (**Figure S16**). The transcriptional responses of *S. aureus* to the depletion of one metal (Mn, Fe or Zn) were largely recapitulated in metal-depleted cocultures (**Figures S16 – S18**). Although most DE genes in CP-treated monocultures

overlapped with those in CP-treated cocultures, a significant proportion of transcriptional responses to CP were observed only for *S. aureus* in coculture (**Figures S19A** and **S19B**). Gene set enrichment analysis revealed multiple GO terms that were enriched in both CP-treated and metal-depleted cocultures, and identified several groups of GO terms which responded uniquely to each condition (**Figure S20**). We note that the p-values for these GO terms obtained were relatively large due to the limited annotation of *S. aureus* genomes (see **Supplemental Experimental**) and variation between the transcriptional responses of *S. aureus* across culture conditions, such as in Zn-depleted cocultures (*vide infra*).

CP treatment – effects on *S. aureus* in monoculture and coculture

CP treatment and Zn depletion resulted in the upregulation of genes associated with staphylopine biosynthesis and transport machinery (*cnt*, *B7H15_13700 – B7H15_13715*) (55, 56) (**Figure S31** and **Table SF13**). These findings suggest that increased staphylophine production by *S. aureus* is part of a Zn-starvation response. CP treatment and Fe depletion downregulated the expression of genes encoding for nitrate reductase (*nar*) (57) (**Figure 8B**). CP treatment resulted in upregulation of *glpT*, which encodes a glycerol-3-phosphate transporter (58) and two genes involved in gluconate uptake and utilization (*gntPK*) (59) (**Figure S24**).

Fe depletion – effects on *S. aureus* in coculture

We also identified transcriptional changes for cocultures subjected to Fe depletion that were not observed in CP-treated cocultures (**Table SF15**), which included upregulation of genes associated with general oxidative stress responses (*ahpF*, *katA*, *sodM*, *msrAB*) (60) (**Figure S21**), and the downregulation of genes associated with type 8 capsular polysaccharide synthesis (*cap*) and translational machinery (**Figures S21** and **S22**). These effects were also identified in Mn-depleted cocultures. In addition, expression of the methicillin resistance gene *mecA* (61) was decreased and the expression of genes encoding for urease (62) was upregulated only in response to Fe depletion (**Figure S21**). Fe depletion also decreased the expression of genes associated with the *agr* master virulence regulator, ATP synthase and DNA

replication (**Table SF15B**). Fe depletion also resulted in downregulation of genes encoding for the SrrAB two-component system, which is involved in metabolic adaptations and responses of *S. aureus* to changes in respiration (63).

Zn depletion – effects on *S. aureus* in coculture

CP treatment and Zn depletion upregulated expression of the Zn uptake regulator *zur* (*B7H15_08535*) (**Figure S31** and **Table SF19**); the presence of CP resulted in a small but significant increase of *zur* in coculture. Zn depletion resulted in the upregulation of the Zn-specific ABC permease *adcA* (56) and a Zur-regulated transporter (annotated as a second ferrous ion transporter, *B7H15_14360*) (**Figure S31** and **Table SF19**); expression levels of these genes were not significantly changed in CP-treated cocultures and were found to be upregulated in CP-treated monocultures.

Mn depletion – effects on *S. aureus* in coculture

CP treatment and Mn depletion resulted in upregulation of the manganese ABC transporter substrate-binding protein and permease *mntAB* (64), which was upregulated to a lesser extent in Fe-depleted cocultures. The presence of CP and Mn limitation led to upregulation of the *arlRS* two-component system (65–67), which is involved in resistance against Mn starvation (**Figure S32**, **Table SF19**), although the change in expression of *arlRS* in response to Mn depletion fell just below the DE threshold. Beyond these findings, we detected only minor overlap between the transcriptional responses of *S. aureus* in coculture to CP treatment and Mn depletion (**Figure S16 and Table S14**). We detected several *S. aureus* systems that were DE only in response to Mn depletion, which primarily consisted of genes involved in amino acid utilization (**Figure S32**). *S. aureus* in Mn-depleted cocultures exhibited upregulation of genes involved in arginine utilization processes (*arc*, *arg*) (68) and the proline transporter gene *proP* (69, 70).

CP treatment – effects on *S. aureus* in coculture

Common to CP treatment and Fe depletion, the expression of genes involved in the biosynthesis and transport of biotin (*bio*) was upregulated (**Figure 8A, 8C** and **Table SF12A**). Genes associated with tryptophan and anthranilate biosynthesis (*trp*), staphyloxanthin biosynthesis (*crt*) (71), quinol oxidase (*qox*) (72) and the Na^+/H antiporters (*mnh*) (73) were downregulated for *S. aureus* in CP-treated and Fe-depleted cocultures (**Figures 8B, 8C** and **Table SF12B**). These collective responses are likely a response of *S. aureus* to Fe starvation.

CP treatment – effects on *S. aureus* in monoculture

A small proportion of genes was found to be DE only for *S. aureus* in monoculture (**Table SF18**). Genes associated with the conversion of pyruvate to formate *(pflAB*) were upregulated in CP-treated monocultures (74) (**Figure S33**). However, the expression of other genes associated with fermentation, such as alcohol dehydrogenase and lactate dehydrogenase, remained unchanged (49, 75) (**Figure S33**). Furthermore, expression of the gene encoding for the catabolite control protein A (*ccpA*) was unchanged (**Figure S33**). Together, these findings suggest that CP treatment may increase formate demand in *S. aureus* monocultures without inducing an overall shift in respiration towards fermentation. CP-mediated upregulation of genes associated with purine and pyrimidine biosynthesis was found to be attenuated in monoculture as compared to coculture, which may result from differences in growth phase (1).

Other genes that were upregulated in CP-treated monocultures include genes encoding for the glycerophosphodiester phosphodiesterase *glpQ* (76, 77), alpha-glucosidase (*B7H15_08295*) (78), and a putative oligopeptide permease (*B7H15_00390 – B7H15_400*, assigned as *opp*3) involved with peptide import (79, 80) (**Figure S33**). Other genes that were downregulated only in CP-treated monocultures include the *ssp* operon (81), which encodes for the V8 serine protease and staphopain B, and the sirohydrochlorin ferrochelatase *B7H15_13305* (82) (**Figure S33**).

Zn availability contributes to the metabolism of *S. aureus* cocultured with *P. aeruginosa* when part of a multi-metal starvation response

Intriguingly, many *S. aureus* genes that were DE in response to CP treatment, Fe depletion or Mn depletion were not DE in response to Zn depletion (**Figures 9, S18, S21, S22** and **S24**), which included genes responsive to cell wall damage and genes associated with translational machinery. These observations indicate that Zn depletion alone does not significantly impact the killing of *S. aureus* by *P. aeruginosa*, in agreement with our prior work (1). The observation that *S. aureus* is able to mount partial Fe-starvation responses (**Figure 9**) when cocultured with *P. aeruginosa* under metal-depleted conditions led us to hypothesize Zn depletion likely facilitates sufficient metabolic activity for *S. aureus* to mount Fe-starvation responses when part of a multimetal-starvation response (**Figure S21 and S22**). This idea is reminiscent of a prior study showing that CP-mediated Zn sequestration decreased the proteolytic activity of *P. aeruginosa* supernatants via effects on Zn(II)-dependent proteases (83). Given that we identified only limited transcriptional responses to Zn depletion (**Figure S18** and **Table S3**), our findings suggest that Zn availability likely contributes to, but is not a key determinant of, *S. aureus* viability in coculture under our experimental conditions.

SUPPLEMENTARY TABLES

Table S1. Detection of transcripts for the *S. aureus* housekeeping gene *sigA*. RNA was extracted from cultures grown in metal-replete CDM ± 20 μM CP and incubated at 37 °C for 6 – 8 h.

	−CP			+CP		
Timepoint/ Biological Replicate	#1	#2	#3	#1	#2	#3
6 h	26.5	28.5	26.8	21.8	23.5	22.5
7 h	33.2	31.8	29.4	23.4	24.2	24.7
8 h	33	>35	>35	24.5	24.3	25.0

Table S2. The proportion of differentially expressed *P. aeruginosa* and *S. aureus* genes across conditions. Genes which met the threshold cutoff of at least 1 Log_2(Fold Change) from the untreated control are represented.

	P. aeruginosa		*S. aureus*	
Condition	Proportion of upregulated genes (%)	Proportion of downregulated genes (%)	Proportion of upregulated genes (%)	Proportion of downregulated genes (%)
Mono+CP	10.9	12.8	4.69	2.80
Co+CP	12.1	13.6	21.8	18.5
Co-Fe	4.07	5.53	21.9	20.7
Co-Mn	0.74	0.12	3.10	2.40
Co-Zn	2.12	0.22	0.63	0.00
Co-dep	5.53	6.51	12.5	14.3

Table S3. Key statistics and average size of *P. aeruginosa* and *S. aureus* libraries obtained from RNAseq.

	P. aeruginosa		*S. aureus*	
Condition	Proportion of unique genes detected (% of genome)	Average library size (million)	Proportion of unique genes detected (% of genome)	Average library size (million)
Mono-CP	99.85	13.14	99.73	18.23
Mono+CP	99.88	12.29	99.69	20.77
Co-CP	99.87	11.18	96.74	1.69
Co+CP	99.82	4.31	99.73	11.54
Co-Fe	99.83	12.01	93.99	0.74
Co-Mn	99.87	6.43	94.03	0.61
Co-Zn	99.85	12.96	96.61	1.34
Co-dep	99.85	10.72	96.57	1.49

Table S4. Optimized collision and fragmentation parameters for the selected analytes.

Analyte	Precursor ion (*m/z*)	Product ion (*m/z*)	Fragmentor voltage (V)	Collision energy (V)
3-oxo-C_{12}-HSL	298.1	102.1	94	12
C_4-HSL	172.1	102.1	70	4
C_9-PQS	288.1	175.1	146	36
HHQ	244.1	159.1	138	32
HQNO	260.1	159.1	126	32
NHQ	272.1	159.1	150	40
NQNO	288.1	159.1	150	32
PQS	260.1	175.1	130	32
C_6-HSL-d_3	203.1	102.0	70	8

Table S5. Primers used for real-time PCR of *P. aeruginosa*.

Primer	Sequence (5' to 3')	Reference
16S.For	GGAGAAAGTGGGGGATCTTC	(84)
16S.Rev	CCGGTGCTTATTCTGTTGGT	(84)
lasA.For	GTTGTAGAGCAGCGAGAAATG	-
lasA.Rev	CTATGCCAGCAACATCAACAC	-
lasB.For	CAACCAGAAGATCGGCAAGTA	-
lasB.Rev	GTTCATGTCGACGGTGATGA	-
rhlA.For	CATTTCAACGTGGTGCTGTTC	-
rhlA.Rev	TTCCACCTCGTCGTCCTT	-
pqsD.For	CCATGTTGCCCAGACGAT	-
pqsD.Rev	ACCATGTGATCTGCCATCAA	-
pqsE.For	CAGTGGTCGTAGTGCTTGT	-
pqsE.Rev	GGATGCCGAATTGGTTTGG	-
rmf.For	CTGGAGTTGATTGAGACGTTGTA	-
rmf.Rev	CTCGTGATCTTTGTCCGTTCA	-
rpoS.For	CGGAGTTTGACCACGATGAT	-
rpoS.Rev	GAGAAGGAAGTGGTGGCTTT	-
crcZ.For	GCCAGTCGGAAGAAGAATAA	-
crcZ.Rev	CCAGGCTGGGAGTTCAATAG	-

Table S6. Standard curves for real-time PCR of *P. aeruginosa* genes associated with quorum-sensing, alkylquinolone production and translational activity.

**Gene**	**Conc. Range (Metal-replete) (ng/μL)**	**R^2 value**	**Conc. Range (Metal-replete +CP) (ng/μL)**	**R^2 value**	**Conc. Range (Fe-depleted) (ng/μL)**	**R^2 value**
16S	10–300[1]	0.9827[1]	10–600[1]	0.9981[1]	1–300	0.9879
lasA	1–100	0.9964	1–100	0.9948	1–300	0.9938
lasB	1–100	0.9891	1–100	0.9891	1–100	0.9988
rhlA	1–100	0.9917	1–100	0.9934	1–300	0.9965
pqsD	1–100	0.9950	1–300	0.9859	1–600	0.9957
pqsE	1–100	0.9936	1–100	0.9892	1–100	0.9905
rmf	1–100	0.9872	10–300	0.9821	1–300	0.9998
rpoS	1–100	0.9938	1–100	0.9911	1–300	0.9997
crcZ	1–600	0.9939	1–100	0.9956	1–100	0.9891

Table S7. Effective dynamic range and standard curve fitting of analytes quantified by triple quadrupole mass spectrometry.

**Analyte**	**Conc. Range (nM)**	**R^2 value**
3-oxo-C_{12}-HSL	10–10000	0.9965
C_4-HSL	10–10000	0.9964
C_9-PQS	10–25000	0.9991
HHQ	10–5000	0.9921
HQNO	10–10000	0.9949
NHQ	10–10000	0.9910
NQNO	10–10000	0.9952
PQS	25–50000	0.9992
C_6-HSL-d_3	10–10000	0.9968

SUPPLEMENTARY FIGURES

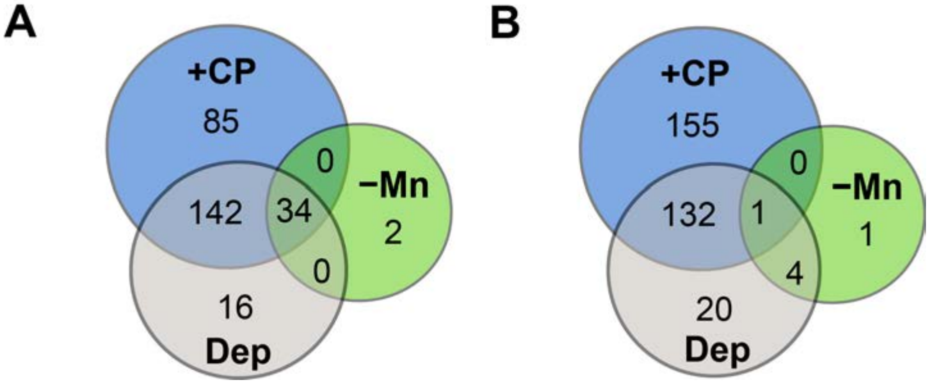

Figure S1. Transcriptional responses to Mn depletion overlap with responses to CP treatment in *P. aeruginosa* cocultured with *S. aureus*. Venn diagrams of the top 600 differentially expressed genes across all culture conditions reveals significant overlap for upregulated (**A**) genes in CP-treated, Mn-depleted and metal-depleted (Dep) cocultures, although only a small number of genes were found to be downregulated (**B**) in Mn-depleted cocultures.

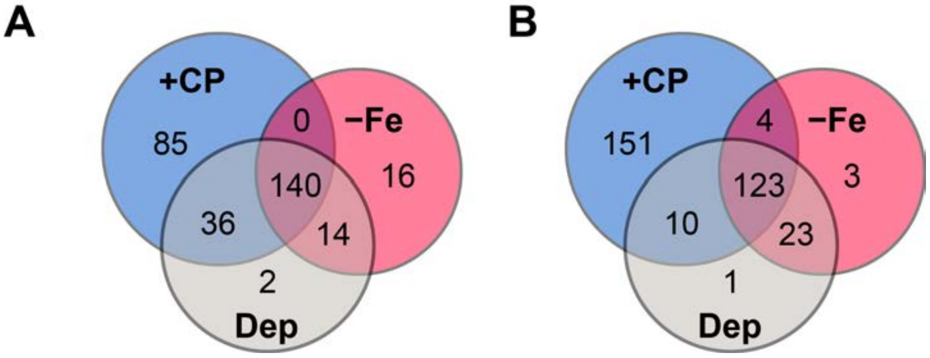

Figure S2. Transcriptional responses to Fe depletion overlap significantly with responses to CP treatment in *P. aeruginosa* cocultured with *S. aureus*. Venn diagrams of the top 600 differentially expressed genes across all culture conditions reveals significant overlap for upregulated (**A**) genes and downregulated (**B**) genes in CP-treated, Fe-depleted and metal-depleted (Dep) cocultures.

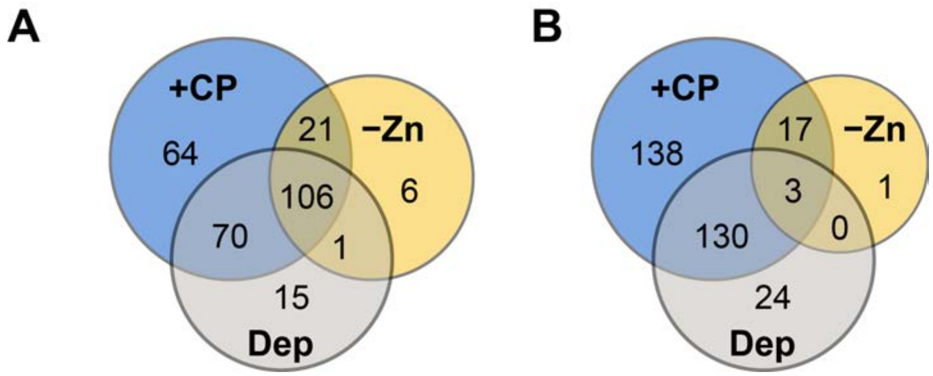

Figure S3. Transcriptional responses to CP treatment and Zn depletion overlap significantly in *P. aeruginosa* cocultured with *S. aureus*. Venn diagrams of the top 600 differentially expressed genes across all culture conditions reveals significant overlap for upregulated (**A**) genes in CP-treated, Zn-depleted and metal-depleted cocultures and downregulated (**B**) genes in CP-treated and Zn-depleted cocultures.

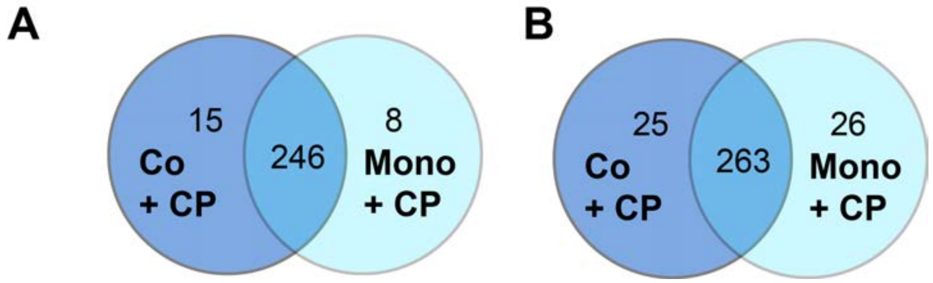

Figure S4. Transcriptional responses of *P. aeruginosa* in coculture with *S. aureus* to CP resemble those seen for *P. aeruginosa* in monoculture. Venn diagrams of the top 600 differentially expressed genes across all culture conditions reveals large similarities in both upregulated (**A**) genes and downregulated (**B**) genes resulting from CP treatment of *P. aeruginosa* in monoculture and in coculture with *S. aureus*.

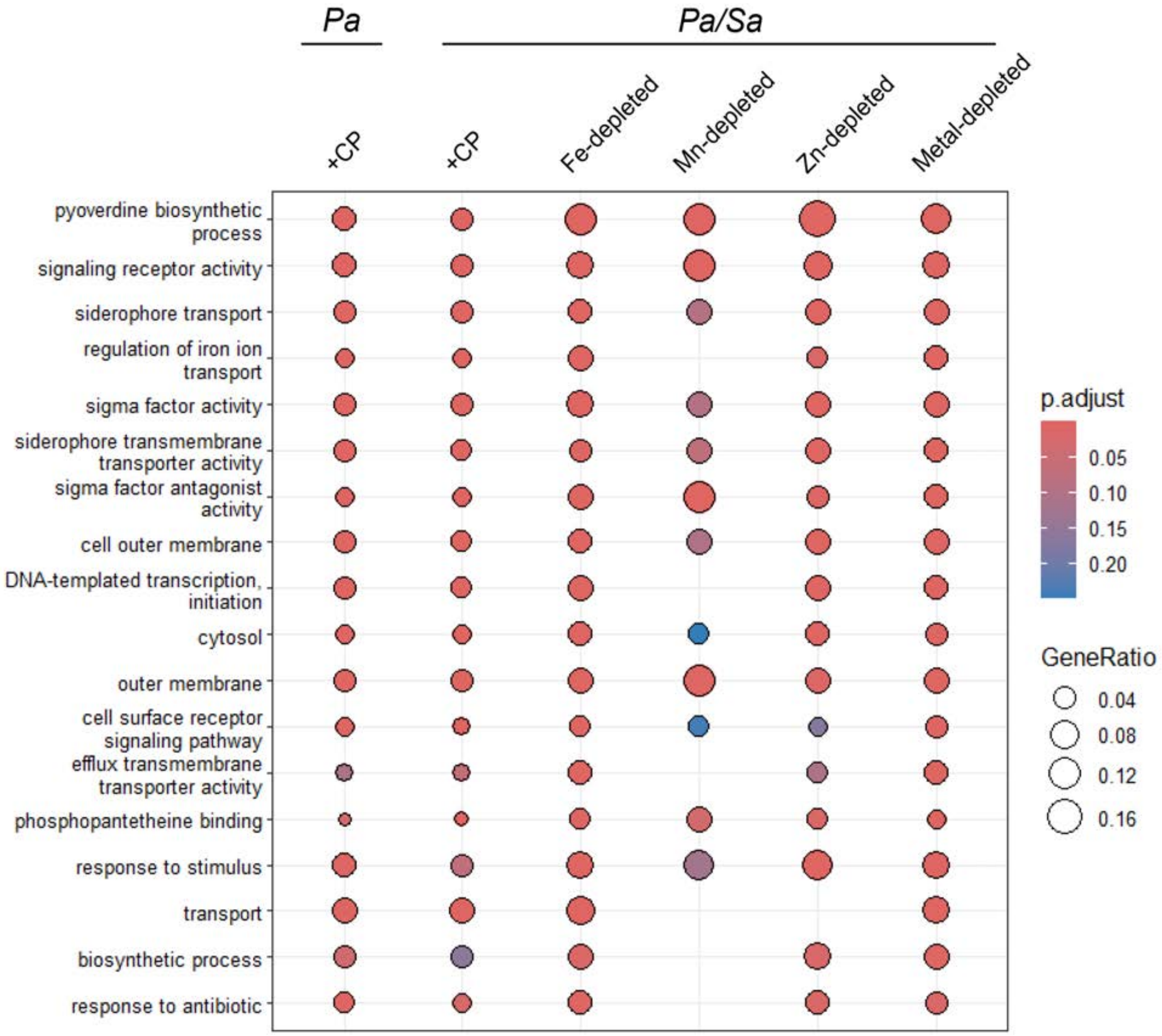

Figure S5A. Overrepresentation analysis of upregulated *P. aeruginosa* genes across conditions reveals GO terms enriched across multiple conditions. P-values were calculated using the Benjamini-Hochberg method, and the gene ratio represents the proportion of genes out of all genes considered for the tested condition. *Pa* indicates *P. aeruginosa* monoculture and *Pa/Sa* indicates the coculture. GO terms that were enriched for *P. aeruginosa* in monoculture and in coculture ($p < 0.2$) and in at least one other condition are shown.

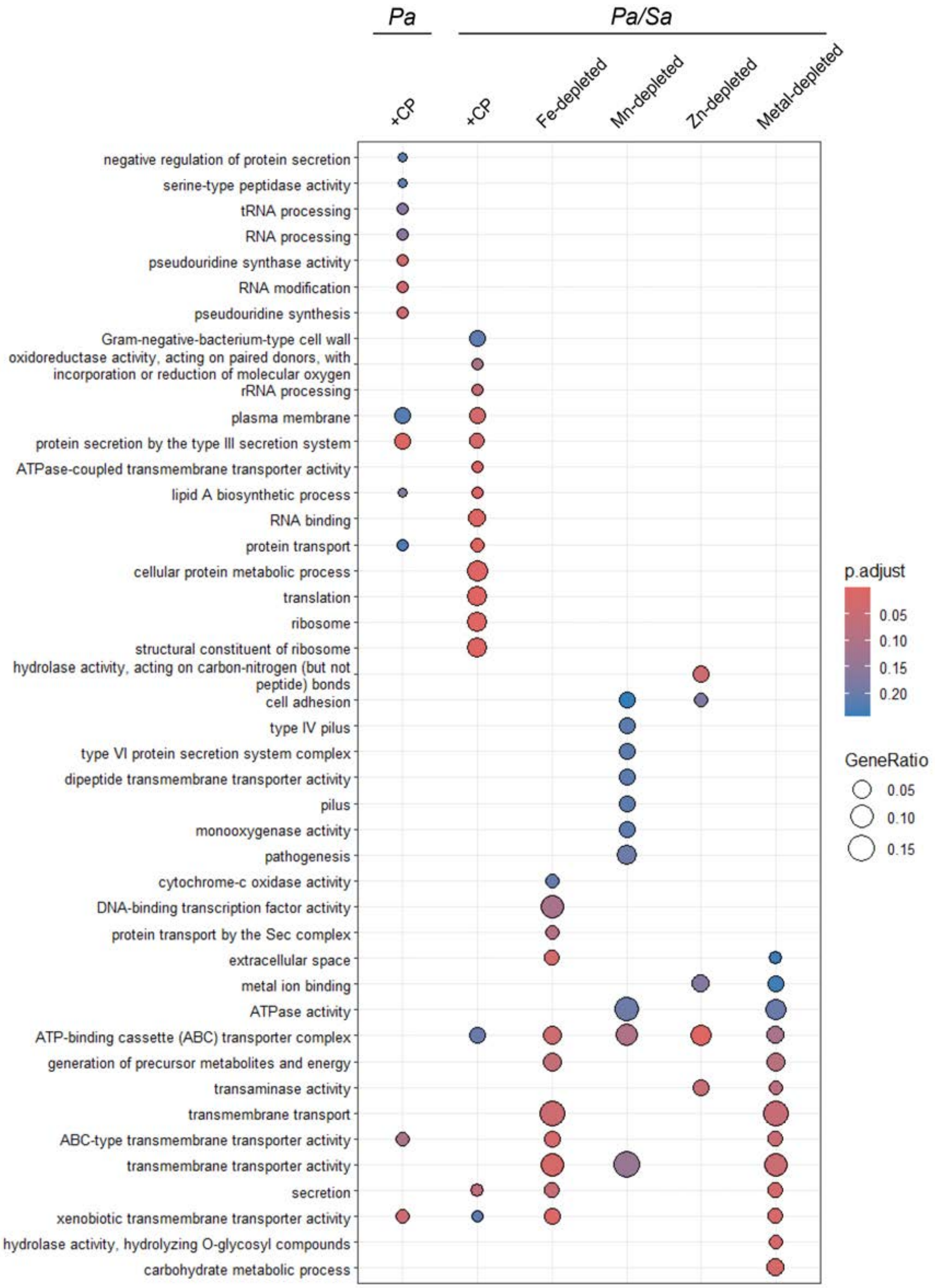

Figure S5B. Overrepresentation analysis of upregulated *P. aeruginosa* genes across conditions. P-values were calculated using the Benjamini-Hochberg method, and the gene ratio represents the proportion of genes out of all genes considered for the tested condition. *Pa* indicates *P. aeruginosa* monoculture and *Pa/Sa* indicates the coculture.

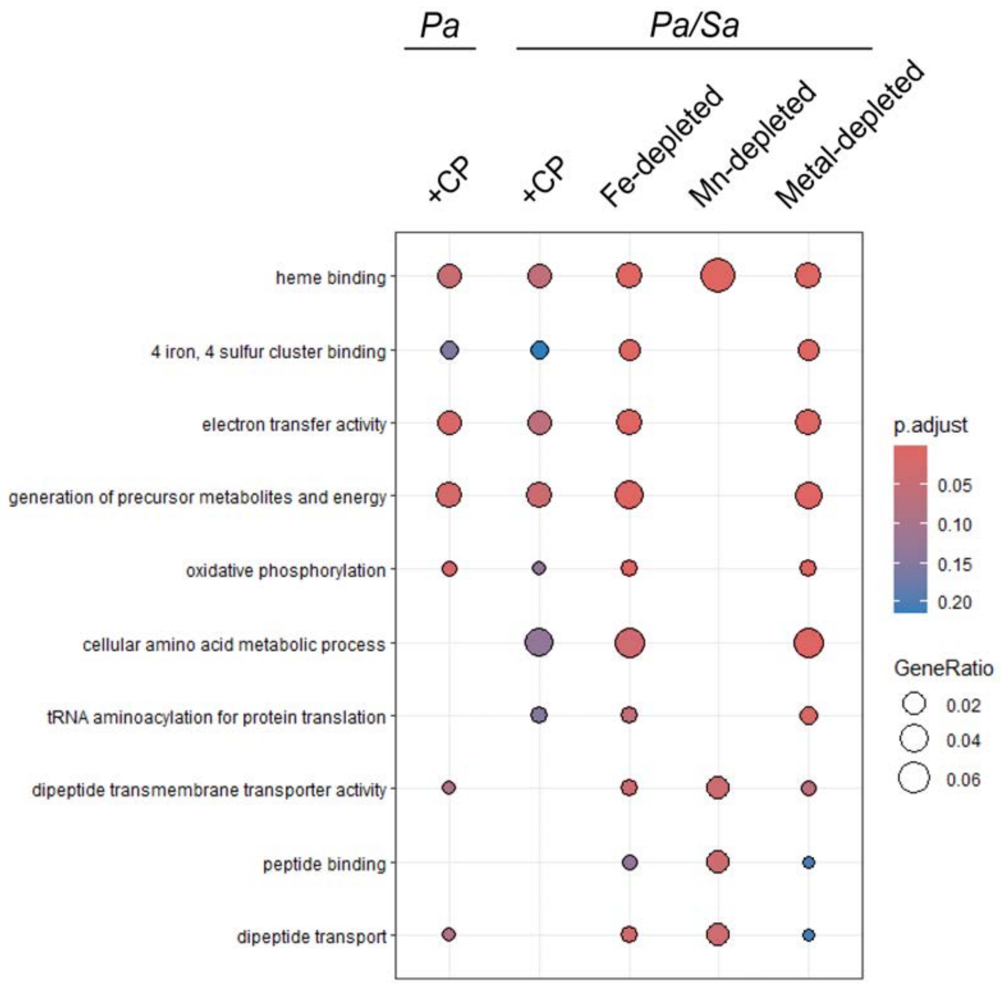

Figure S5C. Overrepresentation analysis of downregulated *P. aeruginosa* genes across conditions reveals GO terms enriched across multiple conditions. P-values were calculated using the Benjamini-Hochberg method, and the gene ratio represents the proportion of genes out of all genes considered for the tested condition. *Pa* indicates *P. aeruginosa* monoculture and *Pa/Sa* indicates the coculture. GO terms that were enriched for *P. aeruginosa* in the presence of CP and in at least two other conditions are shown.

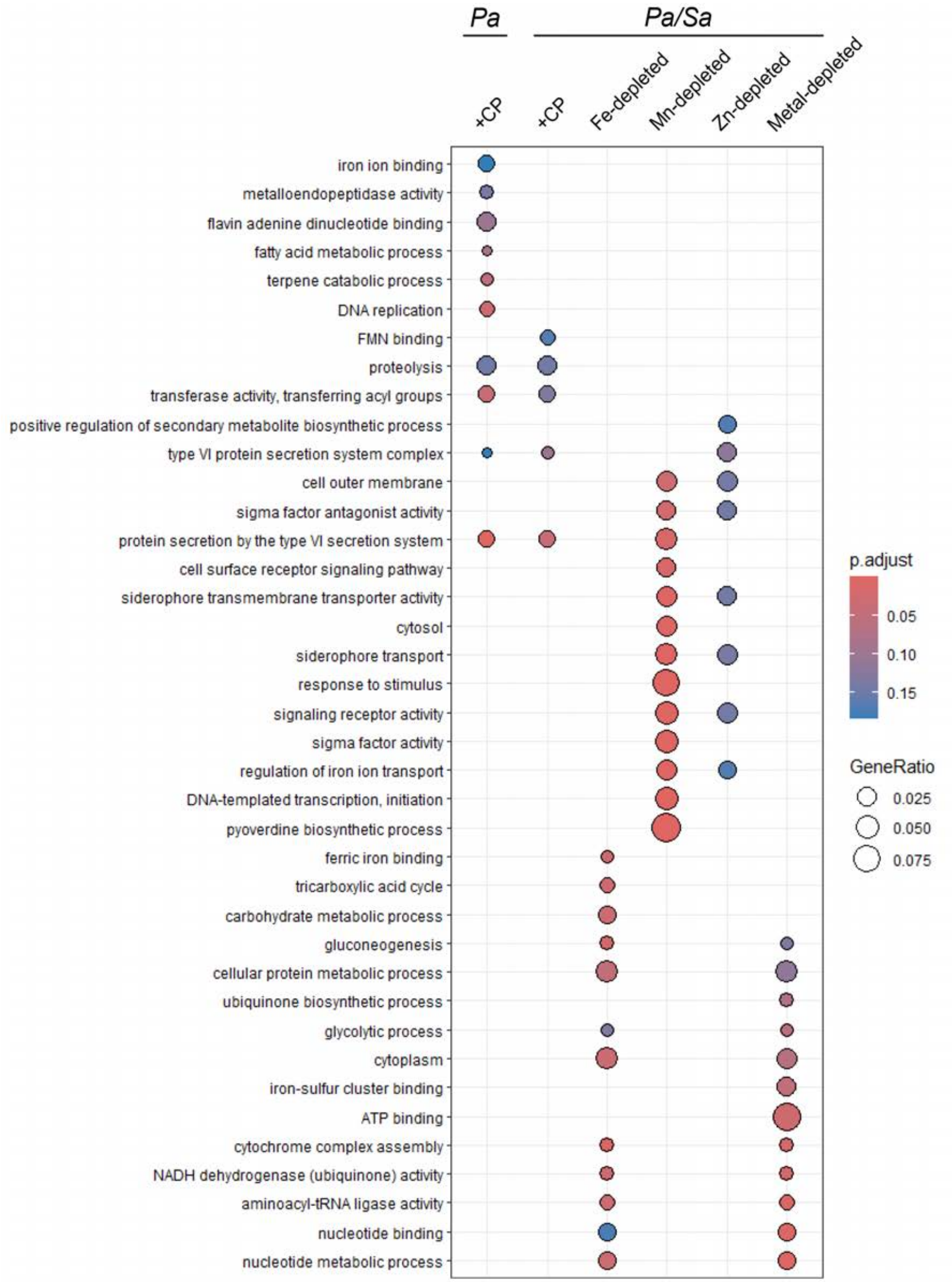

Figure S5D. Overrepresentation analysis of downregulated *P. aeruginosa* genes across conditions. P-values were calculated using the Benjamini-Hochberg method, and the gene ratio represents the proportion of genes out of all genes considered for the tested condition. *Pa* indicates *P. aeruginosa* monoculture and *Pa/Sa* indicates the coculture.

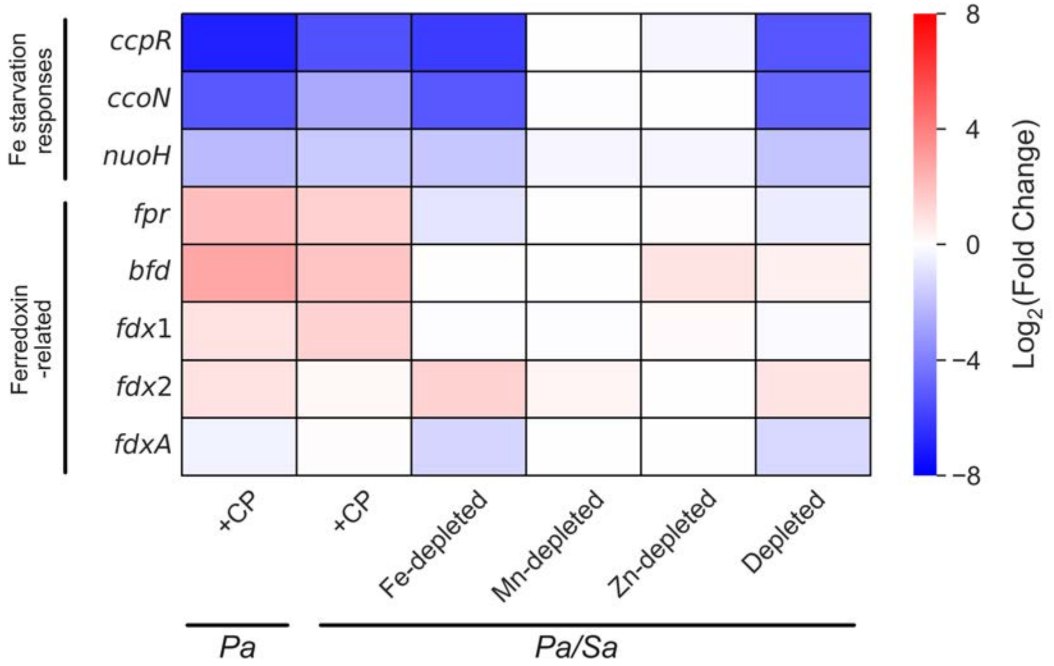

Figure S6. Fe limitation impacts cytochrome preference and respiration in *P. aeruginosa* cocultured with *S. aureus*. Differential expression heatmap of *P. aeruginosa* genes associated with cytochrome preference, respiration, and ferredoxins. *Pa* indicates *P. aeruginosa* monoculture and *Pa/Sa* indicates the coculture.

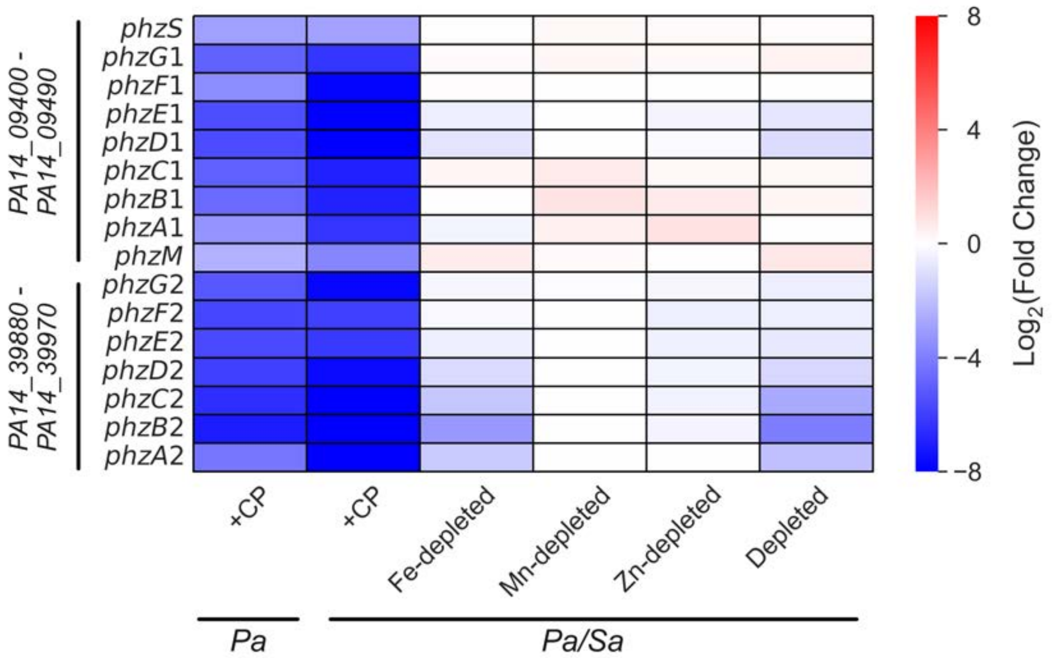

Figure S7. CP decreases the expression of phenazine biosynthetic machinery in *P. aeruginosa* cocultured with *S. aureus*. Differential expression heatmap of *P. aeruginosa* genes in two distinct loci associated with phenazine biosynthesis. *Pa* indicates *P. aeruginosa* monoculture and *Pa/Sa* indicates the coculture.

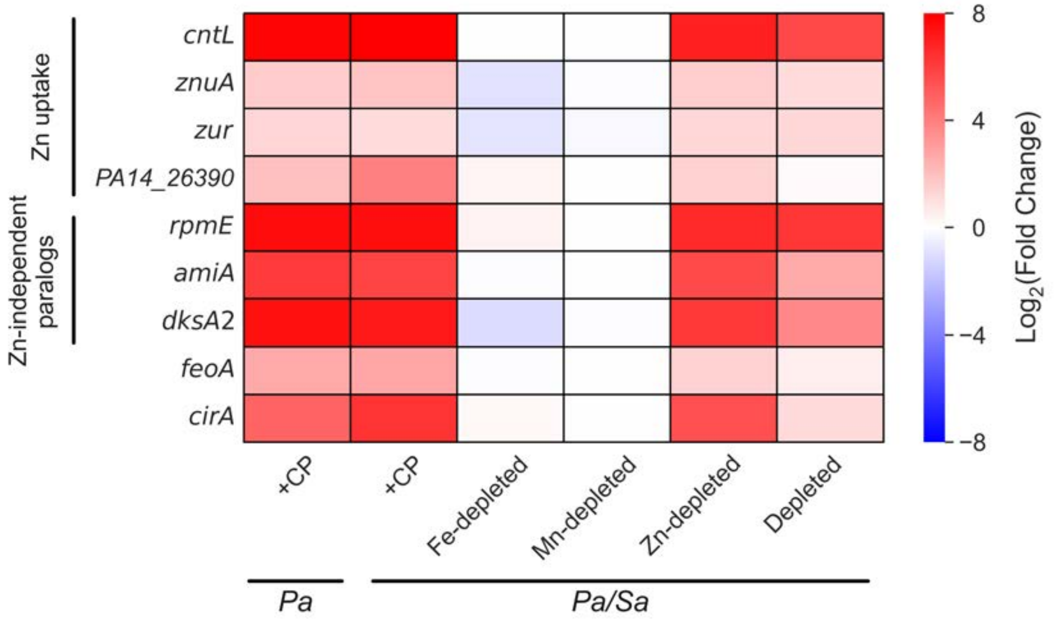

Figure S8. CP elicits Zn-starvation responses for *P. aeruginosa* cocultured with *S. aureus*. Differential expression heatmap of *P. aeruginosa* genes with similar transcriptional responses to CP treatment and Zn depletion. *Pa* indicates *P. aeruginosa* monoculture and *Pa/Sa* indicates the coculture.

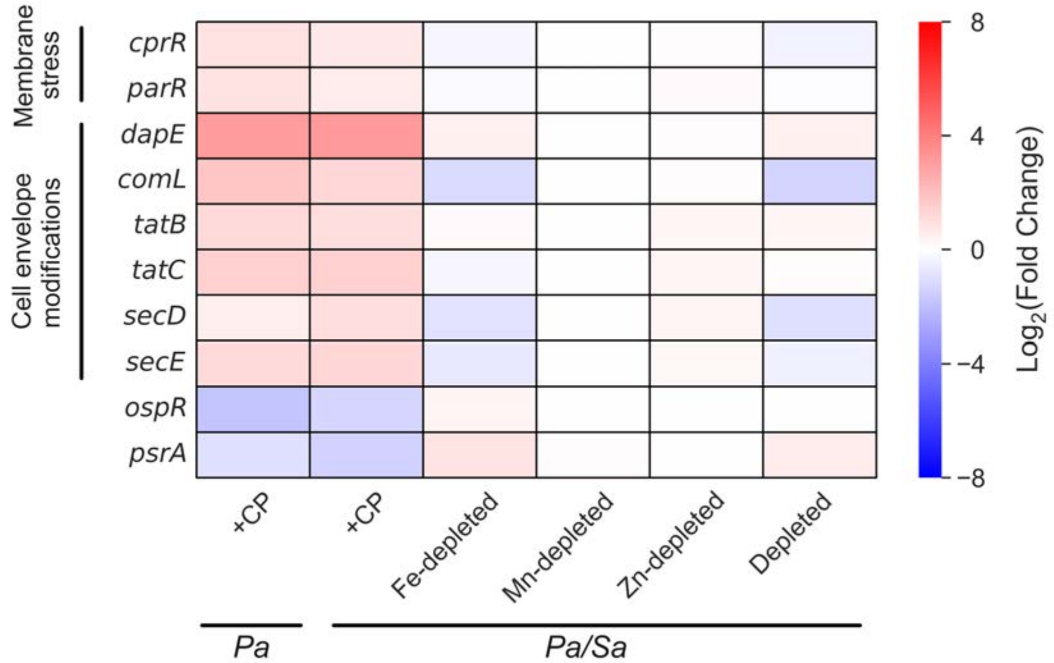

Figure S9. Additional transcriptional responses of *P. aeruginosa* to CP treatment associated with cell envelope modifications. Differential expression heatmap of *P. aeruginosa* genes associated with membrane stress and cell wall modification and other regulators. *Pa* indicates *P. aeruginosa* monoculture and *Pa/Sa* indicates the coculture.

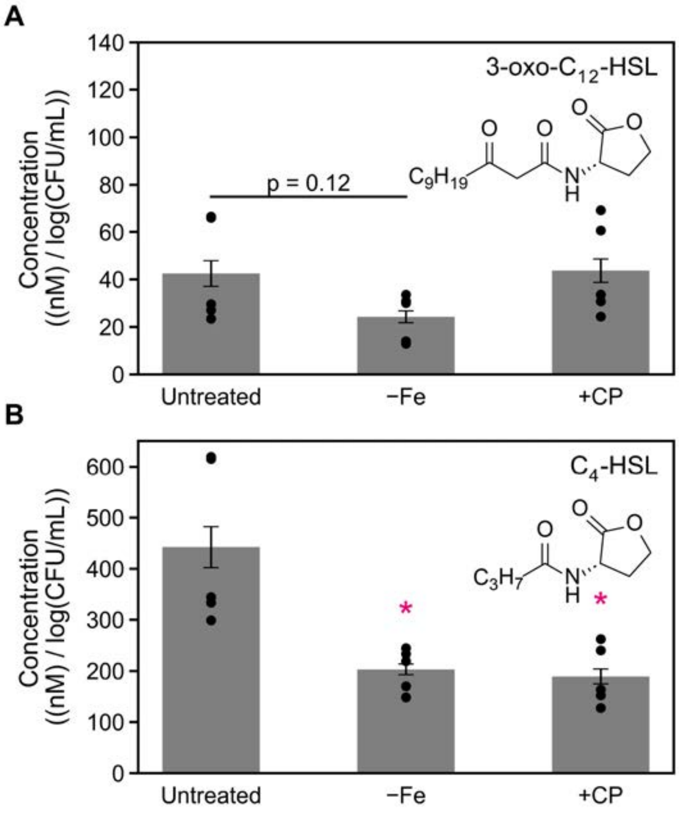

Figure S10. CP treatment and Fe depletion perturb production of the QS autoinducer C_4-HSL in *P. aeruginosa*/*S. aureus* co-cultures. CP treatment does not significantly affect levels of 3-oxo-C_{12}-HSL (**A**) and decreases levels of C_4-HSL (**B**). Aliquots from culture supernatants were collected from cocultures of *P. aeruginosa* and *S. aureus* grown in metal-replete CDM at 37 °C, 11 h, and processed for quantitative mass spectrometry. Metabolite levels were normalized to *P. aeruginosa* CFUs (n=5, error bars represent S.E.). For comparison with the untreated culture condition, * $p < 0.05$.

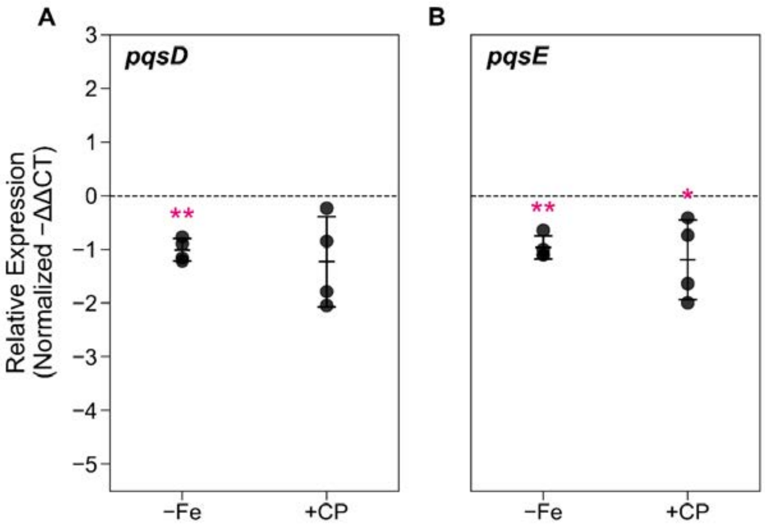

Figure S11. CP treatment and Fe depletion downregulate the expression of genes encoding for PQS biosynthetic machinery in *P. aeruginosa*/*S. aureus* cocultures. CP treatment and Fe depletion decreased the expression of *pqsD* (**A**) and *pqsE* (**B**) in *P. aeruginosa* cocultured with *S. aureus*. Transcript levels were normalized to the *P. aeruginosa* housekeeping gene 16S and the fold change after normalization is presented (n=4, * $p < 0.05$, ** $p < 0.01$, error bars represent S.D.). Cultures were grown in Fe-depleted CDM or metal-replete CDM ± 20 μM CP and incubated at 37 °C for 6 h.

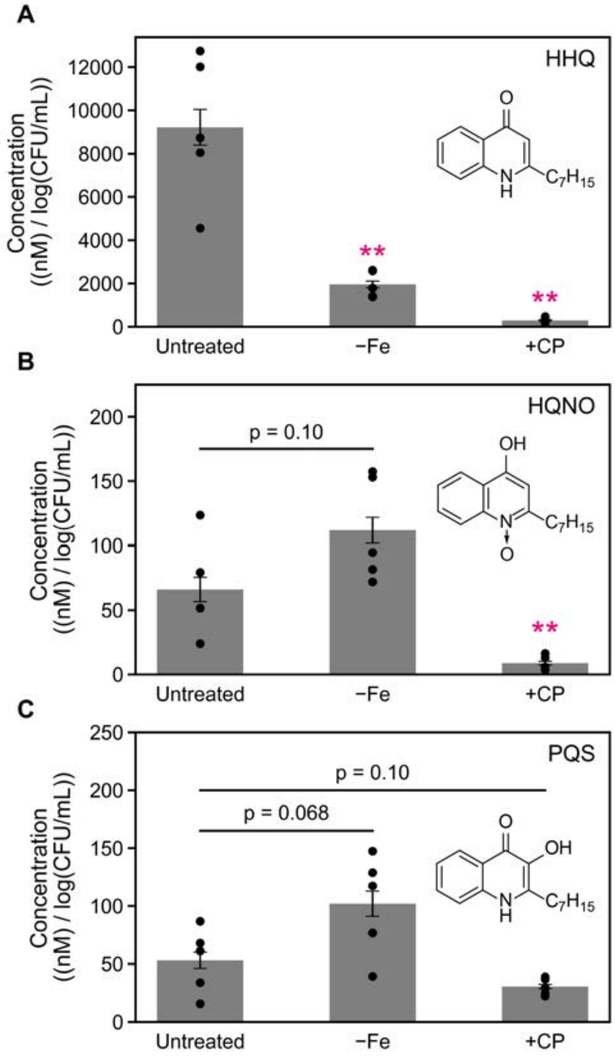

Figure S12. CP treatment decreases the production of C_7 alkylquinolones during early growth in *P. aeruginosa*/*S. aureus* co-cultures. Treatment with CP decreases the levels of HHQ (**A**) and HQNO (**B**), and slightly decreases levels of PQS (**C**). Aliquots from culture supernatants were collected from cocultures of *P. aeruginosa* and *S. aureus* grown in Fe-depleted CDM or metal-replete CDM ± 20 μM CP at 37 °C, 6 h, and processed for quantitative mass spectrometry. Metabolite levels were normalized to *P. aeruginosa* CFUs (n=5, error bars represent S.E.). For comparison with the untreated culture condition, * $p < 0.05$, ** $p < 0.01$.

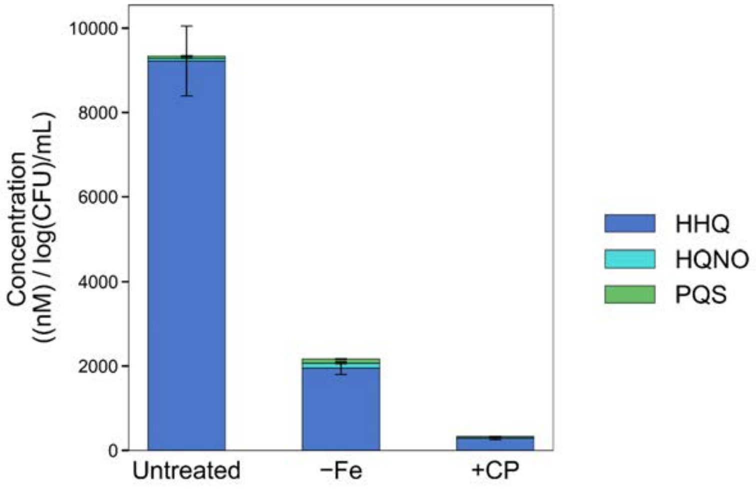

Figure S13. CP treatment and Fe depletion decrease overall levels of C_7 alkylquinolones during early growth in *P. aeruginosa*/*S. aureus* co-cultures. Aliquots from culture supernatants were collected from cocultures of *P. aeruginosa* and *S. aureus* grown in Fe-depleted CDM or metal-replete CDM ± 20 µM CP at 37 °C, 6 h, and processed for quantitative mass spectrometry. Metabolite levels were normalized to *P. aeruginosa* CFUs.

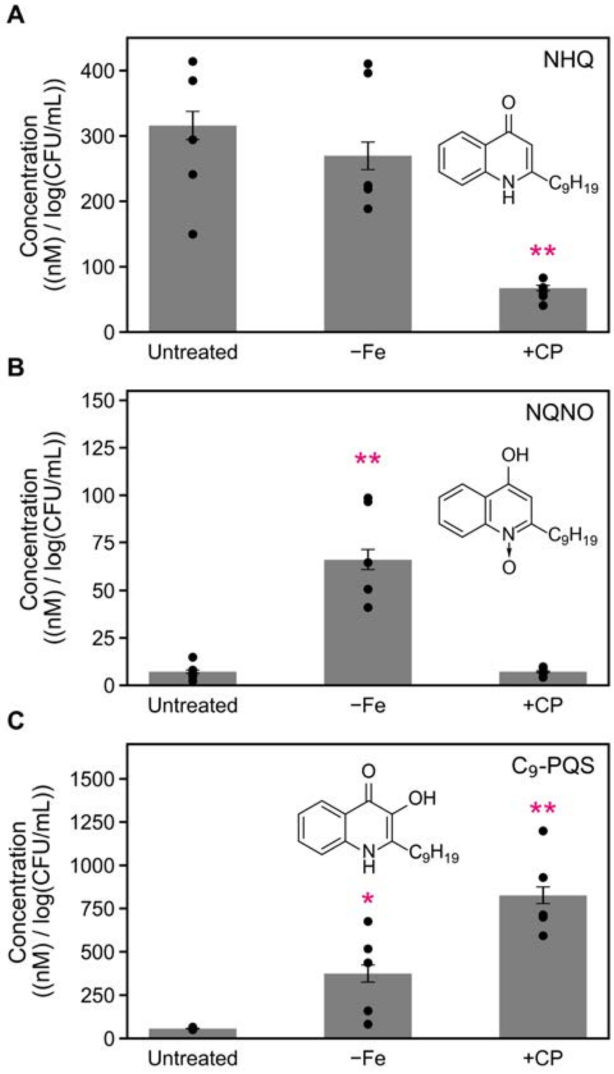

Figure S14. CP treatment decreases levels of the C_9 alkylquinolone NHQ and increases levels of C_9-PQS during early growth in *P. aeruginosa*/*S. aureus* co-cultures. Treatment with CP decreased the levels of NHQ (**A**), but did not have a significant effect on NQNO (**B**) or C_9-PQS (**C**). Aliquots of supernatant were collected from cocultures of *P. aeruginosa* and *S. aureus* grown in Fe-depleted CDM or metal-replete CDM ± 20 μM CP at 37 °C, 6 h, and processed for quantitative mass spectrometry. Metabolite levels were normalized to *P. aeruginosa* CFUs (n=5, error bars represent S.E.). For comparison with the untreated culture condition, ** $p < 0.01$.

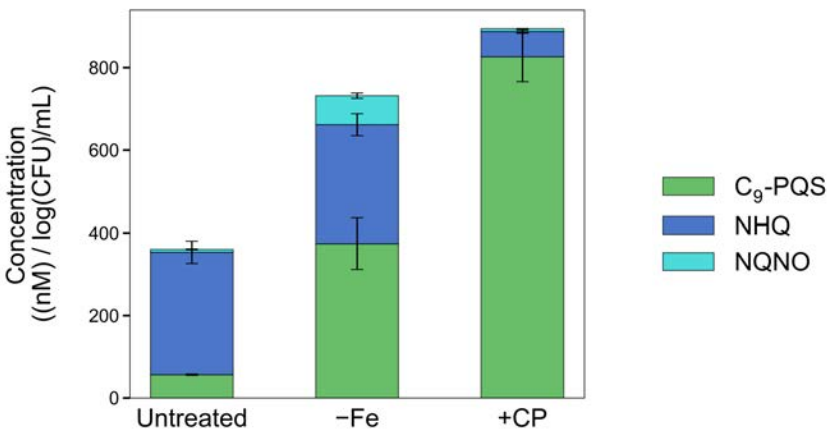

Figure S15. Fe depletion and CP treatment increase overall levels of C_9 alkylquinolones during early growth in *P. aeruginosa*/*S. aureus* co-cultures. Aliquots were collected from cocultures of *P. aeruginosa* and *S. aureus* grown in Fe-depleted CDM or metal-replete CDM ± 20 μM CP at 37 °C, 6 h, and processed for quantification by mass spectrometry. Metabolite levels were normalized to *P. aeruginosa* CFUs.

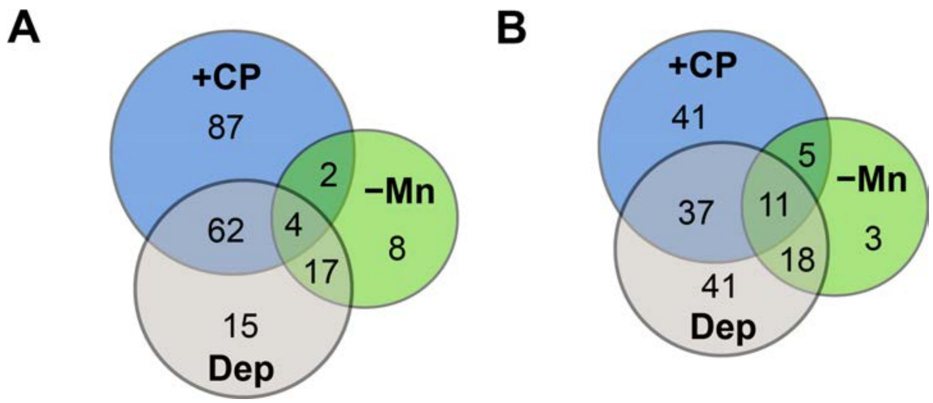

Figure S16. Transcriptional responses of *S. aureus* in coculture with *P. aeruginosa* to Mn depletion differ from responses to CP treatment. Venn diagrams of the top 600 differentially expressed genes across all conditions tested reveals only minor overlap in both upregulated (**A**) genes and downregulated (**B**) genes for CP-treated and Mn-depleted cocultures, whereas moderate overlap was found between CP-treated and metal-depleted (Dep) cocultures.

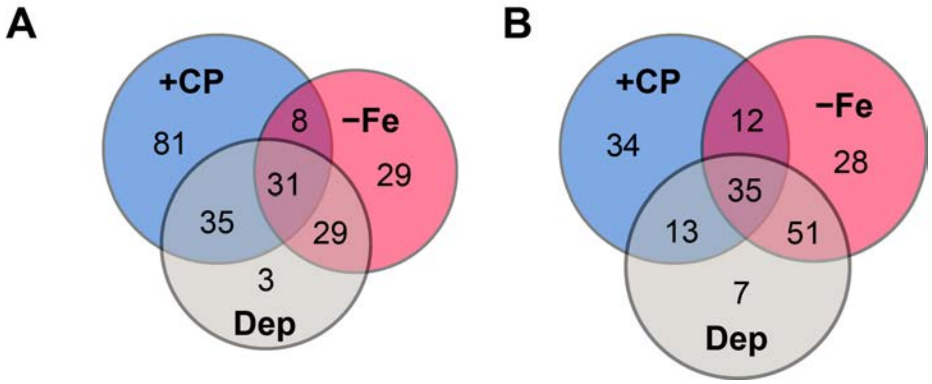

Figure S17. Transcriptional responses of *S. aureus* in coculture with *P. aeruginosa* to Fe depletion overlap with responses to CP treatment. Venn diagrams of the top 600 differentially expressed genes across all conditions tested reveals moderate overlap in both upregulated (**A**) genes and downregulated (**B**) genes for CP-treated, Fe-depleted and metal-depleted (Dep) cocultures.

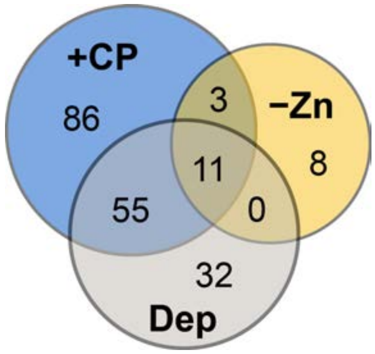

Figure S18. Transcriptional responses of *S. aureus* in coculture with *P. aeruginosa* to Zn depletion are distinct from responses to CP treatment. Venn diagrams of the top 600 differentially expressed genes across all conditions tested reveals moderate overlap for upregulated genes in CP-treated and metal-depleted (Dep) cocultures and only minimal overlap between CP-treated and Zn-depleted cocultures. Zn depletion did not result in the detection of any downregulated genes meeting the significance and DE threshold cutoffs.

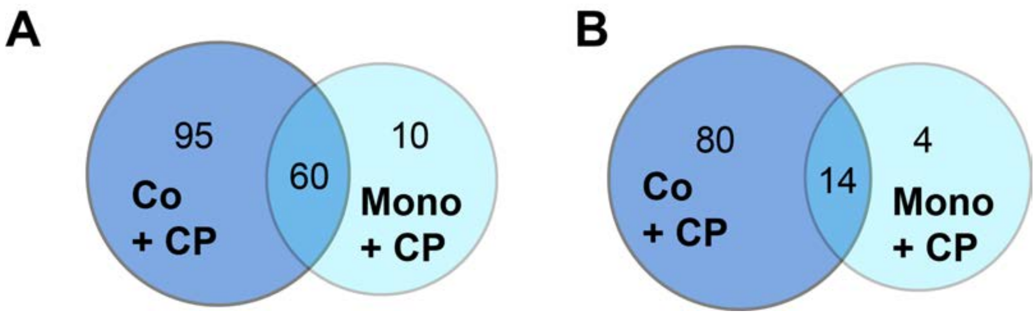

Figure S19. Transcriptional responses of *S. aureus* in coculture with *P. aeruginosa* to CP treatment differ significantly from responses of *S. aureus* in monoculture. Venn diagrams of the top 600 differentially expressed genes across all conditions tested reveals only partial overlap of upregulated (**A**) and downregulated (**B**) genes.

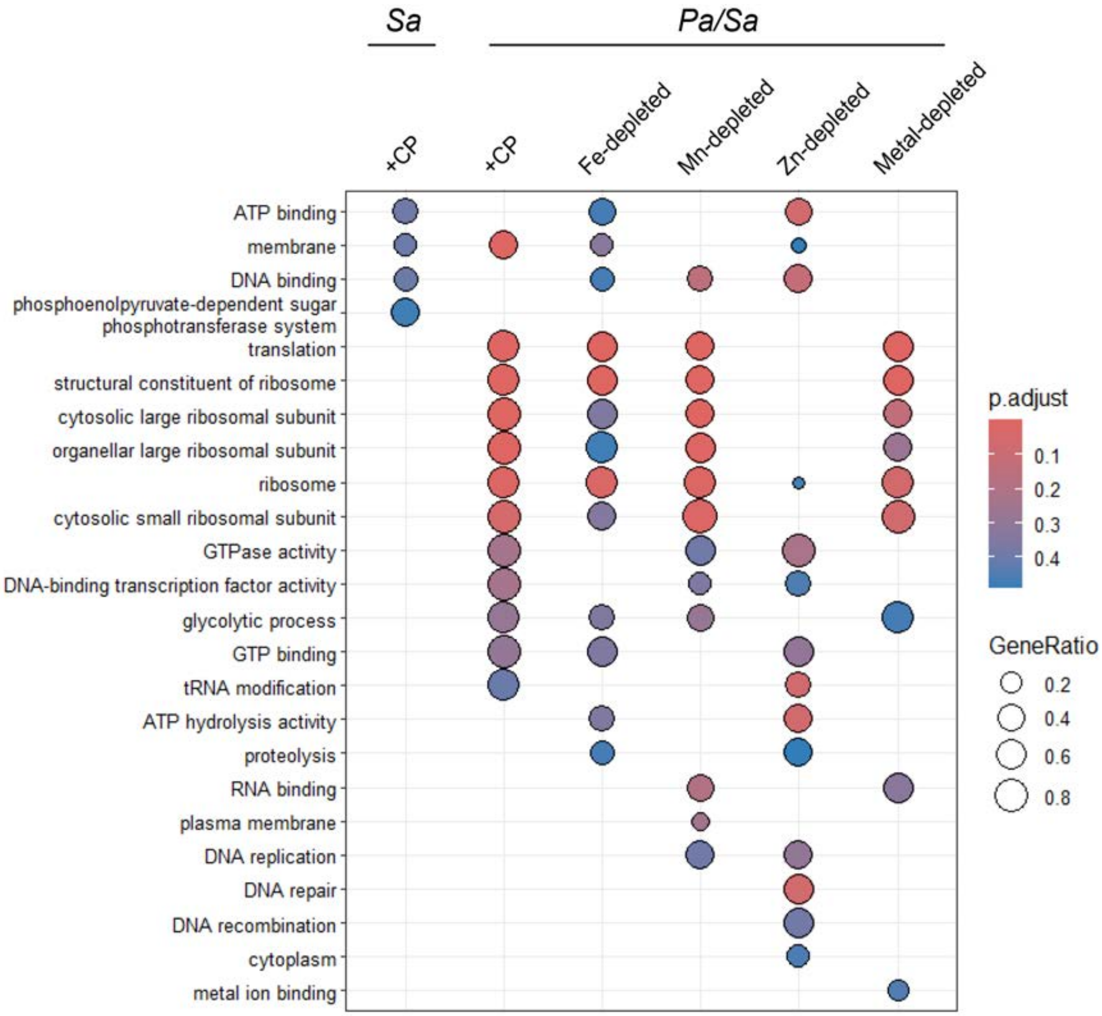

Figure S20. Gene set enrichment analysis of upregulated and downregulated *S. aureus* genes across conditions. P-values were calculated using the Benjamini-Hochberg method, and the gene ratio represents the proportion of genes out of all genes considered for the tested condition. *Sa* indicates *S. aureus* monoculture and *Pa/Sa* indicates the coculture.

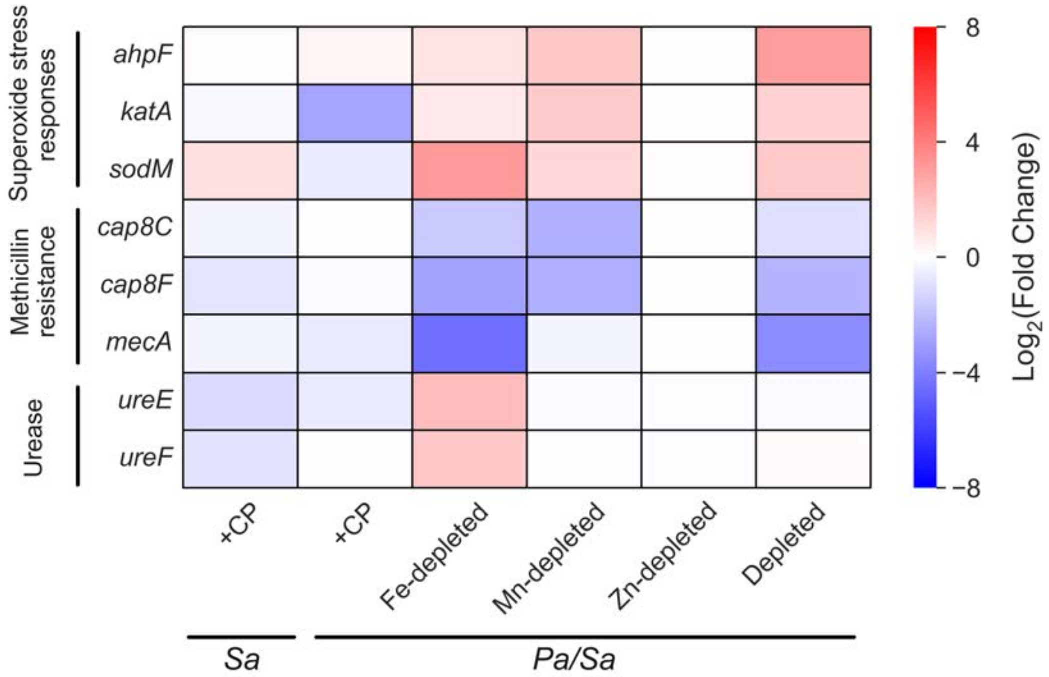

Figure S21. Fe depletion upregulates superoxide stress responses, decreases methicillin resistance and increases the expression of urease genes in *S. aureus* cocultured with *P. aeruginosa*. Differential expression heatmap of *S. aureus* genes associated with superoxide stress responses, methicillin resistance, and urease. *Sa* indicates *S. aureus* monoculture and *Pa/Sa* indicates the coculture.

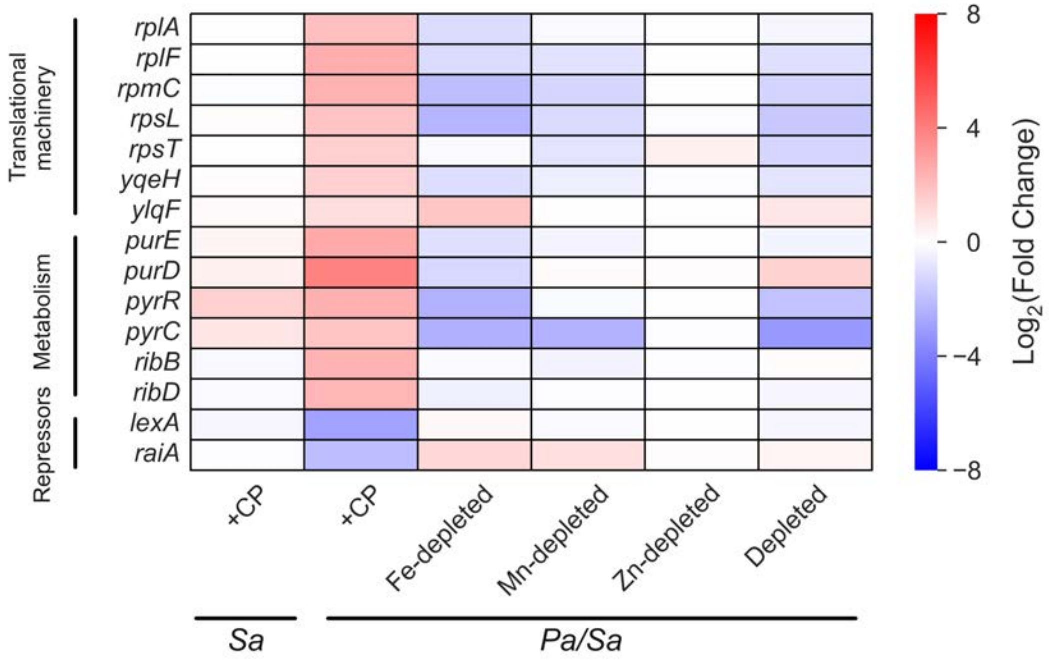

Figure S22. CP treatment increases the expression of genes associated with translational activity in *S. aureus* cocultured with *P. aeruginosa*. Differential expression heatmap of *S. aureus* genes associated with translational machinery and metabolism. *Sa* indicates *S. aureus* monoculture and *Pa/Sa* indicates the coculture.

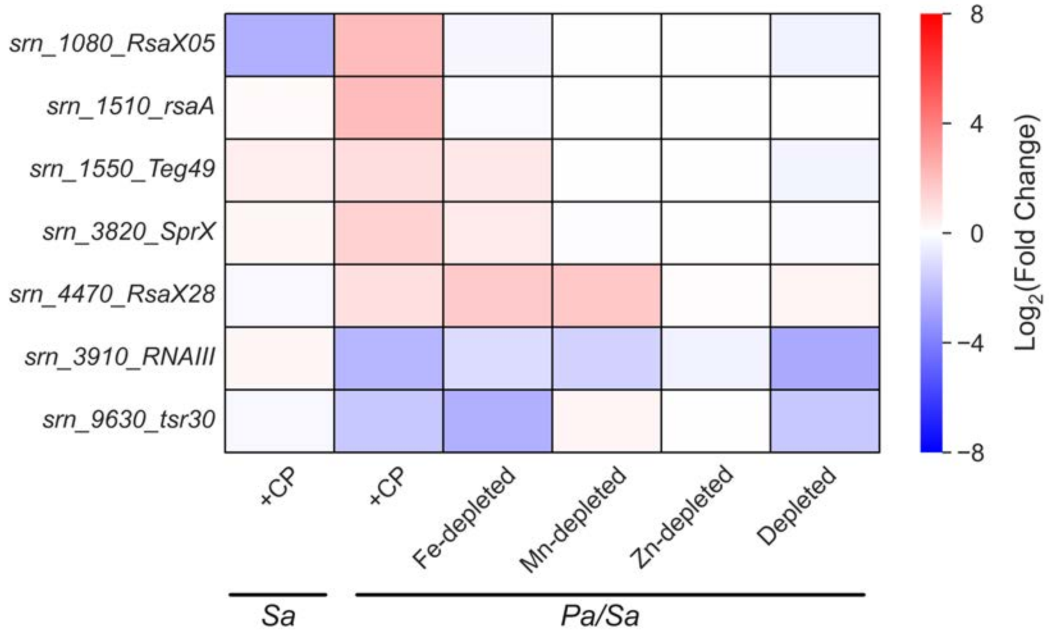

Figure S23. Differential expression heatmap of selected *S. aureus* regulatory RNAs (85). For the complete list of *S. aureus* regulatory RNAs examined, please see **Table SF21**. *Sa* indicates *S. aureus* monoculture and *Pa/Sa* indicates the coculture.

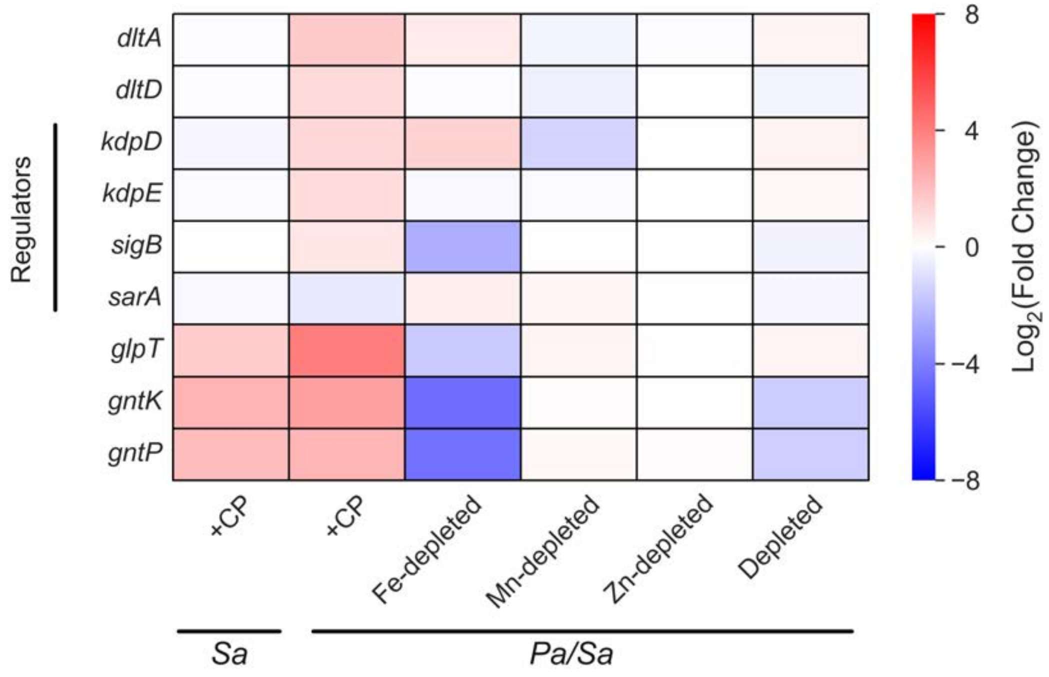

Figure S24. Differential expression heatmap of other unique responses of *S. aureus* cocultured with *P. aeruginosa* to CP treatment. *Sa* indicates *S. aureus* monoculture and *Pa/Sa* indicates the coculture.

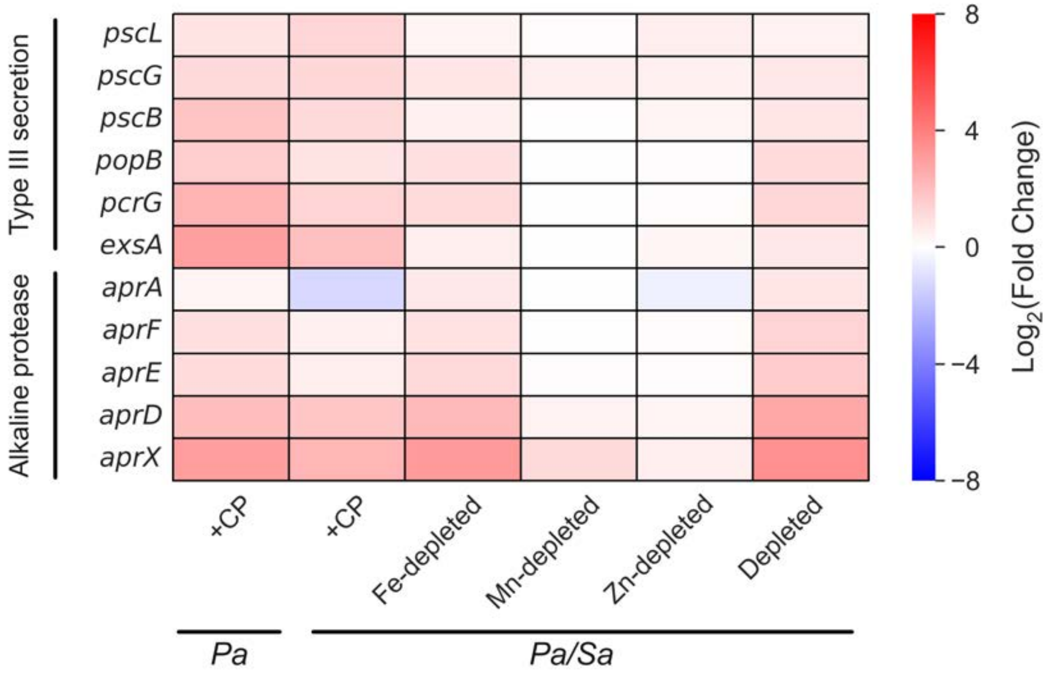

Figure S25. CP treatment and Fe depletion increase the expression of *P. aeruginosa* genes involved in type III secretion systems and the production and transport of alkaline protease. Differential expression heatmap of *P. aeruginosa* associated with type III secretion and alkaline protease. *Pa* indicates *P. aeruginosa* monoculture and *Pa/Sa* indicates the coculture.

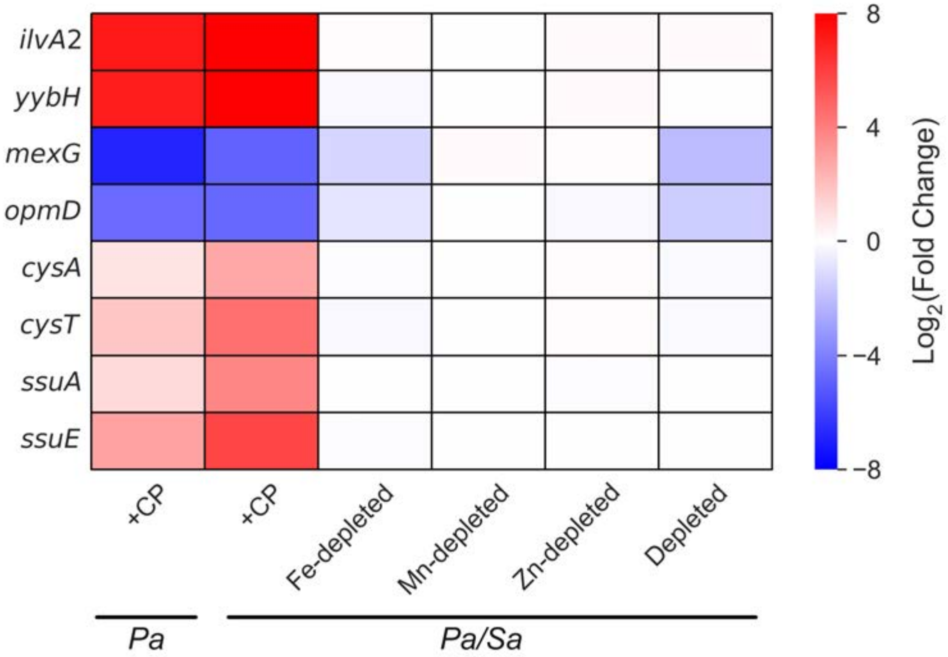

Figure S26. CP treatment affects the expression of *P. aeruginosa* genes perturbed by the P443L mutation of the elongation factor mutant FusA1 in *P. aeruginosa* PAO1. Differential expression heatmap of genes perturbed by the FusA1^{P443L} mutation (28). *Pa* indicates *P. aeruginosa* monoculture and *Pa/Sa* indicates the coculture.

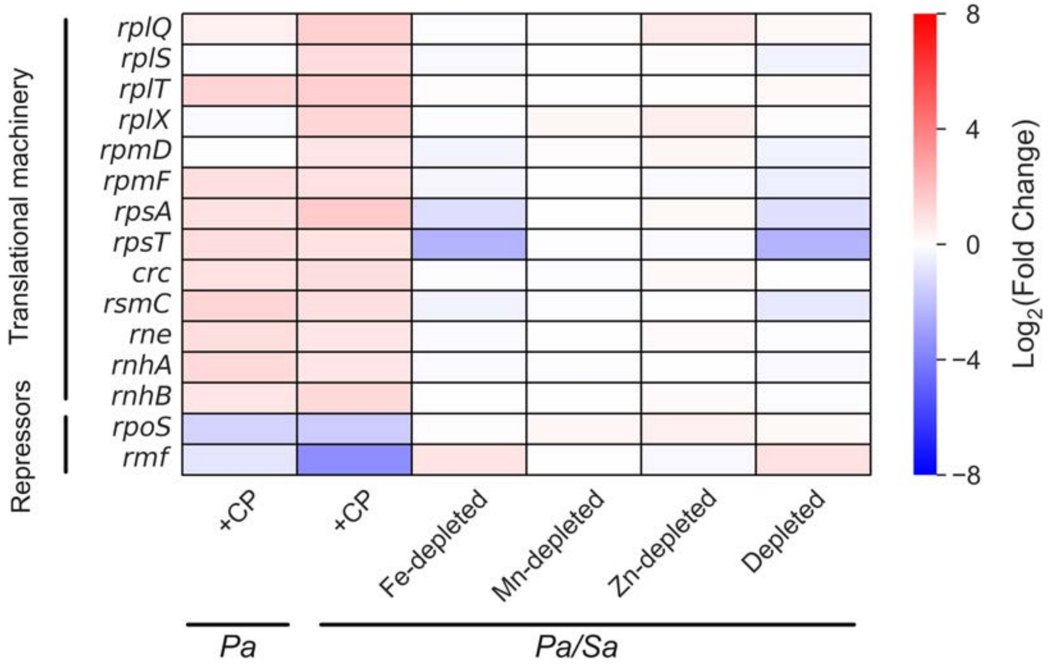

Figure S27. CP treatment affects the expression of translational machinery in *P. aeruginosa*. Differential expression heatmap of genes associated with translational machinery and regulation of translational activity. *Pa* indicates *P. aeruginosa* monoculture and *Pa/Sa* indicates the coculture.

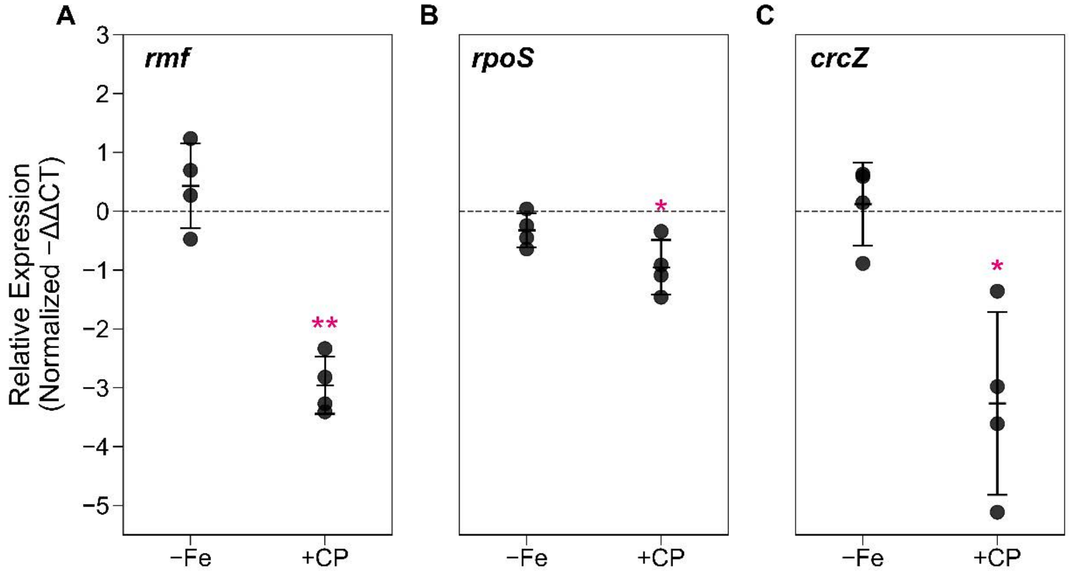

Figure S28. CP treatment increases translational activity and catabolite repression in *P. aeruginosa* cocultured with *S. aureus*. Real-time PCR demonstrates that CP downregulates the expression of the ribosome modulation factor *rmf* (**A**), the stationary phase sigma factor *rpoS* (**B**), and the positive sRNA regulator of catabolite repression *crcZ* (**C**). Cultures were grown in Fe-depleted CDM or metal-replete CDM ± 20 μM CP and incubated at 37 °C for 6 h. Transcript levels were normalized to the *P. aeruginosa* housekeeping gene 16S and the fold change after normalization is presented (n=4, * $p < 0.05$, ** $p < 0.01$, error bars represent S.D.).

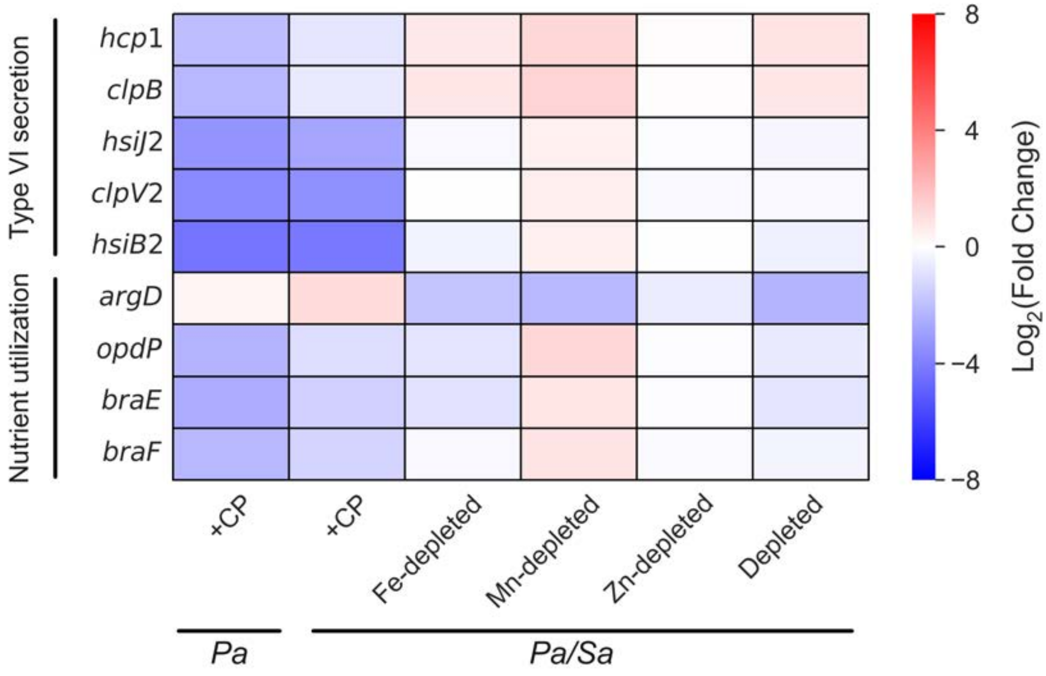

Figure S29. Mn depletion increases the expression of genes involved in type VI secretion and alters nutrient preference in *P. aeruginosa* cocultured with *S. aureus*. Differential expression heatmap of genes associated with type VI secretion and nutrient utilization. *Pa* indicates *P. aeruginosa* monoculture and *Pa/Sa* indicates the coculture.

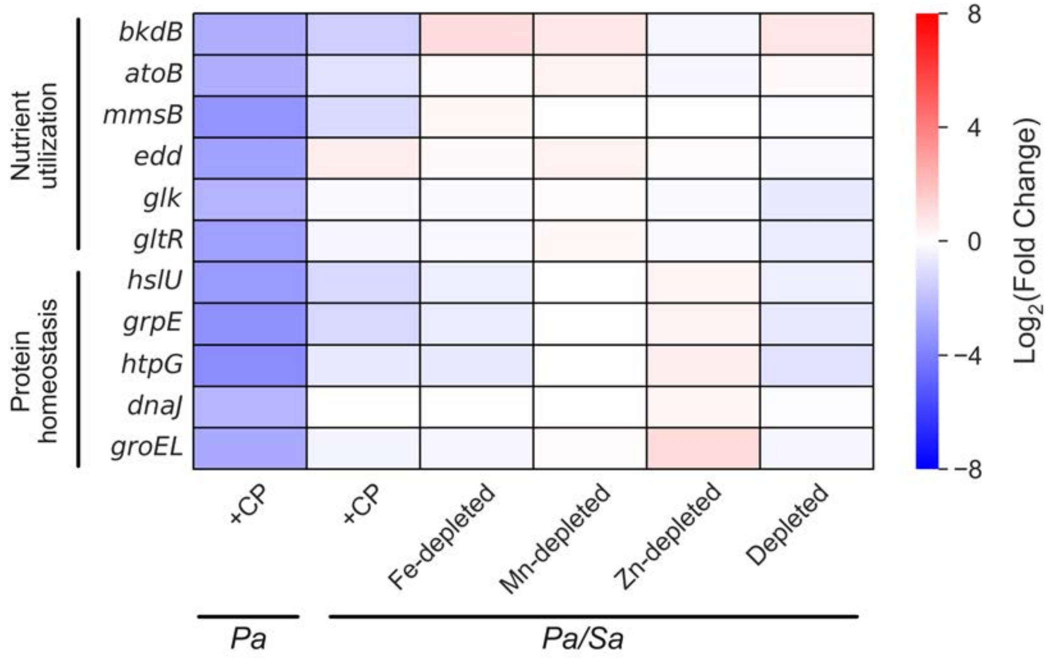

Figure S30. Differential expression heatmap of other unique responses of *P. aeruginosa* monocultures to CP treatment. *Pa* indicates *P. aeruginosa* monoculture and *Pa/Sa* indicates the coculture.

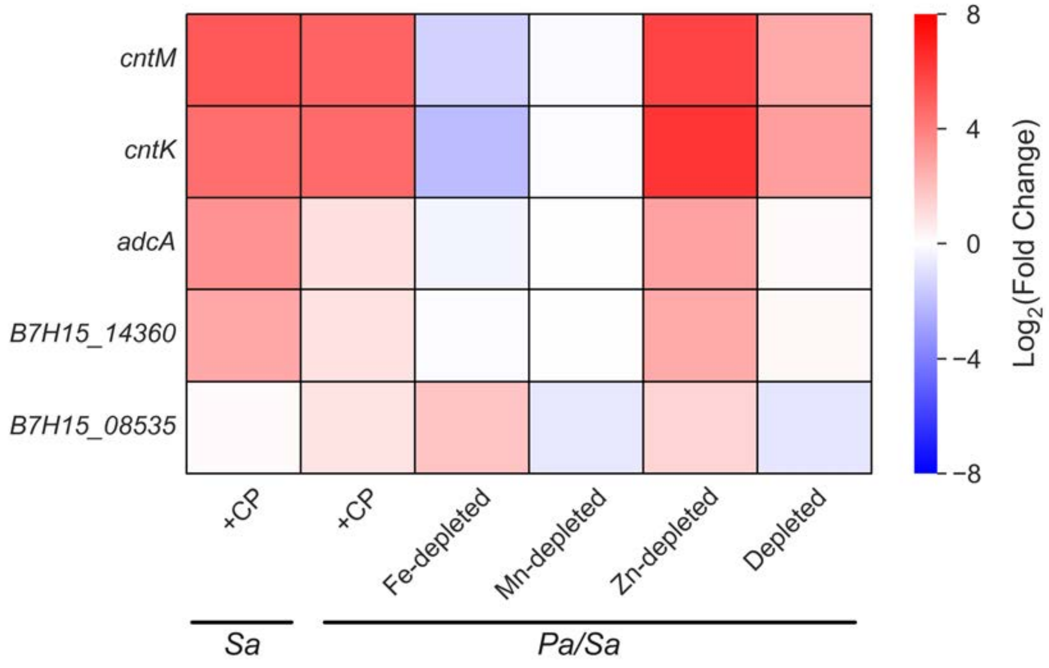

Figure S31. CP increases the expression of genes associated with Zn uptake in *S. aureus*. Differential expression heatmap of *S. aureus* genes associated with Zn uptake machinery. *Sa* indicates *S. aureus* monoculture and *Pa/Sa* indicates the coculture.

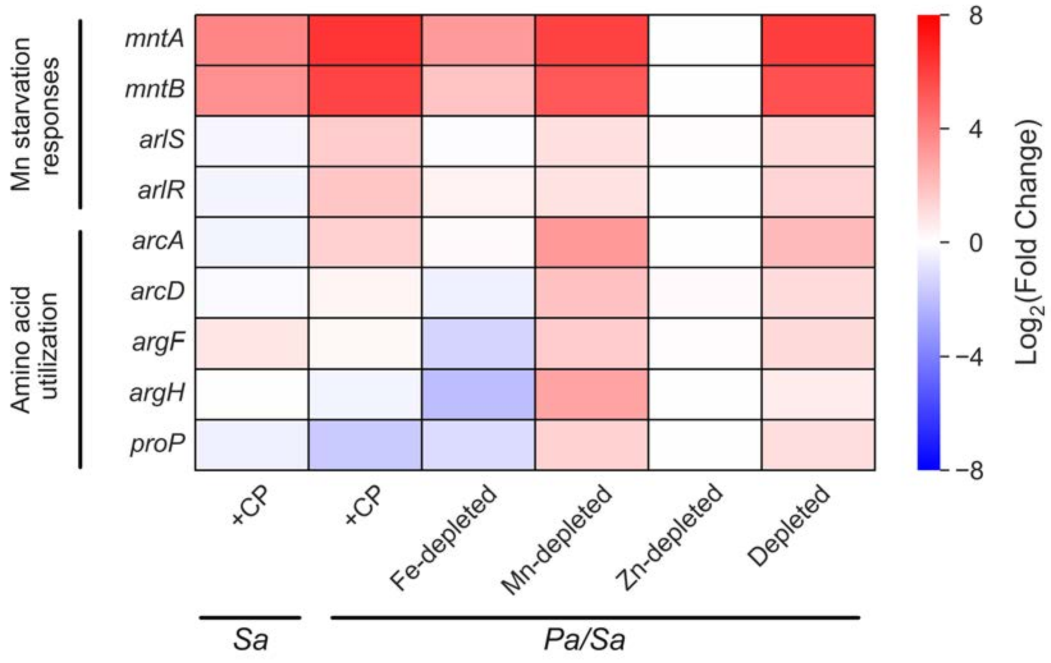

Figure S32. CP elicits Mn-starvation responses from *S. aureus* cocultured with *P. aeruginosa*. Differential expression heatmap of genes associated with Mn-starvation responses and amino acid utilization. *Sa* indicates *S. aureus* monoculture and *Pa/Sa* indicates the coculture.

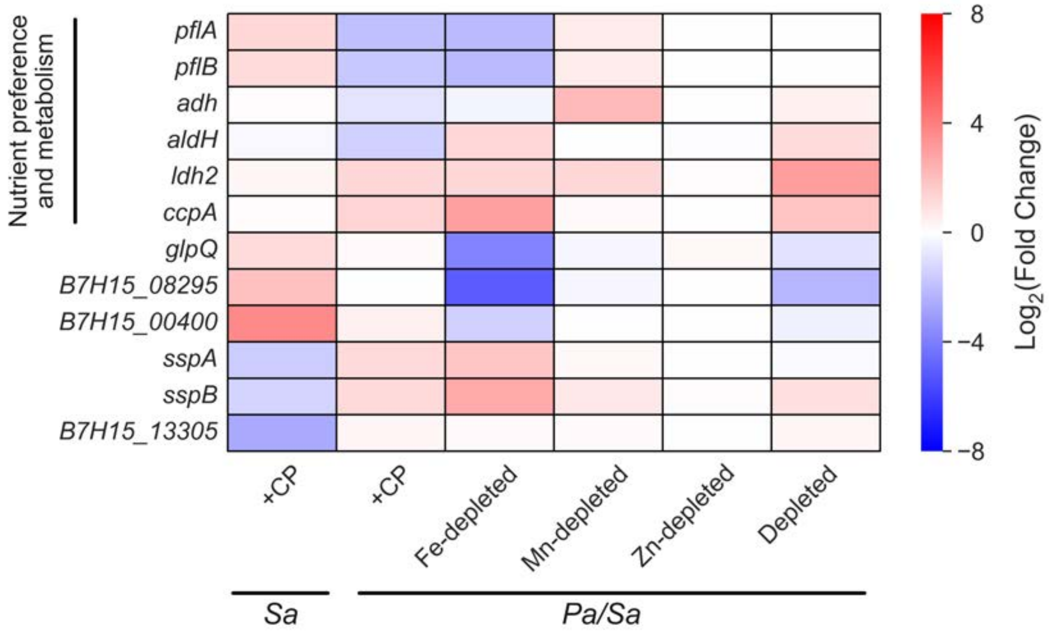

Figure S33. Differential expression heatmap of other unique responses of *S. aureus* monocultures to CP treatment. *Sa* indicates *S. aureus* monoculture and *Pa/Sa* indicates the coculture.

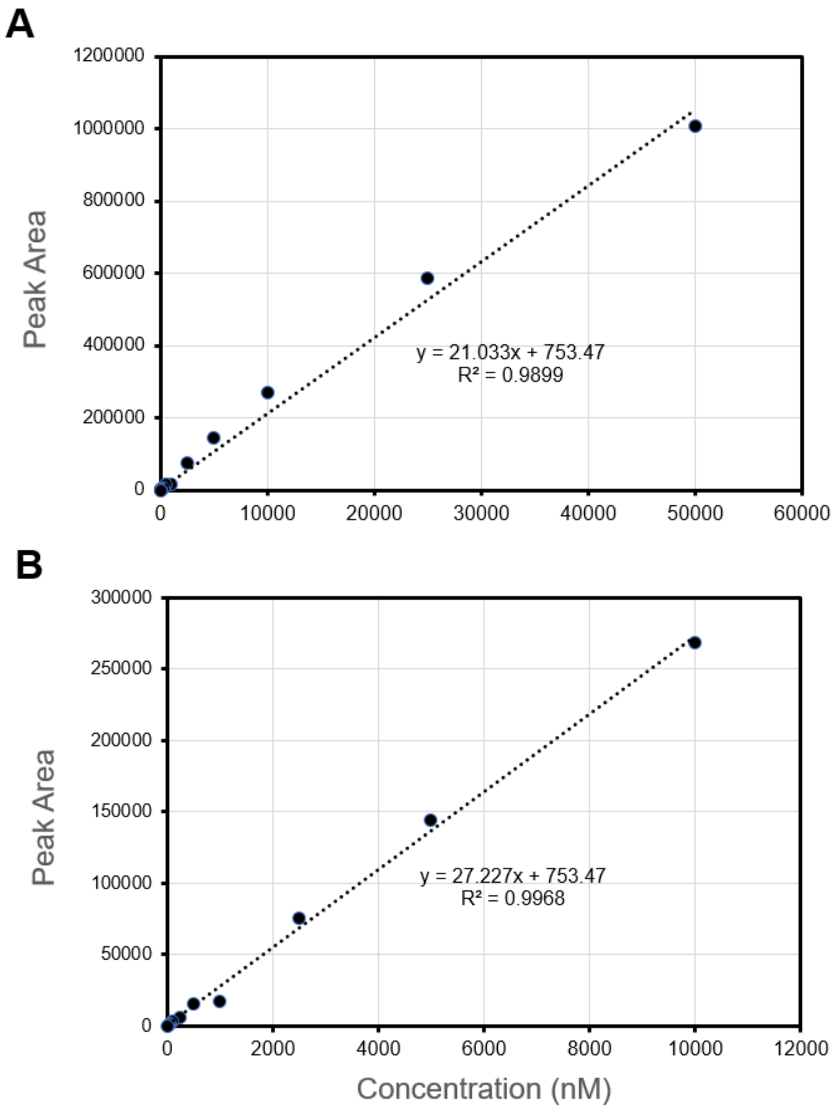

Figure S34. Determination of effective range for quantification of C_6-HSL-d_3 using triple quadrupole mass spectrometry. Peak area was quantified over the full window of assayed concentrations, 10 nM – 50 µM (**A**) and 10 nM – 10 µM (**B**).

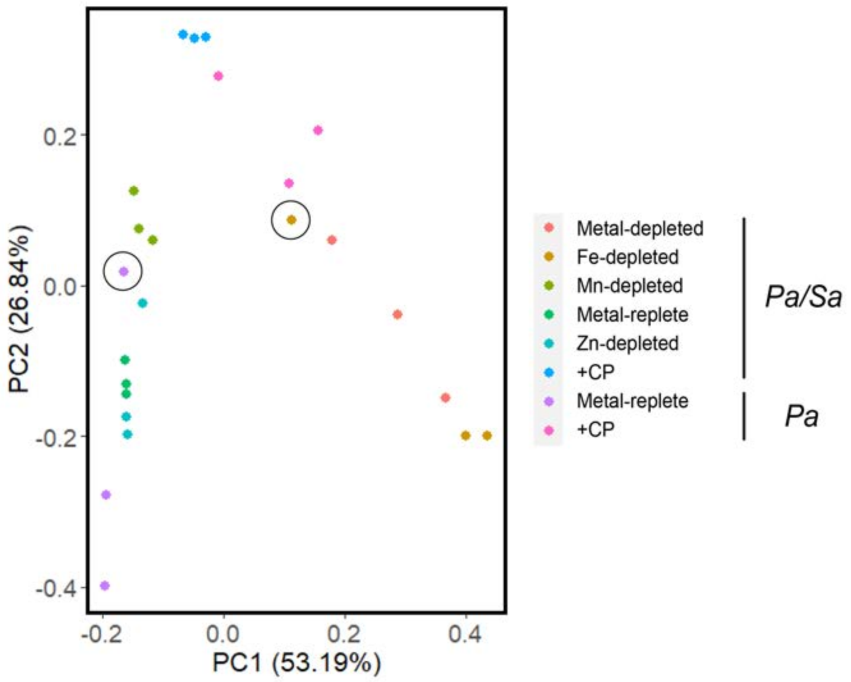

Figure S35. Principal component analysis of *P. aeruginosa* libraries. Two libraries of smaller size (black circles) clustered far away from other biological replicates of the same condition.

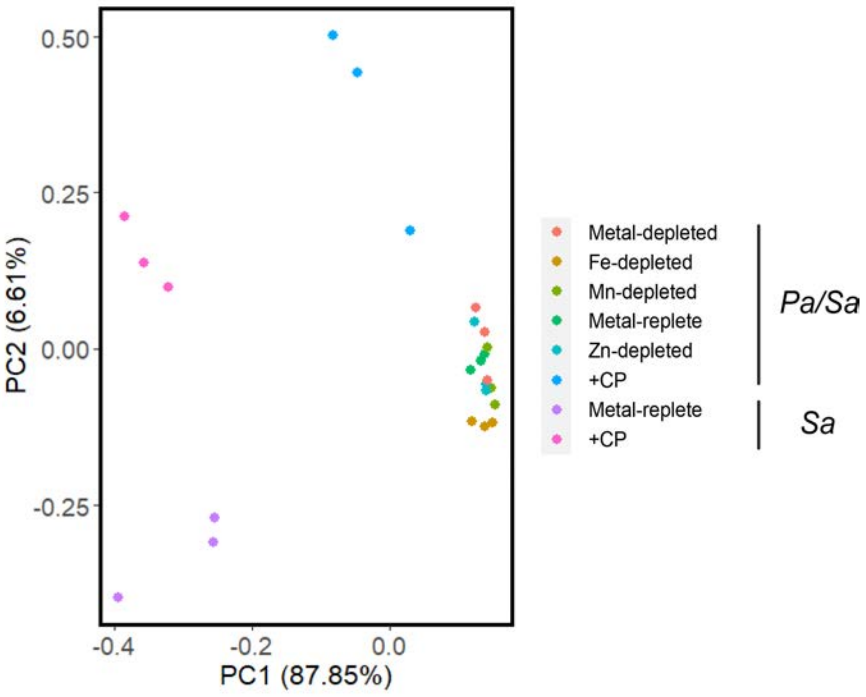

Figure S36. Principal component analysis of *S. aureus* libraries.

SUPPLEMENTARY REFERENCES

1. Lee WH, Zygiel EM, Lee, CH, Oglesby AG, Nolan EM. 2025. Calprotectin-mediated survival of *Staphylococcus aureus* in coculture with *Pseudomonas aeruginosa* occurs without nutrient metal sequestration. mBio 16:e084624.

2. Brophy MB, Hayden JA, Nolan EM. 2012. Calcium ion gradients modulate the zinc affinity and antibacterial activity of human calprotectin. J Am Chem Soc 134:18089–18100.

3. Nakashige TG, Zygiel EM, Drennan CL, Nolan EM. 2017. Nickel sequestration by the host-defense protein human calprotectin. J Am Chem Soc 139:8828–8836.

4. Rahme LG, Stevens EJ, Wolfort SF, Shao J, Tompkins RG, Ausubel FM. 1995. Common virulence factors for bacterial pathogenicity in plants and animals. Science 268:1899–1902.

5. Fey PD, Endres JL, Yajjala VK, Widhelm TJ, Boissy RJ, Bose JL, Bayles KW. 2013. A genetic resource for rapid and comprehensive phenotype screening of nonessential *Staphylococcus aureus* genes. mBio 4:e00537-12.

6. Kim D, Paggi JM, Park C, Bennett C, Salzberg SL. 2019. Graph-based genome alignment and genotyping with HISAT2 and HISAT-genotype. Nat Biotechnol 37:907–915.

7. Lee DG, Urbach JM, Wu G, Liberati NT, Feinbaum RL, Miyata S, Diggins LT, He J, Saucier M, Déziel E, Friedman L, Li L, Grills G, Montgomery K, Kucherlapati R, Rahme LG, Ausubel FM. 2006. Genomic analysis reveals that *Pseudomonas aeruginosa* virulence is combinatorial. Genome Biol 7:R90.

8. Chung M, Adkins RS, Mattick JSA, Bradwell KR, Shetty AC, Sadzewicz L, Tallon LJ, Fraser CM, Rasko DA, Mahurkar A, Dunning Hotopp JC. 2021. FADU: a Quantification Tool for Prokaryotic Transcriptomic Analyses. mSystems 6:e00917-20.

9. Dixon P. 2003. VEGAN, a package of R functions for community ecology. J Veg Sci 14:927–930.

10. Sassi M, Augagneur Y, Mauro T, Ivain L, Chabelskaya S, Hallier M, Sallou O, Felden B. 2015. SRD: a *Staphylococcus* regulatory RNA database. RNA 21:1005–1017.

11. Li W, Godzik A. 2006. Cd-hit: a fast program for clustering and comparing large sets of protein or nucleotide sequences. Bioinformatics 22:1658–1659.

12. Love MI, Huber W, Anders S. 2014. Moderated estimation of fold change and dispersion for RNA-seq data with DESeq2. Genome Biol 15:550.

13. Zhu A, Ibrahim JG, Love MI. 2019. Heavy-tailed prior distributions for sequence count data: removing the noise and preserving large differences. Bioinformatics 35:2084–2092.

14. Anders S, Huber W. 2010. Differential expression analysis for sequence count data. Genome Biol 11:R106.

15. Yu G, Wang L-G, Han Y, He Q-Y. 2012. clusterProfiler: an R package for comparing biological themes among gene clusters. OMICS 16:284–287.

16. Wu T, Hu E, Xu S, Chen M, Guo P, Dai Z, Feng T, Zhou L, Tang W, Zhan L, Fu X, Liu S, Bo X, Yu G. 2021. clusterProfiler 4.0: A universal enrichment tool for interpreting omics data. Innovation (Camb) 2:100141.

17. Winsor GL, Griffiths EJ, Lo R, Dhillon BK, Shay JA, Brinkman FSL. 2016. Enhanced annotations and features for comparing thousands of *Pseudomonas* genomes in the *Pseudomonas* genome database. Nucleic Acids Res 44:D646–D653.

18. Fuchs S, Mehlan H, Bernhardt J, Hennig A, Michalik S, Surmann K, Pané-Farré J, Giese A, Weiss S, Backert L, Herbig A, Nieselt K, Hecker M, Völker U, Mäder U. 2018. *Aureo*Wiki‑The repository of the *Staphylococcus aureus* research and annotation community. Int J Med Microbiol 308:558–568.

19. Ellfolk N, Rönnberg M, Osterlund K. 1991. Structural and functional features of *Pseudomonas* cytochrome c peroxidase. Biochim Biophys Acta 1080:68–77.

20. Romsang A, Duang-nkern J, Wirathorn W, Vattanaviboon P, Mongkolsuk S. 2015. *Pseudomonas aeruginosa* IscR-Regulated Ferredoxin NADP(+) Reductase Gene (*fprB*) Functions in Iron-Sulfur Cluster Biogenesis and Multiple Stress Response. PLoS ONE 10:e0134374.

21. Yeom J, Imlay JA, Park W. 2010. Iron Homeostasis Affects Antibiotic-mediated Cell Death in *Pseudomonas* Species. J Biol Chem 285:22689.

22. Yeom S, Yeom J, Park W. 2010. Molecular characterization of FinR, a novel redox-sensing transcriptional regulator in *Pseudomonas putida* KT2440. Microbiology (Reading) 156:1487–1496.

23. Pletzer D, Lafon C, Braun Y, Köhler T, Page MGP, Mourez M, Weingart H. 2014. High-throughput screening of dipeptide utilization mediated by the ABC transporter DppBCDF and its substrate-binding proteins DppA1-A5 in *Pseudomonas aeruginosa*. PLoS One 9:e111311.

24. Horna G, Ruiz J. 2021. Type 3 secretion system of *Pseudomonas aeruginosa*. Microbiol Res 246:126719.

25. Guzzo J, Pages JM, Duong F, Lazdunski A, Murgier M. 1991. *Pseudomonas aeruginosa* alkaline protease: evidence for secretion genes and study of secretion mechanism. J Bacteriol 173:5290–5297.

26. Okuda K, Morihara K, Atsumi Y, Takeuchi H, Kawamoto S, Kawasaki H, Suzuki K, Fukushima J. 1990. Complete nucleotide sequence of the structural gene for alkaline proteinase from *Pseudomonas aeruginosa* IFO 3455. Infect Immun 58:4083–4088.

27. Frank DW, Iglewski BH. 1991. Cloning and sequence analysis of a trans-regulatory locus required for exoenzyme S synthesis in *Pseudomonas aeruginosa*. J Bacteriol 173:6460–6468.

28. Maunders EA, Triniman RC, Western J, Rahman T, Welch M. 2020. Global reprogramming of virulence and antibiotic resistance in *Pseudomonas aeruginosa* by a single nucleotide polymorphism in elongation factor, *fusA1*. J Biol Chem 295:16411.

29. Aendekerk S, Ghysels B, Cornelis P, Baysse C. 2002. Characterization of a new efflux pump, MexGHI-OpmD, from *Pseudomonas aeruginosa* that confers resistance to vanadium. Microbiology (Reading) 148:2371–2381.

30. Aendekerk S, Diggle SP, Song Z, Høiby N, Cornelis P, Williams P, Cámara M. 2005. The MexGHI-OpmD multidrug efflux pump controls growth, antibiotic susceptibility and virulence in *Pseudomonas aeruginosa* via 4-quinolone-dependent cell-to-cell communication. Microbiology (Reading) 151:1113–1125.

31. Sirko A, Hryniewicz M, Hulanicka D, Böck A. 1990. Sulfate and thiosulfate transport in *Escherichia coli* K-12: nucleotide sequence and expression of the *cysTWAM* gene cluster. J Bacteriol 172:3351–3357.

32. Kahnert A, Vermeij P, Wietek C, James P, Leisinger T, Kertesz MA. 2000. The *ssu* Locus Plays a Key Role in Organosulfur Metabolism in *Pseudomonas putida* S-313. J Bacteriol 182:2869–2878.

33. Hester KL, Lehman J, Najar F, Song L, Roe BA, MacGregor CH, Hager PW, Phibbs PV, Sokatch JR. 2000. Crc Is Involved in Catabolite Repression Control of the bkd Operons of *Pseudomonas putida* and *Pseudomonas aeruginosa*. J Bacteriol 182:1144–1149.

34. Sonnleitner E, Bläsi U. 2014. Regulation of Hfq by the RNA CrcZ in *Pseudomonas aeruginosa* Carbon Catabolite Repression. PLoS Genet 10:e1004440.

35. O'Toole GA, Gibbs KA, Hager PW, Phibbs PV, Kolter R. 2000. The Global Carbon Metabolism Regulator Crc Is a Component of a Signal Transduction Pathway Required for Biofilm Development by *Pseudomonas aeruginosa*. J Bacteriol 182:425–431.

36. Latifi A, Foglino M, Tanaka K, Williams P, Lazdunski A. 1996. A hierarchical quorum-sensing cascade in *Pseudomonas aeruginosa* links the transcriptional activators LasR and RhIR (VsmR) to expression of the stationary-phase sigma factor RpoS. Mol Microbiol 21:1137–1146.

37. Schuster M, Hawkins AC, Harwood CS, Greenberg EP. 2004. The *Pseudomonas aeruginosa* RpoS regulon and its relationship to quorum sensing. Mol Microbiol 51:973–985.

38. Hirsch M, Elliott T. 2002. Role of ppGpp in *rpoS* Stationary-Phase Regulation in *Escherichia coli*. J Bacteriol 184:5077–5087.

39. Sonnleitner E, Abdou L, Haas D. 2009. Small RNA as global regulator of carbon catabolite repression in *Pseudomonas aeruginosa*. Proc Natl Acad Sci USA 106:21866–21871.

40. Wada A, Igarashi K, Yoshimura S, Aimoto S, Ishihama A. 1995. Ribosome modulation factor: stationary growth phase-specific inhibitor of ribosome functions from *Escherichia coli*. Biochem Biophys Res Commun 214:410–417.

41. De Bentzmann S, Giraud C, Bernard CS, Calderon V, Ewald F, Plésiat P, Nguyen C, Grunwald D, Attree I, Jeannot K, Fauvarque M-O, Bordi C. 2012. Unique Biofilm Signature, Drug Susceptibility and Decreased Virulence in *Drosophila* through the *Pseudomonas aeruginosa* Two-Component System PprAB. PLoS Pathog 8:e1003052.

42. Nelson CE, Huang W, Zygiel EM, Nolan EM, Kane MA, Oglesby AG. 2021. The human innate immune protein calprotectin elicits a multimetal starvation response in *Pseudomonas aeruginosa*. Microbiol Spectr 9:e00519-21.

43. Hood RD, Singh P, Hsu F, Güvener T, Carl MA, Trinidad RRS, Silverman JM, Ohlson BB, Hicks KG, Plemel RL, Li M, Schwarz S, Wang WY, Merz AJ, Goodlett DR, Mougous JD. 2010. A Type VI Secretion System of *Pseudomonas aeruginosa* Targets a Toxin to Bacteria. Cell Host Microbe 7:25–37.

44. Sana TG, Hachani A, Bucior I, Soscia C, Garvis S, Termine E, Engel J, Filloux A, Bleves S. 2012. The Second Type VI Secretion System of *Pseudomonas aeruginosa* Strain PAO1 Is Regulated by Quorum Sensing and Fur and Modulates Internalization in Epithelial Cells. J Biol Chem 287:27095–27105.

45. Itoh Y. 1997. Cloning and characterization of the *aru* genes encoding enzymes of the catabolic arginine succinyltransferase pathway in *Pseudomonas aeruginosa*. J Bacteriol 179:7280–7290.

46. Tamber S, Hancock REW. 2006. Involvement of two related porins, OprD and OpdP, in the uptake of arginine by *Pseudomonas aeruginosa*. FEMS Microbiol Lett 260:23–29.

47. Tamber S, Ochs MM, Hancock REW. 2006. Role of the novel OprD family of porins in nutrient uptake in *Pseudomonas aeruginosa*. J Bacteriol 188:45–54.

48. Hoshino T, Kose K. 1990. Cloning, nucleotide sequences, and identification of products of the *Pseudomonas aeruginosa* PAO *bra* genes, which encode the high-affinity branched-chain amino acid transport system. J Bacteriol 172:5531–5539.

49. Tognon M, Köhler T, Luscher A, van Delden C. 2019. Transcriptional profiling of *Pseudomonas aeruginosa* and *Staphylococcus aureus* during *in vitro* co-culture. BMC Genomics 20:30.

50. Jenkins LS, Nunn WD. 1987. Genetic and molecular characterization of the genes involved in short-chain fatty acid degradation in *Escherichia coli*: the *ato* system. J Bacteriol 169:42–52.

51. Steele MI, Lorenz D, Hatter K, Park A, Sokatch JR. 1992. Characterization of the *mmsAB* operon of *Pseudomonas aeruginosa* PAO encoding methylmalonate-semialdehyde dehydrogenase and 3-hydroxyisobutyrate dehydrogenase. J Biol Chem 267:13585–13592.

52. Meyer D, Schneider-Fresenius C, Horlacher R, Peist R, Boos W. 1997. Molecular characterization of glucokinase from *Escherichia coli* K-12. J Bacteriol 179:1298–1306.

53. Daddaoua A, Molina-Santiago C, de la Torre J, Krell T, Ramos J-L. 2014. GtrS and GltR form a two-component system: the central role of 2-ketogluconate in the expression of exotoxin A and glucose catabolic enzymes in *Pseudomonas aeruginosa*. Nucleic Acids Res 42:7654–7665.

54. Temple L, Sage A, Christie GE, Phibbs PV. 1994. Two genes for carbohydrate catabolism are divergently transcribed from a region of DNA containing the *hexC* locus in *Pseudomonas aeruginosa* PAO1. J Bacteriol 176:4700–4709.

55. Ghssein G, Brutesco C, Ouerdane L, Fojcik C, Izaute A, Wang S, Hajjar C, Lobinski R, Lemaire D, Richaud P, Voulhoux R, Espaillat A, Cava F, Pignol D, Borezée-Durant E, Arnoux P. 2016. Biosynthesis of a broad-spectrum nicotianamine-like metallophore in *Staphylococcus aureus*. Science 352:1105–1109.

56. Grim KP, San Francisco B, Radin JN, Brazel EB, Kelliher JL, Párraga Solórzano PK, Kim PC, McDevitt CA, Kehl-Fie TE. 2017. The Metallophore Staphylopine Enables *Staphylococcus aureus* To Compete with the Host for Zinc and Overcome Nutritional Immunity. mBio 8:e01281-17.

57. Burke KA, Lascelles J. 1975. Nitrate reductase system in *Staphylococcus aureus* wild type and mutants. J Bacteriol 123:308–316.

58. Huang Y, Lemieux MJ, Song J, Auer M, Wang D-N. 2003. Structure and mechanism of the glycerol-3-phosphate transporter from *Escherichia coli*. Science 301:616–620.

59. Riordan JT, Muthaiyan A, Van Voorhies W, Price CT, Graham JE, Wilkinson BJ, Gustafson JE. 2007. Response of *Staphylococcus aureus* to Salicylate Challenge. J Bacteriol 189:220–227.

60. Singh VK, Moskovitz J. 2003. Multiple methionine sulfoxide reductase genes in *Staphylococcus aureus*: expression of activity and roles in tolerance of oxidative stress. Microbiology (Reading) 149:2739–2747.

61. Wielders CLC, Fluit AC, Brisse S, Verhoef J, Schmitz FJ. 2002. *mecA* Gene Is Widely Disseminated in *Staphylococcus aureus* Population. J Clin Microbiol 40:3970.

62. Murchan S, Aucken HM, O'Neill GL, Ganner M, Cookson BD. 2004. Emergence, Spread, and Characterization of Phage Variants of Epidemic Methicillin-Resistant *Staphylococcus aureus* 16 in England and Wales. J Clin Microbiol 42:5154–5160.

63. Roman-Rodriguez F, Kim J, Parker D, Boyd JM. 2025. An effective response to respiratory inhibition by a *Pseudomonas aeruginosa* excreted quinoline promotes *Staphylococcus aureus* fitness and survival in co-culture. bioRxiv https://doi.org/10.1101/2025.03.12.642861.

64. Kehl-Fie TE, Zhang Y, Moore JL, Farrand AJ, Hood MI, Rathi S, Chazin WJ, Caprioli RM, Skaar EP. 2013. MntABC and MntH contribute to systemic *Staphylococcus aureus* infection by competing with calprotectin for nutrient manganese. Infect Immun 81:3395–3405.

65. Crosby HA, Tiwari N, Kwiecinski JM, Xu Z, Dykstra A, Jenul C, Fuentes EJ, Horswill AR. 2020. The *Staphylococcus aureus* ArlRS two-component system regulates virulence factor expression through MgrA. Mol Microbiol 113:103–122.

66. Radin JN, Kelliher JL, Solórzano PKP, Kehl-Fie TE. 2016. The two-component system ArlRS and alterations in metabolism enable *Staphylococcus aureus to* resist calprotectin-induced manganese starvation. PLoS Pathog 12:e1006040.

67. Walker JN, Crosby HA, Spaulding AR, Salgado-Pabón W, Malone CL, Rosenthal CB, Schlievert PM, Boyd JM, Horswill AR. 2013. The *Staphylococcus aureus* ArlRS Two-Component System Is a Novel Regulator of Agglutination and Pathogenesis. PLoS Pathog 9:e1003819.

68. Makhlin J, Kofman T, Borovok I, Kohler C, Engelmann S, Cohen G, Aharonowitz Y. 2007. *Staphylococcus aureus* ArcR controls expression of the arginine deiminase operon. J Bacteriol 189:5976–5986.

69. Wood JM. 1988. Proline porters effect the utilization of proline as nutrient or osmoprotectant for bacteria. J Membr Biol 106:183–202.

70. Schwan WR, Lehmann L, McCormick J. 2006. Transcriptional Activation of the *Staphylococcus aureus putP* Gene by Low-Proline-High Osmotic Conditions and during Infection of Murine and Human Tissues. Infect Immun 74:399.

71. Pelz A, Wieland K-P, Putzbach K, Hentschel P, Albert K, Götz F. 2005. Structure and Biosynthesis of Staphyloxanthin from *Staphylococcus aureus*. J Biol Chem 280:32493–32498.

72. Hammer ND, Reniere ML, Cassat JE, Zhang Y, Hirsch AO, Indriati Hood M, Skaar EP. 2013. Two Heme-Dependent Terminal Oxidases Power *Staphylococcus aureus* Organ-Specific Colonization of the Vertebrate Host. mBio 4:e00241-13.

73. Vaish M, Price-Whelan A, Reyes-Robles T, Liu J, Jereen A, Christie S, Alonzo F, Benson MA, Torres VJ, Krulwich TA. 2018. Roles of *Staphylococcus aureus* Mnh1 and Mnh2 Antiporters in Salt Tolerance, Alkali Tolerance, and Pathogenesis. J Bacteriol 200:e00611-17.

74. Troitzsch A, Loi VV, Methling K, Zühlke D, Lalk M, Riedel K, Bernhardt J, Elsayed EM, Bange G, Antelmann H, Pané-Farré J. 2021. Carbon Source-Dependent Reprogramming of Anaerobic Metabolism in *Staphylococcus aureus*. J Bacteriol 203:e00639-20.

75. Filkins LM, Graber JA, Olson DG, Dolben EL, Lynd LR, Bhuju S, O'Toole GA. 2015. Coculture of *Staphylococcus aureus* with *Pseudomonas aeruginosa* drives *S. aureus* towards fermentative metabolism and reduced viability in a cystic fibrosis model. J Bacteriol 197:2252–2264.

76. Jorge AM, Schneider J, Unsleber S, Xia G, Mayer C, Peschel A. 2018. *Staphylococcus aureus* counters phosphate limitation by scavenging wall teichoic acids from other staphylococci via the teichoicase GlpQ. J Biol Chem 293:14916–14924.

77. Jorge AM, Schneider J, Unsleber S, Göhring N, Mayer C, Peschel A. 2017. Utilization of glycerophosphodiesters by *Staphylococcus aureus*. Mol Microbiol 103:229–241.

78. Rolfsmeier M, Haseltine C, Bini E, Clark A, Blum P. 1998. Molecular Characterization of the α-Glucosidase Gene (*malA*) from the Hyperthermophilic Archaeon *Sulfolobus solfataricus*. J Bacteriol 180:1287–1295.

79. Hiron A, Posteraro B, Carrière M, Remy L, Delporte C, La Sorda M, Sanguinetti M, Juillard V, Borezée-Durant E. 2010. A nickel ABC-transporter of *Staphylococcus aureus* is involved in urinary tract infection. Mol Microbiol 77:1246–1260.

80. Bohn C, Rigoulay C, Chabelskaya S, Sharma CM, Marchais A, Skorski P, Borezée-Durant E, Barbet R, Jacquet E, Jacq A, Gautheret D, Felden B, Vogel J, Bouloc P. 2010. Experimental discovery of small RNAs in *Staphylococcus aureus* reveals a riboregulator of central metabolism. Nucleic Acids Res 38:6620–6636.

81. Rice K, Peralta R, Bast D, de Azavedo J, McGavin MJ. 2001. Description of staphylococcus serine protease (*ssp*) operon in *Staphylococcus aureus* and nonpolar inactivation of *sspA*-encoded serine protease. Infect Immun 69:159–169.

82. Videira MAM, Lobo SAL, Sousa FL, Saraiva LM. 2020. Identification of the sirohaem biosynthesis pathway in *Staphylococcus aureus*. FEBS J 287:1537–1553.

83. Vermilyea DM, Crocker AW, Gifford AH, Hogan DA. 2021. Calprotectin-mediated zinc chelation inhibits *Pseudomonas aeruginosa* protease activity in cystic fibrosis sputum. J Bacteriol 203:e00100-21.

84. Magalhães AP, França Â, Pereira MO, Cerca N. 2019. RNA-based qPCR as a tool to quantify and to characterize dual-species biofilms. Sci Rep 9:13639.

85. Menard G, Silard C, Suriray M, Rouillon A, Augagneur Y. 2022. Thirty Years of sRNA-Mediated Regulation in *Staphylococcus aureus*: From Initial Discoveries to In Vivo Biological Implications. Int J Mol Sci 23:7346.
